# Transcriptome Sequencing and iTRAQ of Different Rice Cultivars Provide Insight into Molecular Mechanisms of Cold-Tolerance Response in *Japonica* Rice

**DOI:** 10.1186/s12284-020-00401-8

**Published:** 2020-06-22

**Authors:** Yan Jia, Hualong Liu, Zhaojun Qu, Jin Wang, Xinpeng Wang, Zhuoqian Wang, Liang Yang, Dong Zhang, Detang Zou, Hongwei Zhao

**Affiliations:** 1 Key Laboratory of Germplasm Enhancement, Physiology and Ecology of Food Crops in Cold Region, Ministry of Education, Northeast Agriculture University, Harbin, 150030 Heilongjiang China; 2 Bei Da Huang Kenfeng Seed Limited Company, Harbin, 150431 Heilongjiang China; 3 PlantTech Biotechnology Co., Ltd., Beijing, 100000 China

**Keywords:** *Japonica* rice, Cold tolerance, Transcriptome, Proteome

## Abstract

**Background:**

Rice (*Oryza sativa* L.) is one of the most important crops cultivated in both tropical and temperate regions. However, it has a high sensitivity to cold stress and chilling stress limits its nitrogen uptake and metabolism. To identify the genes and pathways involved in cold tolerance, specifically within nitrogen metabolism pathways, we compared gene and protein expression differences between a cold-tolerant cultivar, Dongnong428 (DN), and a cold-sensitive cultivar, Songjing10 (SJ).

**Results:**

Using isobaric tags for relative or absolute quantification (iTRAQ) with high-throughput mRNA sequencing (RNA-seq) techniques, we identified 5549 genes and 450 proteins in DN and 6145 genes and 790 proteins in SJ, which were differentially expressed during low water temperature (T_w_) treatments. There were 354 transcription factor (TF) genes (212 downregulated, 142 upregulated) and 366 TF genes (220 downregulated, 146 upregulated), including 47 gene families, differentially expressed in DN under control (CKDN) vs. DN under low-T_w_ (D15DN) and SJ under control (CKSJ) vs. SJ under low-T_w_ D15SJ, respectively. Genes associated with rice cold-related biosynthesis pathways, particularly the mitogen-activated protein kinase (MAPK) signaling, zeatin biosynthesis, and plant hormone signal transduction pathways, were significantly differentially expressed in both rice cultivars. Differentially expressed proteins (DEPs) associated with rice cold-related biosynthesis pathways, and particularly glutathione metabolism, were significantly differentially expressed in both rice cultivars. Transcriptome and proteome analysis of the nitrogen metabolism pathways showed that major genes and proteins that participated in γ-aminobutyric acid (GABA) and glutamine synthesis were downregulated under cold stress.

**Conclusion:**

Cold stress conditions during reproductive growth, resulted in genes and proteins related to cold stress biosynthesis pathways being significantly differentially expressed in DN and SJ. The present study confirmed the known cold stress-associated genes and identified new putative cold-responsive genes. We also found that translational regulation under cold stress plays an important role in cold-tolerant DN. Low-T_w_ treatments affected N uptake and N metabolism in rice, as well as promoted Glu metabolism and the synthesis of ornithine and proline in cold-sensitive SJ.

## Background

Rice (*Oryza sativa* L.) is one of the most important crops cultivated in both tropical and temperate regions but it has a high sensitivity to cold stress. Cold temperatures are known to cause huge agricultural losses and are one of the most important environmental factors that affect rice growth and development (Ding et al. 2019). Chilling stress (0–20 °C) is the primary cold stress in tropical and subtropical climatic zones (Xu et al. 2008; Suh et al. 2010), especially in northeastern China, in parts of northern Japan (Shimono et al. 2007b), and Australia (Farrell et al. 2006). It is known to negatively influence the yield and quality of important food crops, such as rice (Shimono et al. 2002; Suzuki et al. 2008; Thakur et al. 2010; Wang et al. 2013; Zhao et al. 2013). Several studies have shown that the reproductive stage is the most sensitive period for cold damage (Jia et al. 2015; Wang et al. 2013; Sipaseuth et al. 2007; Jacobs and Pearson 1999; Shimono et al. 2007a). Furthermore, low root zone temperatures are known to inhibit the occurrence and elongation of functional roots, and this affects rice growth (Ahamed et al. 2012; Kramer and Boyer 1995). However, there is still no clear understanding of the response mechanisms of rice roots to low temperature stress during the reproductive stage.

The key challenge for crops is to maintain an adequate nutrition supply under abiotic stress conditions (Mcallister et al. 2012). A previous study demonstrated that there are many metabolic changes that occur in *Arabidopsis* to enhance freezing tolerance, which involve processes such as the Calvin cycle, nitrogen metabolism, starch synthesis, and sugar synthesis (Stitt and Hurry 2002). Nitrogen (N) plays an integral role in plant growth and development (Kraiser et al. 2011; Mcallister et al. 2012). Previously, the inhibited growth of *desA* and *desB* mutants of *Synechocystis* at decreased temperatures, was found to correlate with the inhibition of nitrate uptake (Sakamoto and Bryant 1999), and was relieved by the application of urea (Sakamoto and Bryant 1999). In rice, chilling stress has been found to generally limit N uptake (Zia et al. 1994; Shimazaki et al. 1963; Lu et al. 2005), and specifically, water temperature, not air temperature, is the main environmental factor regulating this and growth (Matsushima et al. 1964a; Matsushima et al. 1964b). Several other studies have also shown that low water temperature (T_w_) stress reduces N uptake (Shimazaki et al. 1963; Shimono et al. 2012), and this may be due to the reduced activity of enzymes and transporters under low root temperature water conditions (Feng et al. 2011).

Glutamic acid (Glu) plays a central role in the amino acid metabolism of plants (Seifi et al. 2013), which may orchestrate crucial metabolic functions under abiotic stress, such as the biosynthesis of GABA and proline (Pro), that have key roles in plant defense mechanisms (Forde and Lea 2007; Kishor et al. 2014; Hayat et al. 2012; Zhao et al. 2013). Cold stress was found to increase the levels of free amino acids, such as GABA, Glu, and Pro, in barley and wheat. (Mazzucotelli et al. 2006; Kovács et al. 2011). A number of previous studies have examined the GABA shunt response to abiotic stress (Kinnersley and Turano 2000; Bouche et al. 2003; Miyashita and Good 2008; Song et al. 2010). As osmotic regulators, GABA and Pro were found to reduce the oxidative damage to crops in abiotic stress conditions (Stitt and Hurry 2002; Bouche and Fromm 2004; Fait et al. 2008). These results indicate that GABA and Pro may have the potential to enhance the cold tolerance of plants. Considering these findings, the acclimation of nitrogen metabolism may be an essential prerequisite for the adaptation and performance of plants in low temperatures. However, the mechanisms of N metabolism in the roots of rice under low T_w_ conditions during their reproductive stage, are not yet completely understood.

A number of studies have shown that the mitogen-activated protein kinase (MAPK) signaling pathway plays a beneficial role in plants exposed to cold conditions (Shi et al. 2018; Nakagami et al. 2005; Zhu 2016). Homologous genes of ionotropic glutamate receptors (iGluRs) exist in rice (Singh et al. 2014), and glutamate receptor-like (GLR) channels may be essential components of plant signal transduction pathways, affecting the plants metabolic levels during stress conditions (Forde et al. 2013; Michard et al. 2011; Vincill et al. 2013; Li et al. 2006; Mousavi Seyed et al. 2013). Moreover, GLR channels had a significant effect on MAPK activation and the accumulation of defense gene transcripts in *Arabidopsis* plants (Kwaaitaal et al. 2011). Transcription factors (TFs) also play important roles in plant responses to low temperatures (Chinnusamy et al. 2007), and specifically, the WRKY TFs are involved in the MAPK signaling pathway (Asai et al. 2002). Likewise, *OsWRKY71*, which is a cold-responsive rice WRKY gene, acts as a repressor for *Glutamate decarboxylase* (Os03g0236200) (Kim et al. 2016). Furthermore, WRKY genes also regulate Pro biosynthesis under stress conditions. The transcription factor FcWRKY40 positively regulates Pro biosynthesis by directly regulating *SOS* and *P5CS1* homologs under salt stress (Dai et al. 2018). *CsWRKY46* confers cold resistance to transgenic plants by regulating Pro accumulation and a set of cold-stress responsive genes in an ABA-dependent manner (Zhang et al. 2016). There is ample evidence that many of the metabolites from glutamate metabolism are involved in plant responses to low temperature stress, though their reconfiguration under stress conditions is complex, and involves multiple molecular pathways (Guy et al. 2008; Hamid et al. 2013; Zhao et al. 2013; Dai et al. 2018; Kim et al. 2016; Zhang et al. 2016).

RNA sequencing (RNA-Seq) and MS-based shotgun proteomics are powerful high-throughput technologies for identifying and quantifying RNA transcripts and proteins, respectively (Wang et al. 2014). Transcriptome and proteome analysis of gene and protein expression, respectively, has already contributed greatly to our understanding of cold stress and identified a large number of cold-responsive genes in rice (Zhang et al. 2018; Sperotto et al. 2018; Cen et al. 2018; Ji et al. 2017). RNA-seq provides comprehensive information on mRNA abundance, alternative splicing, nucleotide variation, and structure alterations. Meanwhile, proteomic data provides essential confirmation of the validity and functional relevance of the novel findings from RNA-seq data; as the mRNA expression levels do not necessarily directly correspond to the abundance of the corresponding protein species. The relationship between protein and mRNA expression levels provides information on the combined outcomes of translation and protein degradation, which are, in addition to transcription and mRNA stability, essential contributors to gene expression regulation (de Sousa Abreu et al. 2009; Maier et al. 2009). Combined analysis of the transcriptome and proteome can generate complementary data, which when integrated appropriately, could potentially accelerate biological discoveries (Wang et al. 2014).

In this study, we explored the molecular mechanisms involved in rice cold tolerance, using high-throughput mRNA sequencing (RNA-seq) and isobaric tags for relative and absolute quantification (iTRAQ), to analyze the transcriptome and proteome, respectively. The cold-tolerant rice cultivar DN and the cold-sensitive cultivar SJ were exposed to either low T_w_ or control treatments for 15 days, during the reproductive stage of growth. We compared the mRNA and protein expression profiles of the cultivars from the control and cold stress treatments, which demonstrated low expression correlation. We determined the functions of differentially expressed genes (DEGs) and proteins (DEPs) using enrichment analysis of the gene ontology (GO) and the Kyoto Encyclopedia of Genes and Genomes (KEGG) databases. We then compared transcriptome and proteome data between the different rice cultivars under normal and cold stress conditions. We selected the DEGs with inverse change trends and analyzed the nitrogen metabolism responses of rice during the low-T_w_ treatment, to provide information on the association which will provide an association between the nitrogen metabolism pathway and cold tolerance in rice.

## Results

### Phenotypic and Physiological Responses of Rice during Cold Stress

**Fig. 1:**
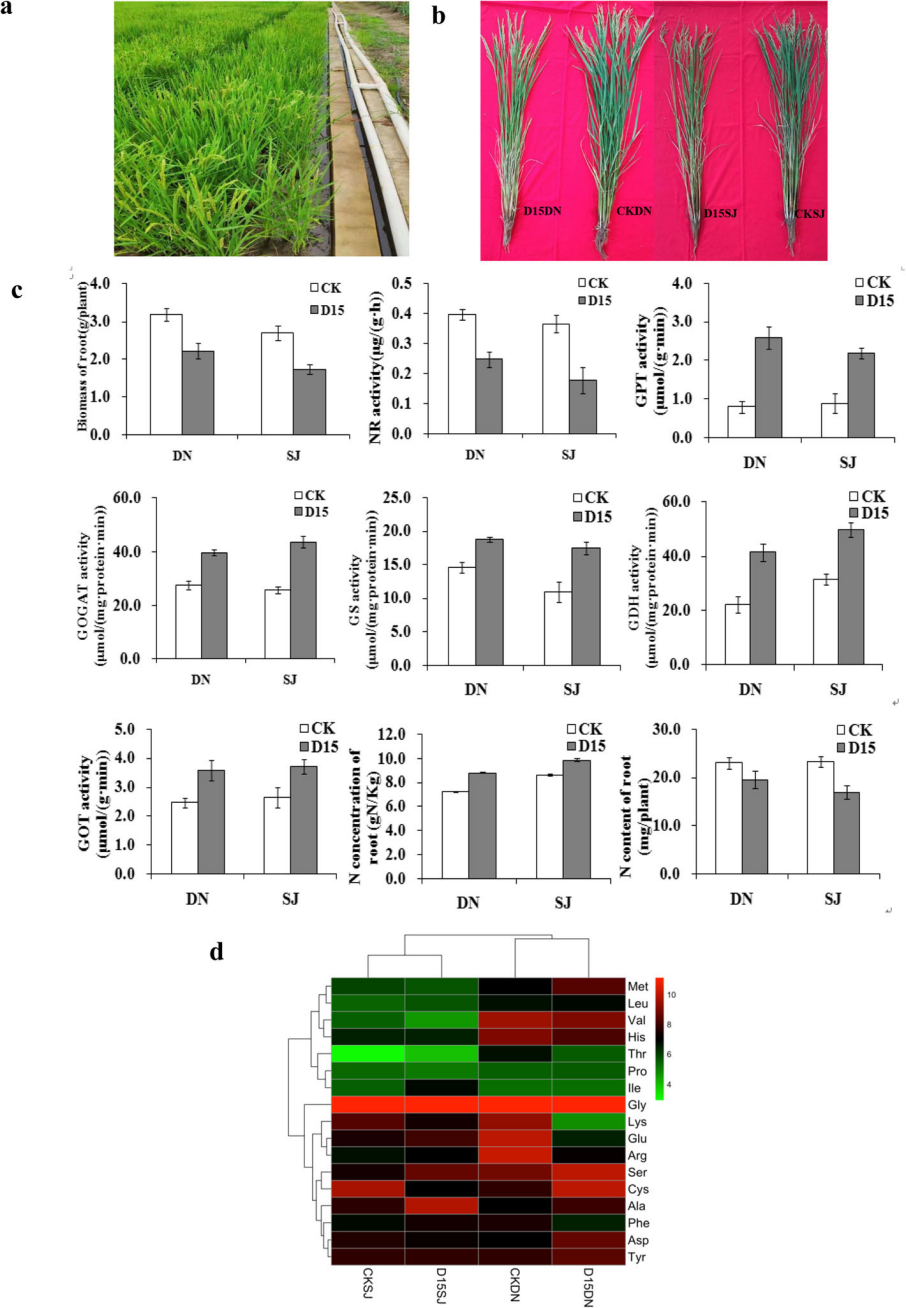
Phenotypic and physiological responses of two rice cultivars with low-T_w_ treatment during reproductive growth. **a.** The plots of cold water treatment in the field **b,** Plants of Dongnong428 (DN) and Songjing10 (SJ) cultivars under low-T_w_ treatment at the full heading stage. **c,** Nitrogen (N) concentration, and nitrate reductase (NR), glutamate synthase (GOGAT), glutamine synthetase (GS), glutamate dehydrogenase (GDH), aspartate aminotransferase (GOT), and alanine aminotransferase (GPT) activities in the control (CK) plants and after 15 d of the low-T_w_ treatment. **d,** Differences in the amino acid contents of the CK and 15 d low-T_w_ treatments of DN and SJ

The cold-stress phenotypes of rice are often too unstable to be surveyed in the field. To precisely measure the cold tolerant phenotypes of rice, we used 150 in situ plots for cold water treatments (Fig. 1a). Each plot was 9 m^2^ and with 90 individual rice plants (Fig. 1a). We selected 50 widely planted *japonica* rice cultivars with similar flowering times to measure their phenotypic responses to cold stress at the reproductive stage. For each cultivar, we grew 1620 individual plants in six plots, with an approximately 20 cm water layer depth. When the plants reached their reproductive stage, with approximately 1 cm young panicles (da Cruz et al. 2006), we independently treated three plots with 17 °C low- temperature water for 15 days, which is close to the temperature that causes cold damage to rice during reproductive growth in Heilongjiang Province, China. The other three plots of rice served as controls. In total, we systematically measured the phenotypes of 81,000 individual plants. We identified 21 cold-tolerant cultivars and 29 cold-sensitive cultivars. Compared with those of the control, the cold-sensitive cultivars had significantly reduced yields and biomass, whereas those of the cold-tolerant cultivars were only slightly reduced (Fig. 1a).

We selected the cold-tolerant cultivar Dongnong428 (DN) and cold-sensitive cultivar Songjing 10 (SJ) to utilize in the following experiments. The yield of DN under control (CKDN) was 880 g/m^2^ and that of SJ under control CKSJ was 810 g/m^2^. The 15 d low-T_w_ treatment decreased the yields in comparison with those of the controls, by 41% for DN under low-T_w_ (D15DN) (515 g/m^2^) and 51% for DN under low-T_w_ (D15SJ) (395 g/m^2^) (Fig. 1b).

We measured the physiological indicators and nitrogen content of the samples that experienced chilling damage. Compared with the untreated samples, the nitrate reductase (NR) activity of the roots was significantly decreased in D15DN (37.8%) and D15SJ (51.5%), while the glutamate synthase (GOGAT), glutamine synthetase (GS), glutamate dehydrogenase (GDH), aspartate aminotransferase (GOT), and alanine aminotransferase (GPT) activities were significantly increased. This indicated that under the low-T_w_ treatment, the NO_3_^−^ reducing ability in the roots of the rice was decreased, but the NH_4_^+^ assimilation ability was enhanced, which was the main reason for the increased N concentration in the roots (21.6%, DN; 14.1%, SJ). However, with the low-T_w_ treatment, the biomass of the roots was significantly decreased (30.4%, DN; 36.4%, SJ), which lead to decreased levels of N in the roots (15.4%, DN; 27.4%, SJ) (Fig. 1c).

Amino acid content analysis showed that the glutamate acid (Glu) content in D15DN was reduced and the Pro content was slightly increased, compared with that of CKDN. In D15SJ, the content of the Glu increased and the content of the Pro decreased slightly, compared with that of CKSJ. Compared with D15SJ, the Glu content in D15DN was 64.7% lower, while the Pro content was 42.9% higher (Fig. 1d).

### Genome-Wide Expression Changes in mRNA and Protein Expression under Cold Stress

There were significant phenotypic differences in the dry matter accumulation and N metabolism related physiological indicators of the roots, between DN and SJ (Fig. 1c). To understand the regulatory mechanisms of the cold stress and explore new regulators, cold responsive transcriptome, and proteome changes in the roots of DN and SJ were studied using RNA-seq and iTRAQ. Four samples (CKDN; CKSJ; D15DN; D15SJ) with three biological replicates each, were used for transcriptome analysis. Then, 12 cDNA libraries were prepared from these samples and subjected to paired-end sequencing. Gene expression was calculated as the fragments per kilobase per million reads (FPKM). For RNA-seq, each library ranged from 31.40 to 46.94 million reads. We mapped the clean reads onto the reference genome via HISAT (hierarchical indexing for spliced alignment of transcripts) (Kim et al. 2015) and most of the clean reads (73.02–88.02%) for each library were perfectly mapped to the rice reference genome. The reads that could not be mapped to the rice genome were discarded, and only the mapped reads were analyzed further. FPKM was calculated to measure the expression levels of the transcripts and showed high correlations (spearman correlation coefficient (SCC) = 0.84–0.98) among the biological replicates. A total of 43,704 transcripts were detected in at least one sample and 12,610 new transcripts were found, compared with the reference genome.

Changes in the proteomes of the roots were quantitatively cataloged using iTRAQ. There were three biological replicates for each sample in the proteome analysis. The search algorithm Mascot was used to identify the proteins (Hirosawa et al. 1993). There were 1,184,867 spectra generated from the roots of the two rice varieties. A total of 265,517 of the spectra were matched to known protein sequences and 212,755 spectra were uniquely matched. There were also 11,666 mapped peptides, 32,750 mapped unique peptides, and 7281 mapped proteins, with a 1% false discovery rate (FDR). Repeatability analysis based on the coefficient of variation (CV) revealed that more than 95% of the proteins could be found in the total identified proteins, when the CV value was less than 50%.

**Fig. 2:**
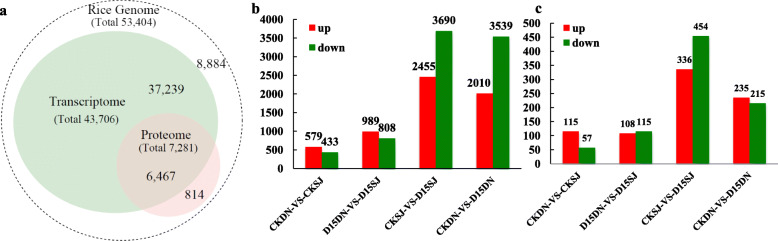
Comparison of the protein and transcript abundances in the roots of the two rice cultivars. **a**, Congruency between the detected transcripts and the proteins of the rice endosperm. **b,** Number of differentially expressed proteins in the roots (1.2-fold change with P value < 0.05). **c,** Number of differentially expressed genes in the roots (absolute value of log2 (FC ≥1) with FDR ≤0.01)

Based on the iTRAQ results, 88.8% of the proteins were detected in the transcriptome data, accounting for 14.8% of the total number of transcripts detected (Fig. 2a). To explore the relationship between the proteins and their corresponding genes, we calculated spearman correlations for each group. The comparisons revealed a low concordance between the data obtained from the two technologies (spearman correlation coefficient between − 0.07 and 0.10) (Figure S1).

To explore the relationship between the proteins and their corresponding genes, we matched all expressed proteins with their corresponding genes in each treatment and observed a weak correlation, with *r* values ranging from 0.14 to 0.31. These results demonstrate that gene expression cannot fully account for the protein species abundances, and strong post-translational regulations may exist in the process of protein production.

### Cold Response Transcriptome Differences in Rice Cultivars

Differential gene expression was examined using the R package DEseq2 to quantify and analyze all control and treatment combinations (Love et al. 2014). Over the 15 d of cold exposure, a total of 5549 genes were differentially expressed in the roots between D15DN and CKDN, of which 3539 genes were downregulated and 2010 upregulated. There were 6145 genes that were differentially regulated in the roots between D15SJ and CKSJ, of which 3690 were downregulated and 2455 were upregulated (Fig. 2b). The number of differentially expressed genes in the two varieties of rice was similar after the cold treatments, indicating that there is some similarity between the two varieties in their responses to cold stress.

To determine the plant systems influenced during the cold stress, the DEGs were searched against the GO database for enrichment analysis. The GO database enrichment analysis was separated into three ontologies: molecular function (MF), cellular component (CC), and biological process (BP); 19 and 16 GO terms were significantly different (*P* ≤ 0.05) in the CKDN vs. D15DN and CKSJ vs. D15SJ comparisons, respectively.

**Fig. 3:**
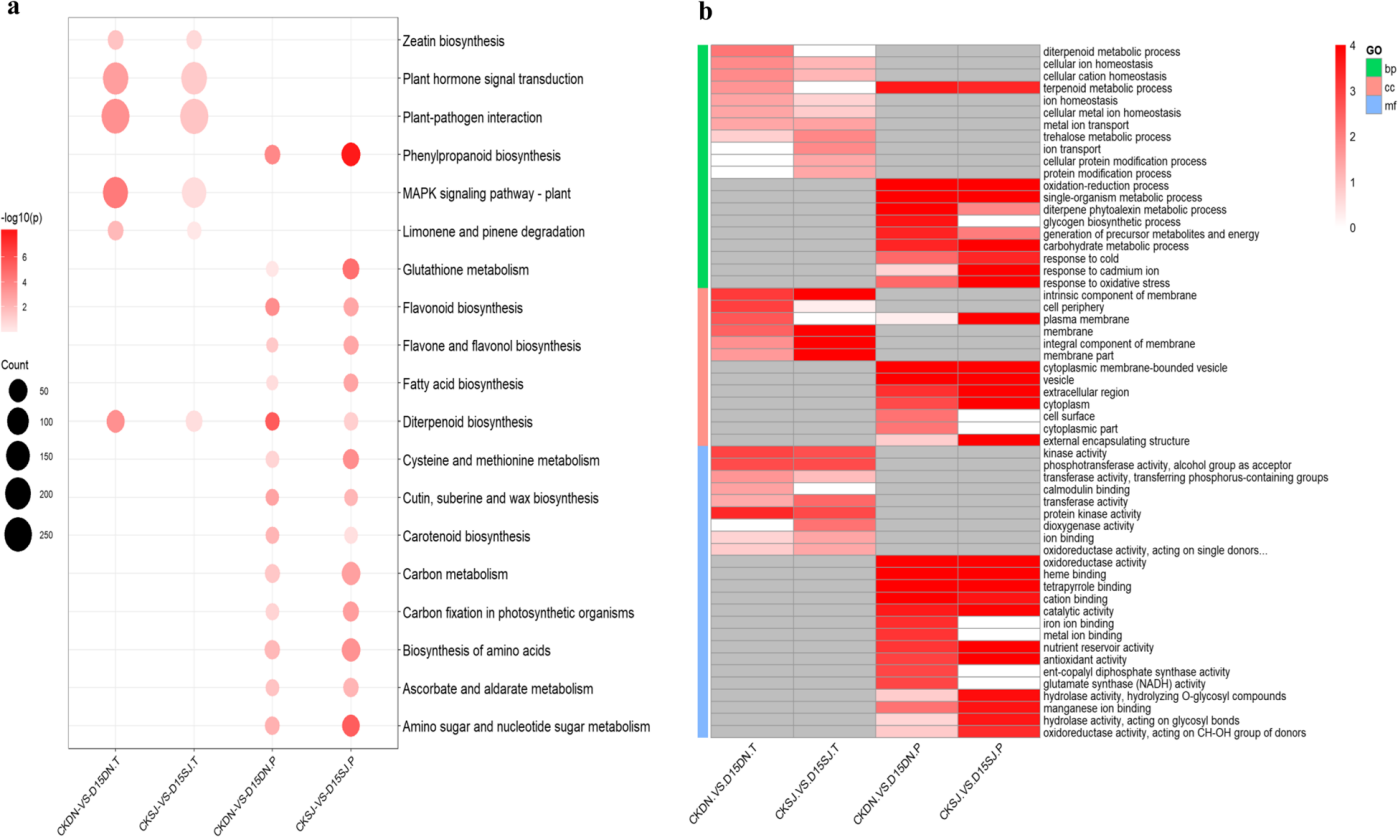
Enrichment analysis of the cold responsive transcriptome and proteome changes in the two rice cultivars. **a,** KEGG enrichment analysis of the differentially expressed genes and proteins presented in a bubble chart. **b,** GO enrichment analysis of the differentially expressed genes and proteins presented as a heat map

The DEGs of the CKDN vs. D15DN and CKSJ vs. D15SJ enriched terms focused on intrinsic components of the membrane and phosphotransferase activity (alcohol group as acceptor). The specific term enrichment for CKDN vs. D15DN included cell periphery and plasma membrane, calmodulin binding (as well as ion binding), diterpenoid metabolic process, cellular ion homeostasis, and metal ion transport. Oxidoreductase activity (acting on single donors with the incorporation of molecular oxygen incorporation of two atoms of oxygen), trehalose metabolic processes, ion transport, cellular protein modification processes, and protein modification were identified for CKSJ vs. D15SJ (Fig. 3b).

The Kyoto Encyclopedia of Genes and Genomes (KEGG) pathway enrichment analysis was conducted to determine the biological pathways involved in cold stress. Previous studies have demonstrated that there are many metabolic changes that occur in *Arabidopsis* to enhance its tolerance to freezing conditions, affecting areas such as the Calvin cycle, nitrogen metabolism, starch synthesis, and sugar synthesis (Stitt and Hurry 2002). By applying a cut-off criterion of a Q value ≤0.05, the results showed that six and one pathways were significantly enriched in the CKDN vs. D15DN and CKSJ vs. D15SJ groups, respectively (Fig. 3a).

Pathways enriched between CKDN vs. D15DN were those for the MAPK signaling pathway, diterpenoid biosynthesis, plant-pathogen interaction, plant hormone signal transduction, limonene and pinene degradation, and zeatin biosynthesis. While the pathway enriched between CKSJ vs. D15SJ was for plant-pathogen interactions.

A number of studies have shown that the MAPK signaling pathway has a beneficial role in plants exposed to cold conditions (Shi et al. 2018; Nakagami et al. 2005; Zhu 2016). There were 184 DEGs involved in the MAPK signaling pathway in CKDN vs. D15DN, of which only 164 were also DEGS in CKSJ vs. D15SJ (Fig. 3a). Twenty genes were specifically upregulated and 55 specifically downregulated in the MAPK signaling pathway in CKDN vs. D15DN, including two ethylene receptors (Cht9 and ERS2), three LRR receptor-like serine/threonine-protein kinases (Os01g0191200, 107,275,384, and 107,278,972), and two WRKY transcription factors (Os08g0500300 and 107,281,845).

### Cold Response Proteome Changes in Contrasting Rice Cultivars

A cut-off threshold of a 1.2-fold change for increased accumulations and a 0.83-fold change for decreased accumulations was applied, together with a number of unique peptides ≥2. Based on these criteria, a total of 450 proteins were differentially expressed in the roots between D15DN and CKDN, of which 215 were downregulated and 235 were upregulated. Between D15SJ and CKSJ, 790 proteins were differentially regulated in the roots, of which 454 were downregulated and 336 were upregulated (Fig. 2c).

Gene Ontology (GO) enrichment analysis was used to identify over-represented terms from the GO (Alexa and Rahenfhürer, 2009) ontologies for differentially expressed proteins. A full list of all the enriched terms with p values ≤0.05 can be found in Table S5.

The GO term enrichment analysis of the DEPs for CKDN vs. D15DN and CKSJ vs. D15SJ included oxidoreductase activity, heme binding, catalytic activity, cytoplasmic membrane-bound vesicle, oxidation-reduction process, diterpene phytoalexin metabolic process, response to cold, and response to oxidative stress. The specific terms of enrichment for CKDN vs. D15DN were iron ion binding, metal ion binding, ent-copalyl diphosphate synthase activity, glutamate synthase (NADH) activity, cell surface, and cytoplasmic glycogen biosynthetic process. For CKSJ vs. D15SJ, the terms of enrichment also included those related to cadmium ion, external encapsulating structure and plasma membrane, hydrolase activity, glycosyl bonds, oxidoreductase activity, and the CH-OH group of donors (Fig. 3b).

The KEGG pathways enriched in CKDN vs. D15DN and CKSJ vs. D15SJ were diterpenoid biosynthesis, phenylpropanoid biosynthesis, flavonoid biosynthesis, cutin, suberin, and wax biosynthesis, amino sugar and nucleotide sugar metabolism, biosynthesis of amino acid, ascorbate and aldarate metabolism, carbon metabolism, and flavone and flavonol biosynthesis. The specific pathway enrichments for CKDN vs. D15DN were carotenoid biosynthesis, as well as glutathione metabolism, cysteine and methionine metabolism, carbon fixation in photosynthetic organisms, and fatty acid biosynthesis in CKSJ vs. D15SJ (Fig. 3b).

There have been previous studies on the genes involved in diterpenoid biosynthesis and the phenylpropanoid biosynthesis pathways in response to cold stress (Fang et al. 2017). Diterpenoid biosynthesis was enriched in the transcriptome (39 genes) and the proteome (9 genes) data. Os04g0178400 (CYP99A3), Os12g0491800 (KSL10), Os02g0570700 (CYP71Z7), Os01g0561600, and Os04g0178300 (CPS4) were both upregulated in the transcriptome and proteome data.

### Combined Transcriptome and Proteome Analyses of the Differences in the Cold Response Mechanisms of the Two Rice Cultivars

**Fig. 4:**
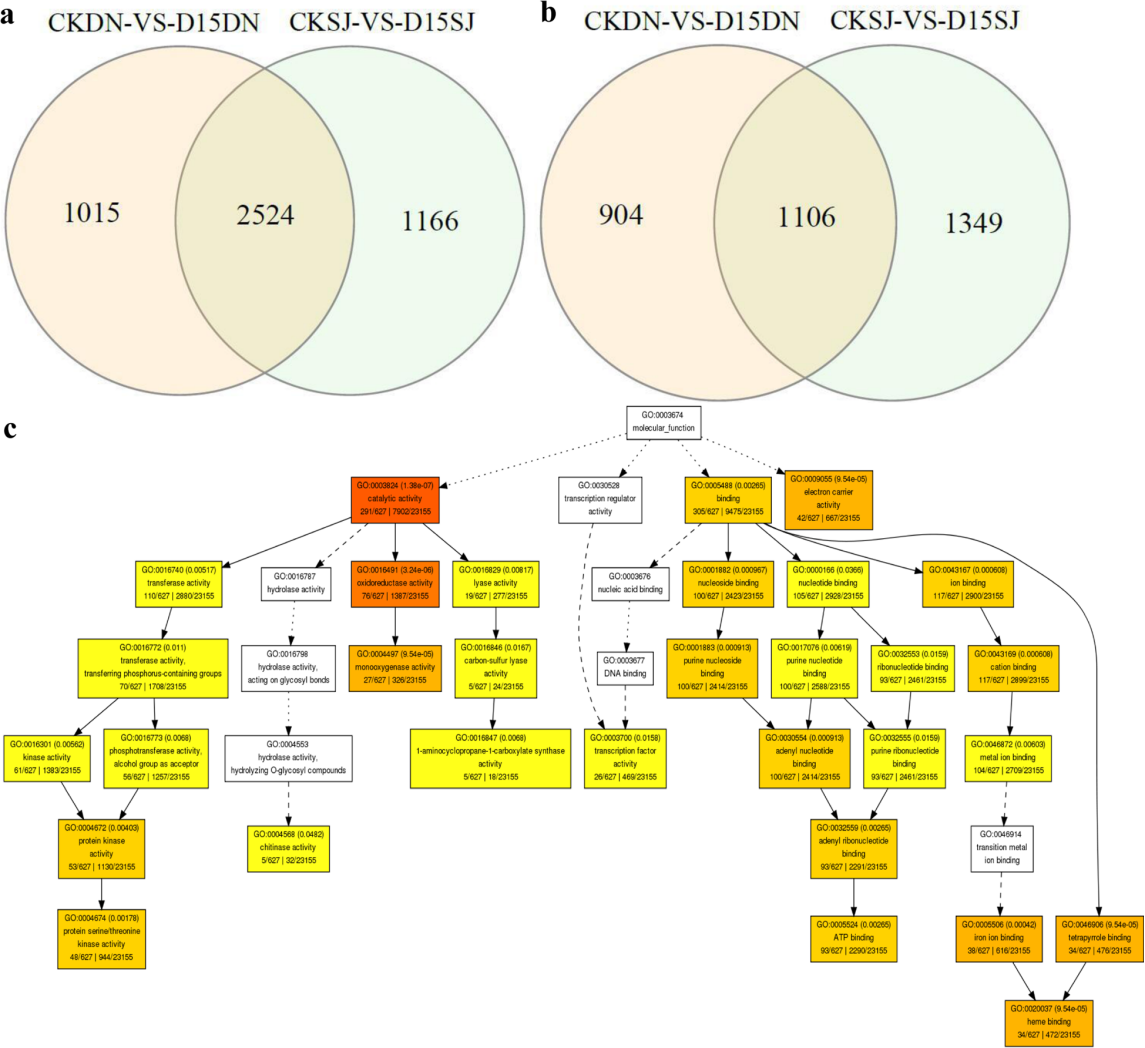
Comparative transcriptome analysis of the two cultivars revealed cold tolerance mechanisms. **a**, Venn diagram of the downregulated differentially expressed genes in Dongnong428 (DN) under control (CKDN) vs. DN under low-T_w_ (D15DN) and Songjing10 (SJ) under CKSJ vs. D15SJ. **b**, Venn diagram of the upregulated differentially expressed genes in CKDN vs. D15DN and CKSJ vs. D15SJ. **c,** GO enrichment analysis of the specific CKSJ vs. D15SJ differentially expressed genes

**Fig. 5:**
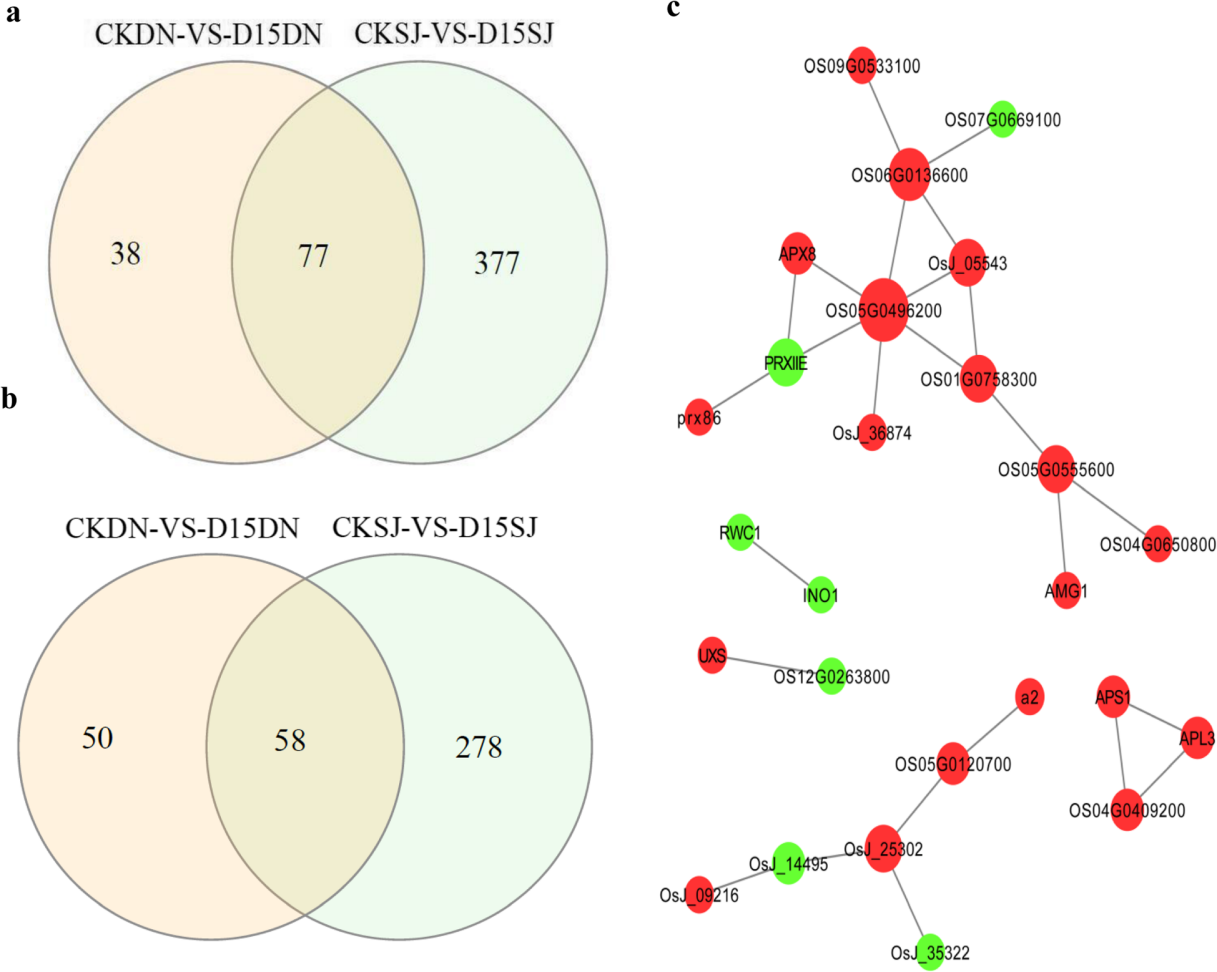
Comparative proteome analysis of the rice cultivars revealed cold tolerance mechanisms. **a,** Venn diagram of the downregulated differentially expressed proteins in Dongnong428 (DN) under control (CKDN) vs. DN under low-T_w_ (D15DN) and Songjing10 (SJ) CKSJ vs. D15SJ. **b,** Venn diagram of the upregulated differentially expressed proteins in CKDN vs. D15DN and CKSJ vs. D15SJ. **c,** Protein-protein interaction analysis of the specific CKSJ vs. D15SJ differentially expressed genes, performed using Cytoscape

Venn analysis showed that in the transcriptome data, 1015 DEGs were upregulated in the CKDN /D15DN group, and 904 were downregulated (Fig. 4a, b). Venn analysis showed that in the proteome data, only 38 DEPs were upregulated in the CKDN/D15DN group, and 50 were downregulated (Fig. 5a, b). There were only five upregulated and nine downregulated DEPs in the proteome and transcriptome data in the CKDN/D15DN group, and there were low spearman correlation coefficients between − 0.07 and 0.10, for each group (Figure S1), indicating strong post-translational regulation and complementarity of the transcriptome and proteome analysis. The upregulated genes contained an aspartyl protease family protein (Os06g0304600); tuliposide A-converting enzyme b6(Os09g0455900); probable polygalacturonase (Os06g0106800); and enolase 1(Os06g0136600); lichenase-2(Os05g0375400). The downregulated genes included FAR1; PRXIIE-2; CYP76M7; CXE15; CPS2; AIG2LD; ALDH2C4; uncharacterized isomerase BH0283 (Os01g0266500); and basic secretory protease (Os10g0491000). CYP76M7 is involved in oxidoreductase activity, terpenoid biosynthetic process, lipid metabolic process, phytoalexin metabolic process, and oxidation-reduction process. CPS2 is involved in magnesium ion binding, lyase activity, terpenoid biosynthetic process, lipid metabolic process, and phytoalexin metabolic process. Enolase 1 is involved in magnesium ion binding, lyase activity, oxidoreductase activity, and oxidation-reduction process; CYP76M7, CPS2, and enolase 1 may play important roles in resisting cold stress.

Combining DEGs and DEPs, we found 1048 upregulated and 945 downregulated genes in the CKDN/D15DN group (Fig. 4a, b). GO enrichment analysis of the upregulated and downregulated genes indicated responses to heat, electron transport chain, flavonoid glucuronidation, and inorganic anion transport. Seven genes were identified that are involved in the “response to heat”: Os06g0104100, Os03g0268400, Os01g0688900, Os07g0517100, Os05g0334000, Os01g0136100, and Os05g0519700, which may also participate in cold stress resistance. Eight genes were identified that are involved in “inorganic anion transport”: Os01g0645900, Os06g0324800, Os01g0704100, Os01g0547600, Os10g0420400, Os01g0304100, Os03g0196000, and Os07g0524000. The terms cellular component, chloroplast thylakoid membrane, chloroplast, ATP-binding cassette (ABC) transporter complex, plastid envelope, extracellular region, and microtubule cytoskeleton were also enriched. Os01g0836600 and Os01g0393400 were associated with the term ATP-binding cassette (ABC) transporter complex. In terms of molecular functions, polysaccharide binding, rRNA binding, protein tyrosine kinase activity, inorganic anion transmembrane transporter activity, quercetin 3-O-glucosyltransferase activity, O-acetyltransferase activity, terpene synthase activity, plant-type vacuole membrane, GTP binding, and protein serine/threonine kinase activity were represented. There were seven genes involved in “rRNAbinding”: Os03g0356300, Os02g0489400, Os12g0133050, Os03g0122200, Os08g0130500, Os07g0628400, and Os05g0270000 (Fig. 4c).

### Identification of Transcription Factors (TFs) that Responded to the Cold Treatments

**Fig. 6:**
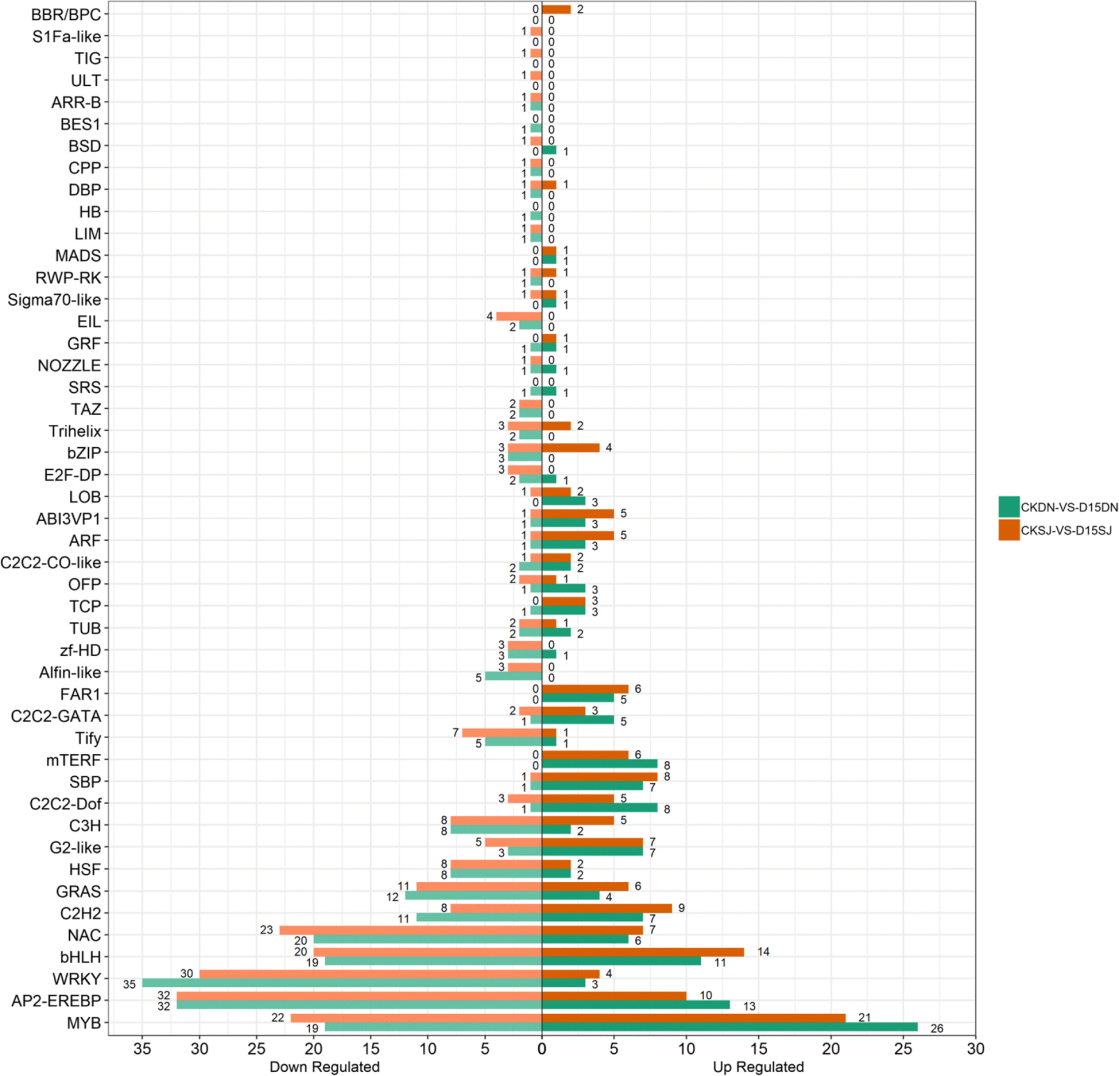
Identification of the transcription factors (TFs) responding to cold responses

Transcription factors (TFs) play important roles in plant responses to low temperatures (Chinnusamy et al. 2007), as they regulate downstream genes in response to cold stress. Identification of TF genes can provide insight into the molecular mechanisms of cold stress response systems and we found that 1869 TFs were expressed in at least one cultivar. There were 354 TF genes (212 down, 142 up), 366 TF genes (220 down, 146 up), including 47 gene families, differentially expressed in CKDN/D15DN and CKSJ/D15SJ, respectively (Fig. 6).

In the CKDN vs. D15DN group, there were 101 TFs (42 down, 59 up) that were differentially expressed. From the PlantRegMap database we found that 14 differentially expressed TFs had 2250 targeted genes and a total of 2831 targeted relationship pairs. Of these 14 TFs, 13 were upregulated and one was downregulated. Among the 2250 genes, 349 were differentially expressed in the CKDN vs. D15DN group, of which 164 were upregulated and 185 were downregulated. The 14 differentially expressed transcription factors and 349 differential genes, constituted 355 targeted relationship pairs.

The Dof family of transcription factors are one of the most important families of transcriptional regulators in higher plants and are involved in plant growth, development, and responses to abiotic stresses. The Os03g0821200, Os07g0685000, Os10g0406300, Os05g0112200, and Os12g0569900 genes, belonging to the C2C2-Dof family, were upregulated in the CKDN vs. D15DN group. Os03g0821200 and Os07g0685000 regulated 39 (19 upregulated, 20 downregulated) and 4 (3 upregulated, 1 downregulated) targeted genes, respectively. Auxin transport protein REH1 was upregulated and auxin-responsive protein IAA26 was downregulated, suggesting that the Dof family may adapt to cold stress by regulating auxin transport and responses.

ERF genes are involved in developmental regulation and environmental responses. The genes Os06g0194000 and Os03g0860100, which were upregulated in the CKDN vs. D15DN group, and Os08g0565200, which was downregulated, belong to the ERF family. The Os05g0403400 and Os02g0749900 genes were grouped with the GO terms for mitochondrion and nitrogen compound metabolic processes. Three ERF family genes controlled the expression of 328 genes (158 upregulated and 170 downregulated). Upregulated genes included GAUT14 (galacturonosyl transferase 14), GAUT7 (galacturonosyl transferase 7), ubiquitin-activating enzyme E1, and RING-H2 finger protein ATL5F. Downregulated genes included histone H2A, cytochrome P450 CYP734A7, delta 12 oleic acid desaturase FAD2, and circadian factor ZGT.

The MYB family is large and involved in controlling various processes, such as responses to biotic and abiotic stresses, development, differentiation, metabolism, and defense. The Os08g0178900, Os05g0140100, Os11g0180900, Os08g0151300, Os07g0634900, Os01g0192300, Os01g0274800, Os02g0685200, Os09g0538400, Os01g0128000, Os11g0684000, Os06g0105800, and Os11g0207600 genes belonging to the MYB family were upregulated in the CKDN vs. D15DN group. Six MYB family members regulated 46 target genes (21 upregulated and 25 downregulated). Upregulated genes included thiamine thiazole synthase THI1, flavonoid 3′-monooxygenase CYP75B4, and downregulated genes include sugar transport protein MST5, actin-depolymerizing factor 3 ADF- 3, and bifunctional nuclease 1 (OsBBD1).

The WRKY transcription factors are one of the largest families of transcriptional regulators in plants. WRKY genes have been found to play significant roles in biotic and abiotic stress responses, and to also regulate growth and development. We found no targeted WRKY genes using the PlantRegMap database, but found putative novel ones in this investigation. The Os07g0111400, Os12g0597700, Os01g0586800, Os06g0146250, Os12g0116700, Os08g0235800, 107,277,882, Os11g0117500, Os11g0685700, Os06g0158100, and BGI_novel_G000104 genes belonging to the WRKY family, were upregulated in the CKDN vs. D15DN group. The Os07g0111400, Os12g0597700, Os01g0586800, Os06g0146250, Os08g0235800, Os11g0685700, Os06g0158100, and BGI_novel_G000104 genes are involved in the MAPK signaling pathway. The WRKY TFs were involved in the mitogen-activated protein kinase (MAPK) signaling pathway, which is involved in stress-induced defensive responses (Asai et al. 2002).

### Metabolism, Transcriptome, and Proteome Analyses of Nitrogen Metabolism Responses to Cold Stress

In the low-T_w_ treatment, the N content in the roots was lower than that of the control by 15.4% in DN and 27.4% in SJ. The amino acid content analysis showed that the Glu content in the D15DN was significantly lower and the Pro content was slightly higher than that of the control. In D15SJ, the glutamic acid content was higher and the Pro content was slightly lower, than that of the control. Compared with D15SJ, the glutamate content in D15DN was 64.7% lower, while the Pro content was 42.9% higher.

**Fig. 7:**
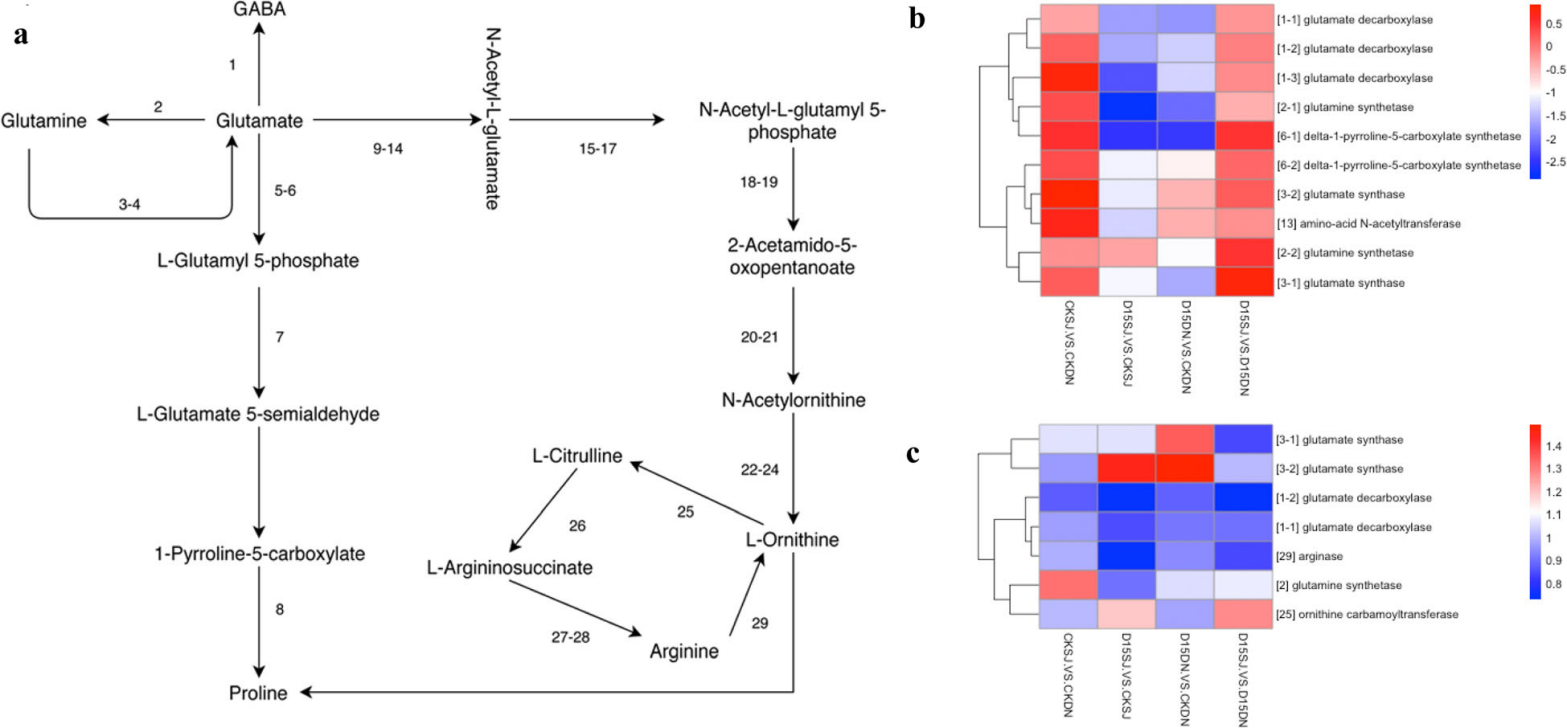
Expression differences in the amino acid metabolism pathways of the transcriptome and proteome. **a,** Amino acid metabolism pathway in KEGG pathway database. **b,** Differentially expressed genes in the amino acid metabolism pathway of the transcriptome. **c,** Differentially expressed proteins in the amino acid metabolism pathway of the proteome

Transcriptome analysis of the nitrogen metabolism pathways, identified nine genes that were downregulated in the D15SJ/CKSJ group (Fig. 7a, b), including three glutamate decarboxylase genes involved in GABA synthesis, two glutamine synthetase genes involved in glutamine synthesis, one glutamate synthase gene involved in glutamate synthesis from glutamine, two delta-1-pyrroline-5-carboxylate synthetase genes involved in L-Glutamyl 5-phosphate synthesis, and one amino-acid N-acetyltransferase gene involved in N-acetyl-L-glutamate synthesis. There were six genes that were downregulated in the D15DN/CKDN group, including three glutamate decarboxylase genes involved in GABA synthesis, two glutamine synthetase genes involved in glutamine synthesis, and one delta-1-pyrroline-5-carboxylate synthetase gene involved in L-glutamyl 5-phosphate synthesis. Compared with D15SJ, no genes showed significant upregulation or downregulation in D15DN.

Proteome analysis of the nitrogen metabolism pathways identified five proteins that were upregulated or downregulated in the D15SJ/CKSJ group (Fig. 7b, c), including two glutamate decarboxylase proteins involved in GABA synthesis and one arginase gene involved in L-ornithine synthesis from arginine were downregulated. One glutamate synthase gene involved in glutamate synthesis, and one ornithine carbamoyl transferase gene involved in L-ornithine synthesis were upregulated in the D15SJ-vs-CKSJ group. In the D15DN/CKDN group, two glutamate synthase genes involved in glutamate synthesis were upregulated. Compared with D15SJ, one ornithine carbamoyl transferase gene involved in L-ornithine synthesis showed significant upregulation in D15DN.

## Discussion

### Transcriptome Analysis cannot Fully Represent the Proteome Analysis

Comparative analyses of the mRNA and protein abundances in a steady state, and the expression alterations that occur with different environmental conditions, have demonstrated that transcription is only half the story (Plotkin 2010). Discordant mRNA and protein changes have revealed the genes that are responsible for the alterations in the protein-level regulations (Wang et al. 2014). In our study, 43,704 transcripts and 7281 proteins were detected in the transcriptome and proteome data, respectively. While transcriptome sequencing can detect more gene expression, more than 80% of the differentially expressed proteins were not differentially expressed in the transcriptome. We also matched expressed proteins with their corresponding genes in each treatment and observed a weak correlation, demonstrating that gene expression cannot fully represent the abundance of protein species, and strong post-translational regulations may exist in the process of protein species production.

### The Role of Transcription Factors in Rice Responses to Cold Stress

Plant tolerance to cold stress involves a complex regulatory network of TFs and other regulatory genes that control enzymes, regulatory proteins, and metabolites. In this study, genes from five TF families, including the C2C2-Dof, C2C2-GATA, WRKY, GRAS, and MtERF were identified as COR genes (Zhang et al. 2009). In previous studies, over-expressed plants such as *OsCOIN*, *OsMYB2*, *OsMYB4*, *OsMYB3R-2*, and *OsZFP245* showed significant increases in Pro content and enhanced their tolerance to low temperatures (Liu et al. 2007; Yang et al. 2012; Park et al. 2010; Vannini et al. 2004; Ma et al. 2009; Huang et al. 2009). In this study, compared with D15SJ, the Pro content in D15DN was increased by 42.9%. The Os08g0178900, *OsMYB2P-1*, Os11g0180900, *OsMYB103*, Os07g0634900, Os01g0192300, *CSA*, Os02g0685200, Os09g0538400, Os01g0128000, *OsJAMyb*, Os06g0105800, and Os11g0207600 genes belonging to the MYB family, were only upregulated in the CKDN vs. D15DN group. The expression of *OsMYB2P-1* has been shown to be induced by cold, salt, and osmotic stress conditions (Xu et al. 2014), indicating that *OsMYB2P-1* is associated with Pro accumulation in rice under low temperature stress.

The Dof family of transcription factors is one of the most important families of transcriptional regulators in higher plants, and is involved in plant growth, development, and responses to abiotic stresses. The *OsDof16*, *OsDof24*, *OsDof27*, *OsDof19*, and *OsDof29* genes belonging to the C2C2-Dof family were only upregulated in the CKDN vs. D15DN group.

The WRKY TFs are involved in the MAPK signaling pathway, which is involved in stress-induced defensive responses (Asai et al. 2002). In this study, the *OsWRKY29*, *OsWRKY83*, *OsWRKY27*, *OsWRKY73*, *OsWRKY64*, *OsWRKY25*, *WRKY117*, 107,277,882, *OsWRKY40*, *OsWRKY93*, and Novelgene_G000104 genes, belonging to the WRKY family, were only upregulated in the CKDN vs. D15DN group, and *OsWRKY29*, *OsWRKY83*, *OsWRKY27*, *OsWRKY73*, *OsWRKY25*, *OsWRKY61*, *OsWRKY93* and Novelgene_G000104 were involved in the MAPK signaling pathway. Furthermore, WRKY TFs have previously been found to be involved in Glu and Pro metabolism under abiotic stress (Kim et al. 2016; Mirabella et al. 2015; Dai et al. 2018). The transcription factor FcWRKY40 of *Fortunella crassifolia,* functions positively in Pro biosynthesis, by directly regulating *SOS2* and *P5CS1* homologs under salt stress (Dai et al. 2018). *OsWRKY40* may confer cold resistance in transgenic-plants by regulating Pro accumulation and a set of cold-stress responsive genes in the MAPK signaling pathway.

The CBF-COR regulatory signaling pathway is highly complex and requires further in-depth investigation (Ding et al. 2019). RNA-seq analysis of the triple mutants in previous investigations revealed that the expression of 10–20% of the COR genes were CBF-dependent (Jia et al. 2016; Zhao et al. 2016; Ding et al. 2019). In our study, CBF1 showed greater up regulation in D15DN than in D15SJ. ICE1 activates the expression of CBF genes by directly binding to their promoters under cold stress conditions. Mutations of ICE1 impairs cold-induced CBF expression and decreases freezing tolerance (Chinnusamy et al. 2003; Ding et al. 2015). In our experiment, ICE1(LOC_Os01g0928000) was up regulated to a greater degree in D15DN than in D15SJ.

Many WRKY proteins have been shown to respond to various abiotic stresses, such as cold (Zhang et al. 2012; Hwang et al. 2005). Several CBF-independent regulators, which are cold-induced transcription factors, function in a similar manner to CBFs, and can induce the expression of COR genes under cold stress, including WRKY33 and MYB73 (Park et al. 2015; Ding et al. 2019). In this study, we found that more WRKY and MYB genes were up regulated in CKDN vs. D15DN than in CKSJ vs. D15SJ.

### Nitrogen Metabolism Responses to Cold Stress

Although the actual role of Glu in plant osmotolerance remains controversial, there is supporting evidence for a positive effect on enzyme and membrane integrity, osmotic adjustment, and free radical scavenging. Still, some authors have argued that Glu accumulation under stress is a product of, and not an adaptive response to stress. A previous study found that the N uptake of rice was severely affected by low-T_w_ treatment during reproductive growth (Jia et al. 2019) and that Glu content and glutamate dehydrogenase (GDH) activity were important traits that influenced grain yield and spikelet sterility, respectively (Jia et al. 2015). In our study, we found that in the low-T_w_ treatment, the Glu content in D15DN was significantly reduced and the Pro content was slightly increased, compared with that of the control. In D15SJ, the trends in Glu and Pro content were opposite to those observed in D15DN. Compared with that of D15SJ, the Glu content in D15DN was 64.7% lower, while the Pro was 42.9% higher.

These results can be attributed to several factors. As indicated by the key enzyme activities of nitrogen metabolism and amino acid content (Fig. 1c, d), the increase in GOGAT activity and GS activity in D15SJ (69.0% and 59.3%) was greater than that of D15DN (43.8% and 29.6%); the increase in GDH activity in D15SJ compared with that of the control (58.0%), was lower than that observed in D15DN (85.3%). GDH activity has been demonstrated to confer resistance to various stress conditions (Dubois et al. 2003; Robredo et al. 2011) and GDH catalyzes the reversible oxidative deamination of Glu to supply 2-oxoglutarate and ammonium (Aubert et al. 2001). Secondly, Glu plays a central role in the amino acid metabolism of plants (Seifi et al. 2013), which may orchestrate crucial metabolic functions under abiotic stresses. For example, GABA and Pro biosynthesis each perform key roles in plant defense mechanisms (Forde and Lea 2007). A large amount of the Glu in D15DN was used in the biosynthesis of Pro, which led to a lower Glu content in D15DN, while Pro levels were higher.

Moreover, compared with D15SJ, the 1-ornithine carbamoyl transferase gene involved in L-ornithine synthesis showed significant upregulation in D15DN, indicating that low-T_w_ treatments may have promoted Glu metabolism, synthesis of ornithine, and Pro in D15DN.

## Conclusions

Cold stress during reproductive growth resulted in the genes and proteins related to the biosynthesis pathways of cold stress being significantly differentially expressed, in both rice cultivars investigated (DN and SJ). DN was more efficient in terms of N metabolism, the MAPK signaling pathway, and the regulation of transcription factors. The present study confirmed known cold stress-associated genes and identified new putative cold-response genes. The study revealed that translational regulation under cold stress plays an important role in cold-tolerant DN. The low-T_w_ treatment affected N uptake and N metabolism in rice, and promoted Glu metabolism, synthesis of ornithine, and Pro in cold-sensitive SJ.

## Materials and Methods

### Plant Material and Growth Conditions

Two *japonica* rice (*Oryza sativa*. *L. subsp*. *japonica*) cultivars, Dongnong428 (DN) and Songjing10 (SJ), which are currently used for local rice production in China, were utilized in this investigation. DN is a cold-resistant cultivar, while SJ is a cold-sensitive cultivar.

A split-plot design was used in this experiment. The whole plot factor had two levels, normal irrigation (CK) and 15 days of low-T_w_ irrigation (D15) during the reproductive growth stage of the rice. The cultivar of the rice (DN and SJ) was the subplot factor, the area of each subplot was 9 m^2^, and there were three replicates of the design. Approximately 30- to 35-day-old seedlings were transplanted with one seeding per hill at a spacing of 13.3 cm (hill space) by 30.0 cm (row space). The low-T_w_ treatment was started at the beginning of the reproductive growth stage, when the young panicle was approximately 1 cm in length (da Cruz et al. 2006). The low water temperature for the treatment was 17 °C, with an approximately 20 cm water layer depth; 17 °C is close to the temperature that causes cold damage to rice during reproductive growth in Heilongjiang Province, China (Jia et al. 2015). The cold water was generated automatically using a temperature controlled cold water irrigation system. Low-T_w_ irrigation was applied from 6:00 AM to 8:00 PM every day for the 15-day treatment period.

Gross root growth was measured using the previously reported mesh bag method (Steen 1991). For each root sample, according to the planned area of each hill, an iron tube (diameter 10 cm, length 30 cm) was inserted into the ground before transplanting. The soil in the tube was dug up with a spade. A mesh bag (diameter 10 cm, length 30 cm, mesh size 3 mm) was pulled onto the iron tube and then filled with soil. At the end of the stress period, the mesh bags were pulled out of the soil, and the roots were carefully rinsed and separated from their nodal bases. The temperature of the water used for washing is consistent with the water temperature of each treatment. Ten grams of roots from three samples were immediately frozen in liquid nitrogen for 10 min, and then stored at − 80 °C for RNA isolation and the N metabolism enzyme activity measurements. Ten grams of roots from another three samples were used for the dry matter measurements.

### Biomass and Nitrogen Metabolism Related Indicators

After being fixed at 105 °C for 30 mins, the samples were oven dried at 80 °C to a constant weight, and the dry weights of the roots were measured. An amino acid analyzer (HITACHI L-8900, Japan) was used for analysis of the amino acids. The GS activity was measured based on the method described by O'neal and Joy (1973). The GOGAT activity was determined based on the methods of Singh and Srivastava (1986). The GDH activity was determined according to Loulakakis and Roubelakis-Angelakis (1990).

### RNA Extraction, Illumina Transcriptome Library Preparation, and Sequencing

Total RNA was extracted from the rice shoots using a *TranZol* Up RNA kit (TransGen Biotech, Beijing, China). All samples were treated with DNase I (TransGen Biotech, Beijing, China). RNA quality was evaluated by gel electrophoresis on 1% agarose gels. RNA samples were further quantified and analyzed using a Nano Drop and Agilent 2100 bioanalyzer (Thermo Fisher Scientific, MA, USA). Then, 1 μg of total RNA from each sample was used to construct the cDNA library using the NEBNextUltra™ RNA Library Prep Kit for Illumina (NEB), following the manufacturer’s protocols, and index codes were added to attribute sequences to each sample. The mRNA was purified by Oligo (dT)-attached magnetic beads from the total RNA. Purified mRNA was fragmented into small pieces with fragment buffer at 20 °C. The RNA fragments were primed with random hexamers and reverse transcribed into the first strand of cDNA, and then the second strand was synthesized. The cDNA was purified by AMPure XP Beads and an A-Tailing Mix, and RNA Index Adapters were then added by incubating to end the repair. The cDNA fragments with adapters were amplified by PCR, and the products were purified by AMPure XP Beads, then dissolved in EB solution. The quality and quantity of the cDNA library was assessed using an Agilent 2100 bioanalyzer and real-time quantitative PCR. The qualified library was amplified on cBot (Meyer and Kircher 2010) to generate the clusters on the flow cell. After cluster generation, the libraries were sequenced on an Illumina Novaseq 6000 platform, according to the manufacturer’s instructions, and paired-end reads were obtained.

### Transcriptome Data Analysis

The raw reads were filtered to obtain clean reads, by removing those without adapter sequences and the low-quality reads (those containing over 10% N, or when the quality score of over 50% of the base was lower than 10); the clean reads were used for downstream analysis. The clean reads were aligned to the rice genome using TopHat (v2.0.9). The gene expression levels were calculated using the FPKM (fragments per kilobase of transcript per million mapped reads) and the Python package HTSeq with default settings. The DESeq R (1.10.1) package was used to evaluate the differential gene expression between the two groups. The resulting *P* values were adjusted using the Benjamini and Hochberg’s approach for controlling the false discovery rate (Anders and Huber, 2012). The differential expression of the genes was determined using the criteria of a false discovery rate (FDR) ≤0.01 and a fold change (FC) ≥2. The Plant Transcriptional Regulatory Map database was used to identify TFs and targeted gene relationships. Cytoscape was used to construct a network between the TFs and targeted genes (Tian et al. 2020; Shannon et al. 2003).

### Protein Preparation

Total protein was extracted from tissue using a Plant Total Protein Extraction Kit (Sigma-Aldrich, St. Louis, MO, USA), in accordance with the manufacturer’s instructions. The samples from the tissues were frozen in liquid nitrogen, homogenized to powders with 10% polyvinylpolypyrrolidone, and then homogenized in lysis buffer (8 M urea and 40 mM Tris-HCl, 2 mM EDTA, and 10 mM DTT, pH 8.5) containing 1 mM PMSF. Homogenates were sonicated for 5 min on ice. Samples were then centrifuged at 15,000×*g* for 20 min. The supernatant was transferred to a new tube and 5 volumes of 10% TCA/cold acetone was added to 10 mM DTT. The tubes were then kept at − 20 °C for 2 h or overnight to precipitate the protein. Next, tubes were centrifuged at 15,000×*g* for 20 min, and the supernatants discarded. Then 1 ml of cold acetone and 10 mM DTT were added to the tubes to suspend the sediment. After 30 min on ice, the samples were centrifuged at 25,000×*g* for 20 min, and the supernatant was discarded. This step was repeated until the supernatant was colorless.

The proteins were air dried and resuspended in lysis buffer. The samples were sonicated on ice for 5 min (2 s/3 s) to improve the dissolving of the protein. After centrifugation, the supernatants were incubated at 50 °C for 1 h for reduction and alkylated using 55 mM iodoacetamide (IAM) in the dark at room temperature for 45 min. Five volumes of cold acetone were added to the samples to precipitate the proteins at − 20 °C for 2 h or overnight. Lysis buffer was added to dissolve the proteins using sonication on ice for 5 min (2 s/3 s).

### iTRAQ Labeling and Peptide Fractionation

The peptides were dissolved in 30 μl 0.5 M TEAB. Peptide labelling was performed using the iTRAQ Reagent 8-plex Kit, according to the manufacturer’s instructions. The labelled peptides with different reagents were pooled and desalted with a Strata X C18 column (Phenomenex, Torrance, CA, USA), and dried by vacuum centrifugation, according to the manufacturer’s protocol.

The peptides were separated on a Shimadzu LC-20AB HPLC Pump system (Shimadzu Scientific Instruments, Kyoto, Japan) with a high pH RP column. The peptides were resuspended with buffer A (5% ACN, 95% H_2_O, pH 9.8) to 2 mL and loaded onto a column containing 5 μm particles (Phenomenex). The peptides were separated at a flow rate of 1 mL/min with a gradient of 5% buffer B (5% H_2_O, 95% ACN, pH 9.8) for 10 min, 5–35% buffer B for 40 min, and 35–95% buffer B for 1 min. Then, the system was maintained in 95% buffer B for 3 min and changed to 5% within 1 min, and then equilibrated with a 5% buffer B for 10 min. By measuring the elution absorbance at 214 nm, fractions were collected every 1 min. The eluted peptides were pooled into 20 fractions and then vacuum dried.

### LC-MS/MS Analyses Using the Q Exactive

The dried eluted peptide fraction was resuspended in buffer A (2% ACN and 0.1% FA) and centrifuged at 20,000×*g* for 10 min. The supernatant was loaded onto a C18 trap column 5 μL/min for 8 min, using an LC-20 AD nano-HPLC instrument (Shimadzu). After that, the peptides were eluted from the trap column and separated by an analytical C18 column (inner diameter 75 μm) packed in-house. The gradient was run at 300 nL/min from 8 to 35% of buffer B (2% H_2_O and 0.1% FA in ACN) for 35 min, then changed up to 60% for 5 min, then maintained at 80% buffer B for 5 min, and then decreased to 5% over 1 min, and equilibrated for 10 min.

The peptides separated from the nano HPLC were subjected to the tandem mass spectrometry Q Exactive (Thermo Fisher Scientific, San Jose, CA, USA) for data-dependent acquisition (DDA) detection, by nano-electrospray ionization. The parameters for MS analysis were as follows: electrospray voltage was 1.6 kV; precursor scan ranged from 350 to 1600 m/z at a resolution of 70,000; MS/MS fragment scan range was > 100 m/z at a resolution of 17,500 in HCD mode; normalized collision energy was 27%; dynamic exclusion time was 15 s; automatic gain control (AGC) for the full MS target and the MS2 target was respectively 3e6 and1e5; the number of MS/MS scans following one MS scan: the 20 most abundant precursor ions were above a threshold ion count of 20,000.

### Database Search and Quantification

Protein identification and quantification were performed with Proteome Discoverer Software (Thermo Fisher Scientific), using two included algorithms, Mascot and SEQUEST. Searches were constructed against the genome database. Peptide and MS/MS tolerance was set to 10 ppm and 0.6 Da, respectively. The enzyme specificity was set for trypsin, with two missed cleavages. Each confident protein identification and quantification required at least one unique peptide. The false discovery rate (FDR) of the identified proteins was ≤0.01. For each of the three biological repeats, spectra were combined into one file and searched. A peptide confidence level of 95%, or an unused confidence score > 1.3, were used as the qualification criteria. The relative quantification of the proteins was calculated based on the ratio of the peak areas from the MS/MS spectra. Differentially abundant proteins were determined using a Student’s t-test. Proteins with a < 0.05 Q-value and a fold change ≥1.2 were considered upregulated, and a fold change ≤0.83 was considered to be downregulated.

### Proteome Bioinformatic Analysis

The R package top GO version 2.28.0 was used for the GO enrichment analysis, with the classic algorithm option (each GO term was tested independently). The pathway enrichment analysis of the identified proteins was performed using the KOBAS software (KEGG ortholog-based annotation system, http://kobas.cbi.pku.edu.cn), and the analysis was conducted using a hypergeometric statistics test. The Benjamin-Hochberg correction was used to correct the probability values, and only the corrected P values that were ≤ 0.05 were considered significantly enriched pathways.

## Supplementary information

**Additional file 1: Table S1.** Summary of the RNA-Seq paired-end data produced by Illumina sequencing. **Table S2.** Significant KEGG pathways of DEGs (*P*-value ≤0.05) involved in DN and SJ under low-T_w_ treatment. **Table S3.** Significant KEGG pathways of the DEPs (*P*-value ≤0.05) involved in DN and SJ under low-T_w_ treatment. **Table S4.** A list of significantly enriched GO terms (*P*-value ≤0.05) with DEGs in DN and SJ under low-T_w_ treatment. **Table S5.** A list of significantly enriched GO terms (*P*-value ≤0.05) with DEPs in DN and SJ under low-T_w_ treatment. **Table S6.** A detailed list of the TFs expressed differentially in CKDN vs. D15DN under low-T_w_ treatment. **Table S7.** A detailed list of the TFs expressed differentially in CKSJ vs. D15SJ under low-T_w_ treatment.

**Additional file 2: Table S8.** Q-value of proteins in each group.

**Additional file 3: Figure S1.** Spearman correlations for the proteins and corresponding genes in each group.

**Additional file 4: Figure S2.** Targeted gene analysis of the TFs.

## Data Availability

The raw transcriptomic datasets from this study have been submitted to the NCBI Sequence Read Archive (SRA) under the accession number PRJNA557063. The query link is as follows: https://www.ncbi.nlm.nih.gov/sra/?term=PRJNA557063. The proteomic datasets have been submitted to iProX; raw data expected to be public on January 1, 2021. The mass spectrometry proteomics data have been deposited to the ProteomeXchange Consortium (http://proteomecentral.proteomexchange.org) via the iProX partner repository (Ma et al. 2019) with the dataset identifier **PXD018086.** After the data have been published, the access connection in ProteomeXchange will be: http://proteomecentral.proteomexchange.org/cgi/GetDataset? ID = PXD018086. After the data have been published, the access link in iProX will be: https://www.iprox.org/page/project.html?id=IPX0002064000

## Abbreviations

iTRAQ: Isobaric tags for relative and absolute quantification
DEP: Differentially expressed protein
DEG: Differentially expressed gene
KOBAS: KEGG Orthology-Based Annotation System
GO: Gene ontology
KEGG: Kyoto Encyclopedia of Genes and Genomes
