# Supplementary material for: Transcriptome Sequencing and iTRAQ of Different Rice Cultivars Provide Insight into Molecular Mechanisms of Cold-Tolerance Response in Japonica Rice
Source: Rice (N Y). 2020 Jun 22;13:43. doi: 10.1186/s12284-020-00401-8 (PMC7310054; doi:10.1186/s12284-020-00401-8)
Supplement: Supplementary file 1 — Additional file 1: Table S1. Summary of the RNA-Seq paired-end data produced by Illumina sequencing. Table S2. Significant KEGG pathways of DEGs (P-value ≤0.05) involved in DN and SJ under low-Tw treatment. Table S3. Significant KEGG pathways of the DEPs (P-value ≤0.05) involved in DN and SJ under low-Tw treatment. Table S4. A list of significantly enriched GO terms (P-value ≤0.05) with DEGs in DN and SJ under low-Tw treatment. Table S5. A list of significantly enriched GO terms (P-value ≤0.05) with DEPs in DN and SJ under low-Tw treatment. Table S6. A detailed list of the TFs expressed differentially in CKDN vs. D15DN under low-Tw treatment. Table S7. A detailed list of the TFs expressed differentially in CKSJ vs. D15SJ under low-Tw treatment. [file 12284_2020_401_MOESM1_ESM.docx]

**TableS1: Summary of RNA-Seq paired-end data produced by Illumina sequencing.**

| Sample | Total Raw Reads(Mb) | Total Clean Reads(Mb) | Total Clean Bases(Gb) | Clean Reads Q30(%) | Total MappingRatio | Uniquely MappingRatio |
| --- | --- | --- | --- | --- | --- | --- |
| CKDN_1 | 38.96 | 38.96 | 5.84 | 94.33 | 0.7581 | 0.6625 |
| CKDN_2 | 46.94 | 46.94 | 7.04 | 94.44 | 0.7619 | 0.6662 |
| CKDN_3 | 37.22 | 37.22 | 5.58 | 92.04 | 0.7654 | 0.6399 |
| CKSJ_1 | 36.55 | 36.55 | 5.48 | 92.81 | 0.851 | 0.7268 |
| CKSJ_3 | 37.44 | 37.44 | 5.62 | 94.44 | 0.8269 | 0.7221 |
| D15DN_1 | 30.85 | 30.85 | 4.63 | 94.17 | 0.7328 | 0.6421 |
| D15DN_2 | 40.15 | 40.14 | 6.02 | 94.07 | 0.8417 | 0.7385 |
| D15DN_3 | 31.93 | 31.93 | 4.79 | 94.1 | 0.7867 | 0.6868 |
| D15SJ_1 | 31.4 | 31.4 | 4.71 | 92.83 | 0.8802 | 0.7511 |
| D15SJ_2 | 33.03 | 33.02 | 4.95 | 94.08 | 0.8042 | 0.7098 |
| D15SJ_3 | 35.28 | 35.27 | 5.29 | 86.47 | 0.7741 | 0.5688 |

**Table S2.** Significant KEGG pathways of DEGs (P-value ≤0.05) involved in DN and SJ under low -T_w_ treatment.

| Group | Pathway | DEGs genes with pathway annotation (4011) | All genes with pathway annotation (22460) | Corrected P-value | Pathway ID | Level 1 | Level 2 |
| --- | --- | --- | --- | --- | --- | --- | --- |
| CKDN-VS-D15DN | MAPK signaling pathway - plant | 184 (4.59%) | 733 (3.26%) | 4.94E-05 | ko04016 | Environmental Information Processing | Signal transduction |
| CKDN-VS-D15DN | Diterpenoid biosynthesis | 39 (0.97%) | 108 (0.48%) | 3.08E-04 | ko00904 | Metabolism | Metabolism of terpenoids and polyketides |
| CKDN-VS-D15DN | Plant-pathogen interaction | 263 (6.56%) | 1153 (5.13%) | 3.08E-04 | ko04626 | Organismal Systems | Environmental adaptation |
| CKDN-VS-D15DN | Plant hormone signal transduction | 191 (4.76%) | 819 (3.65%) | 1.10E-03 | ko04075 | Environmental Information Processing | Signal transduction |
| CKDN-VS-D15DN | Limonene and pinene degradation | 19 (0.47%) | 49 (0.22%) | 1.17E-02 | ko00903 | Metabolism | Metabolism of terpenoids and polyketides |
| CKDN-VS-D15DN | Zeatin biosynthesis | 17 (0.42%) | 45 (0.2%) | 2.71E-02 | ko00908 | Metabolism | Metabolism of terpenoids and polyketides |
| CKSJ-VS-D15SJ | Plant-pathogen interaction | 276 (6.2%) | 1153 (5.13%) | 0.030867 | ko04626 | Organismal Systems | Environmental adaptation |

**Table S3.** Significant KEGG pathways of DEPs (P-value ≤0.05) involved in DN and SJ under low -T_w_ treatment.

| Group | Pathway | Diff Proteins with pathway annotation | All Proteins with pathway annotation | Corrected P-value | Pathway ID |
| --- | --- | --- | --- | --- | --- |
| CKDN-VS-D15DN | Biosynthesis of secondary metabolites | 72 (36.73%) | 1053 (17.24%) | 2.48E-11 | ko01110 |
| CKDN-VS-D15DN | Metabolic pathways | 89 (45.41%) | 1733 (28.38%) | 1.87E-07 | ko01100 |
| CKDN-VS-D15DN | Diterpenoid biosynthesis | 9 (4.59%) | 39 (0.64%) | 2.77E-06 | ko00904 |
| CKDN-VS-D15DN | Phenylpropanoid biosynthesis | 17 (8.67%) | 196 (3.21%) | 0.000165 | ko00940 |
| CKDN-VS-D15DN | Flavonoid biosynthesis | 8 (4.08%) | 53 (0.87%) | 0.00025 | ko00941 |
| CKDN-VS-D15DN | Cutin, suberine and wax biosynthesis | 4 (2.04%) | 17 (0.28%) | 0.001763 | ko00073 |
| CKSJ-VS-D15SJ | Metabolic pathways | 302 (42.72%) | 1733 (28.38%) | 2.46E-18 | ko01100 |
| CKSJ-VS-D15SJ | Biosynthesis of secondary metabolites | 201 (28.43%) | 1053 (17.24%) | 1.98E-15 | ko01110 |
| CKSJ-VS-D15SJ | Phenylpropanoid biosynthesis | 51 (7.21%) | 196 (3.21%) | 1.03E-08 | ko00940 |
| CKSJ-VS-D15SJ | Amino sugar and nucleotide sugar metabolism | 36 (5.09%) | 143 (2.34%) | 3.69E-06 | ko00520 |
| CKSJ-VS-D15SJ | Glutathione metabolism | 26 (3.68%) | 95 (1.56%) | 1.72E-05 | ko00480 |
| CKSJ-VS-D15SJ | Cysteine and methionine metabolism | 21 (2.97%) | 81 (1.33%) | 0.000253 | ko00270 |
| CKSJ-VS-D15SJ | Biosynthesis of amino acids | 46 (6.51%) | 242 (3.96%) | 0.00039 | ko01230 |
| CKSJ-VS-D15SJ | Carbon fixation in photosynthetic organisms | 20 (2.83%) | 83 (1.36%) | 0.000974 | ko00710 |
| CKSJ-VS-D15SJ | Carbon metabolism | 47 (6.65%) | 262 (4.29%) | 0.001265 | ko01200 |
| CKSJ-VS-D15SJ | Fatty acid biosynthesis | 10 (1.41%) | 31 (0.51%) | 0.001851 | ko00061 |
| CKSJ-VS-D15SJ | Flavonoid biosynthesis | 14 (1.98%) | 53 (0.87%) | 0.002183 | ko00941 |
| CKSJ-VS-D15SJ | Glyoxylate and dicarboxylate metabolism | 16 (2.26%) | 67 (1.1%) | 0.003355 | ko00630 |
| CKSJ-VS-D15SJ | Phenylalanine, tyrosine and tryptophan biosynthesis | 11 (1.56%) | 39 (0.64%) | 0.00367 | ko00400 |
| CKSJ-VS-D15SJ | Fatty acid metabolism | 14 (1.98%) | 57 (0.93%) | 0.004509 | ko01212 |

**Table S4.** A list of significantly enriched GO terms (P-value ≤0.05) having DEGs in DN and SJ under low -T_w_ treatment.

| Group | Gene Ontology term | Cluster frequency | Genome frequency of use | Corrected P-value |
| --- | --- | --- | --- | --- |
| CKDN-VS-D15DN | intrinsic component of membrane | 365 out of 2445 genes, 14.9% | 1709 out of 14018 genes, 12.2% | 0.00083 |
| CKDN-VS-D15DN | cell periphery | 83 out of 2445 genes, 3.4% | 300 out of 14018 genes, 2.1% | 0.001 |
| CKDN-VS-D15DN | plasma membrane | 60 out of 2445 genes, 2.5% | 203 out of 14018 genes, 1.4% | 0.00224 |
| CKDN-VS-D15DN | membrane | 470 out of 2445 genes, 19.2% | 2294 out of 14018 genes, 16.4% | 0.00339 |
| CKDN-VS-D15DN | integral component of membrane | 232 out of 2445 genes, 9.5% | 1068 out of 14018 genes, 7.6% | 0.01823 |
| CKDN-VS-D15DN | membrane part | 383 out of 2445 genes, 15.7% | 1871 out of 14018 genes, 13.3% | 0.02545 |
| CKDN-VS-D15DN | protein kinase activity | 228 out of 1979 genes, 11.5% | 936 out of 10769 genes, 8.7% | 0.00041 |
| CKDN-VS-D15DN | kinase activity | 276 out of 1979 genes, 13.9% | 1181 out of 10769 genes, 11.0% | 0.00114 |
| CKDN-VS-D15DN | phosphotransferase activity, alcohol group as acceptor | 238 out of 1979 genes, 12.0% | 1000 out of 10769 genes, 9.3% | 0.00151 |
| CKDN-VS-D15DN | transferase activity, transferring phosphorus-containing groups | 325 out of 1979 genes, 16.4% | 1470 out of 10769 genes, 13.7% | 0.02203 |
| CKDN-VS-D15DN | calmodulin binding | 13 out of 1979 genes, 0.7% | 24 out of 10769 genes, 0.2% | 0.03437 |
| CKDN-VS-D15DN | transferase activity | 562 out of 1979 genes, 28.4% | 2703 out of 10769 genes, 25.1% | 0.0463 |
| CKDN-VS-D15DN | diterpenoid metabolic process | 17 out of 1592 genes, 1.1% | 32 out of 8798 genes, 0.4% | 0.00693 |
| CKDN-VS-D15DN | cellular ion homeostasis | 12 out of 1592 genes, 0.8% | 19 out of 8798 genes, 0.2% | 0.01487 |
| CKDN-VS-D15DN | cellular cation homeostasis | 12 out of 1592 genes, 0.8% | 19 out of 8798 genes, 0.2% | 0.01487 |
| CKDN-VS-D15DN | terpenoid metabolic process | 25 out of 1592 genes, 1.6% | 61 out of 8798 genes, 0.7% | 0.0213 |
| CKDN-VS-D15DN | ion homeostasis | 14 out of 1592 genes, 0.9% | 26 out of 8798 genes, 0.3% | 0.03654 |
| CKDN-VS-D15DN | cellular metal ion homeostasis | 10 out of 1592 genes, 0.6% | 15 out of 8798 genes, 0.2% | 0.03984 |
| CKDN-VS-D15DN | metal ion transport | 45 out of 1592 genes, 2.8% | 141 out of 8798 genes, 1.6% | 0.04087 |
| CKSJ-VS-D15SJ | intrinsic component of membrane | 438 out of 2714 genes, 16.1% | 1709 out of 14018 genes, 12.2% | 1.32E-09 |
| CKSJ-VS-D15SJ | membrane | 555 out of 2714 genes, 20.4% | 2294 out of 14018 genes, 16.4% | 4.25E-08 |
| CKSJ-VS-D15SJ | membrane part | 457 out of 2714 genes, 16.8% | 1871 out of 14018 genes, 13.3% | 7.02E-07 |
| CKSJ-VS-D15SJ | integral component of membrane | 279 out of 2714 genes, 10.3% | 1068 out of 14018 genes, 7.6% | 2.22E-06 |
| CKSJ-VS-D15SJ | protein kinase activity | 254 out of 2280 genes, 11.1% | 936 out of 10769 genes, 8.7% | 0.00142 |
| CKSJ-VS-D15SJ | phosphotransferase activity, alcohol group as acceptor | 269 out of 2280 genes, 11.8% | 1000 out of 10769 genes, 9.3% | 0.0015 |
| CKSJ-VS-D15SJ | kinase activity | 311 out of 2280 genes, 13.6% | 1181 out of 10769 genes, 11.0% | 0.00179 |
| CKSJ-VS-D15SJ | transferase activity | 652 out of 2280 genes, 28.6% | 2703 out of 10769 genes, 25.1% | 0.0044 |
| CKSJ-VS-D15SJ | dioxygenase activity | 24 out of 2280 genes, 1.1% | 49 out of 10769 genes, 0.5% | 0.00624 |
| CKSJ-VS-D15SJ | ion binding | 745 out of 2280 genes, 32.7% | 3172 out of 10769 genes, 29.5% | 0.03965 |
| CKSJ-VS-D15SJ | oxidoreductase activity, acting on single donors with incorporation of molecular oxygen, incorporation of two atoms of oxygen | 15 out of 2280 genes, 0.7% | 27 out of 10769 genes, 0.3% | 0.04173 |
| CKSJ-VS-D15SJ | trehalose metabolic process | 12 out of 1830 genes, 0.7% | 17 out of 8798 genes, 0.2% | 0.01343 |
| CKSJ-VS-D15SJ | ion transport | 111 out of 1830 genes, 6.1% | 371 out of 8798 genes, 4.2% | 0.01404 |
| CKSJ-VS-D15SJ | metal ion transport | 50 out of 1830 genes, 2.7% | 141 out of 8798 genes, 1.6% | 0.03493 |
| CKSJ-VS-D15SJ | cellular protein modification process | 315 out of 1830 genes, 17.2% | 1256 out of 8798 genes, 14.3% | 0.04204 |
| CKSJ-VS-D15SJ | protein modification process | 315 out of 1830 genes, 17.2% | 1256 out of 8798 genes, 14.3% | 0.04204 |

**Table S5.** A list of significantly enriched GO terms (P-value ≤0.05) having DEPs in DN and SJ under low -T_w_ treatment.

| Group | Gene Ontology term | Cluster frequency | Protein frequency of use | Corrected P-value |
| --- | --- | --- | --- | --- |
| CKDN-VS D15DN | cytoplasmic membrane-bounded vesicle | 68 out of 165 genes, 41.2% | 1282 out of 5035 genes, 25.5% | 4.94E-06 |
| CKDN-VS-D15DN | cytoplasmic vesicle | 68 out of 165 genes, 41.2% | 1282 out of 5035 genes, 25.5% | 4.94E-06 |
| CKDN-VS-D15DN | membrane-bounded vesicle | 68 out of 165 genes, 41.2% | 1284 out of 5035 genes, 25.5% | 5.24E-06 |
| CKDN-VS-D15DN | vesicle | 68 out of 165 genes, 41.2% | 1285 out of 5035 genes, 25.5% | 5.40E-06 |
| CKDN-VS-D15DN | extracellular region | 18 out of 165 genes, 10.9% | 234 out of 5035 genes, 4.6% | 0.00057 |
| CKDN-VS-D15DN | amyloplast | 3 out of 165 genes, 1.8% | 7 out of 5035 genes, 0.1% | 0.001098 |
| CKDN-VS-D15DN | cytoplasm | 153 out of 165 genes, 92.7% | 4271 out of 5035 genes, 84.8% | 0.001442 |
| CKDN-VS-D15DN | cell surface | 2 out of 165 genes, 1.2% | 4 out of 5035 genes, 0.1% | 0.006132 |
| CKDN-VS-D15DN | cytoplasmic part | 145 out of 165 genes, 87.9% | 4049 out of 5035 genes, 80.4% | 0.006894 |
| CKDN-VS-D15DN | apoplast | 13 out of 165 genes, 7.9% | 184 out of 5035 genes, 3.7% | 0.006935 |
| CKDN-VS-D15DN | phosphopyruvate hydratase complex | 2 out of 165 genes, 1.2% | 5 out of 5035 genes, 0.1% | 0.010001 |
| CKDN-VS-D15DN | intracellular part | 156 out of 165 genes, 94.5% | 4550 out of 5035 genes, 90.4% | 0.035975 |
| CKDN-VS-D15DN | anchored to membrane | 3 out of 165 genes, 1.8% | 23 out of 5035 genes, 0.5% | 0.037833 |
| CKDN-VS-D15DN | cell | 163 out of 165 genes, 98.8% | 4840 out of 5035 genes, 96.1% | 0.041383 |
| CKDN-VS-D15DN | cell part | 163 out of 165 genes, 98.8% | 4840 out of 5035 genes, 96.1% | 0.041383 |
| CKDN-VS-D15DN | thylakoid | 9 out of 165 genes, 5.5% | 143 out of 5035 genes, 2.8% | 0.044143 |
| CKDN-VS-D15DN | oxidoreductase activity | 48 out of 163 genes, 29.4% | 737 out of 4595 genes, 16.0% | 8.64E-06 |
| CKDN-VS-D15DN | heme binding | 16 out of 163 genes, 9.8% | 140 out of 4595 genes, 3.0% | 2.82E-05 |
| CKDN-VS-D15DN | tetrapyrrole binding | 16 out of 163 genes, 9.8% | 146 out of 4595 genes, 3.2% | 4.75E-05 |
| CKDN-VS-D15DN | cation binding | 60 out of 163 genes, 36.8% | 1074 out of 4595 genes, 23.4% | 5.90E-05 |
| CKDN-VS-D15DN | catalytic activity | 137 out of 163 genes, 84.0% | 3324 out of 4595 genes, 72.3% | 0.000255 |
| CKDN-VS-D15DN | iron ion binding | 11 out of 163 genes, 6.7% | 96 out of 4595 genes, 2.1% | 0.000516 |
| CKDN-VS-D15DN | metal ion binding | 53 out of 163 genes, 32.5% | 993 out of 4595 genes, 21.6% | 0.000668 |
| CKDN-VS-D15DN | nutrient reservoir activity | 5 out of 163 genes, 3.1% | 21 out of 4595 genes, 0.5% | 0.000678 |
| CKDN-VS-D15DN | antioxidant activity | 12 out of 163 genes, 7.4% | 121 out of 4595 genes, 2.6% | 0.001079 |
| CKDN-VS-D15DN | ent-copalyl diphosphate synthase activity | 2 out of 163 genes, 1.2% | 2 out of 4595 genes, 0.0% | 0.001251 |
| CKDN-VS-D15DN | glutamate synthase (NADH) activity | 2 out of 163 genes, 1.2% | 2 out of 4595 genes, 0.0% | 0.001251 |
| CKDN-VS-D15DN | glutamate synthase activity, NAD(P)H as acceptor | 2 out of 163 genes, 1.2% | 2 out of 4595 genes, 0.0% | 0.001251 |
| CKDN-VS-D15DN | 4-hydroxy-3-methylbut-2-en-1-yl diphosphate synthase activity | 2 out of 163 genes, 1.2% | 2 out of 4595 genes, 0.0% | 0.001251 |
| CKDN-VS-D15DN | syn-copalyl diphosphate synthase activity | 2 out of 163 genes, 1.2% | 2 out of 4595 genes, 0.0% | 0.001251 |
| CKDN-VS-D15DN | oxidoreductase activity, acting on CH or CH2 groups, with an iron-sulfur protein as acceptor | 2 out of 163 genes, 1.2% | 2 out of 4595 genes, 0.0% | 0.001251 |
| CKDN-VS-D15DN | intramolecular lyase activity | 3 out of 163 genes, 1.8% | 7 out of 4595 genes, 0.2% | 0.001381 |
| CKDN-VS-D15DN | peroxidase activity | 10 out of 163 genes, 6.1% | 102 out of 4595 genes, 2.2% | 0.003053 |
| CKDN-VS-D15DN | oxidoreductase activity, acting on peroxide as acceptor | 10 out of 163 genes, 6.1% | 102 out of 4595 genes, 2.2% | 0.003053 |
| CKDN-VS-D15DN | 1,4-alpha-glucan branching enzyme activity | 2 out of 163 genes, 1.2% | 3 out of 4595 genes, 0.1% | 0.003665 |
| CKDN-VS-D15DN | phosphoglycerate kinase activity | 2 out of 163 genes, 1.2% | 3 out of 4595 genes, 0.1% | 0.003665 |
| CKDN-VS-D15DN | glucose-1-phosphate adenylyltransferase activity | 2 out of 163 genes, 1.2% | 3 out of 4595 genes, 0.1% | 0.003665 |
| CKDN-VS-D15DN | glutamate synthase activity | 2 out of 163 genes, 1.2% | 3 out of 4595 genes, 0.1% | 0.003665 |
| CKDN-VS-D15DN | oxidation-reduction process | 51 out of 158 genes, 32.3% | 674 out of 4250 genes, 15.9% | 1.32E-07 |
| CKDN-VS-D15DN | single-organism metabolic process | 79 out of 158 genes, 50.0% | 1433 out of 4250 genes, 33.7% | 1.23E-05 |
| CKDN-VS-D15DN | diterpene phytoalexin metabolic process | 5 out of 158 genes, 3.2% | 12 out of 4250 genes, 0.3% | 4.27E-05 |
| CKDN-VS-D15DN | glycogen metabolic process | 4 out of 158 genes, 2.5% | 9 out of 4250 genes, 0.2% | 0.0002 |
| CKDN-VS-D15DN | glycogen biosynthetic process | 4 out of 158 genes, 2.5% | 9 out of 4250 genes, 0.2% | 0.0002 |
| CKDN-VS-D15DN | energy reserve metabolic process | 4 out of 158 genes, 2.5% | 9 out of 4250 genes, 0.2% | 0.0002 |
| CKDN-VS-D15DN | phytoalexin metabolic process | 5 out of 158 genes, 3.2% | 16 out of 4250 genes, 0.4% | 0.000209 |
| CKDN-VS-D15DN | terpenoid metabolic process | 8 out of 158 genes, 5.1% | 46 out of 4250 genes, 1.1% | 0.000239 |
| CKDN-VS-D15DN | terpenoid biosynthetic process | 7 out of 158 genes, 4.4% | 36 out of 4250 genes, 0.8% | 0.000289 |
| CKDN-VS-D15DN | generation of precursor metabolites and energy | 20 out of 158 genes, 12.7% | 234 out of 4250 genes, 5.5% | 0.000346 |
| CKDN-VS-D15DN | carbohydrate metabolic process | 30 out of 158 genes, 19.0% | 427 out of 4250 genes, 10.0% | 0.000375 |
| CKDN-VS-D15DN | diterpenoid metabolic process | 5 out of 158 genes, 3.2% | 18 out of 4250 genes, 0.4% | 0.000386 |
| CKDN-VS-D15DN | response to stress | 45 out of 158 genes, 28.5% | 766 out of 4250 genes, 18.0% | 0.000667 |
| CKDN-VS-D15DN | dicarboxylic acid biosynthetic process | 4 out of 158 genes, 2.5% | 12 out of 4250 genes, 0.3% | 0.000721 |
| CKDN-VS-D15DN | response to temperature stimulus | 16 out of 158 genes, 10.1% | 180 out of 4250 genes, 4.2% | 0.000915 |
| CKDN-VS-D15DN | D-xylose metabolic process | 3 out of 158 genes, 1.9% | 6 out of 4250 genes, 0.1% | 0.000928 |
| CKDN-VS-D15DN | diterpene phytoalexin biosynthetic process | 3 out of 158 genes, 1.9% | 6 out of 4250 genes, 0.1% | 0.000928 |
| CKDN-VS-D15DN | single-organism carbohydrate metabolic process | 20 out of 158 genes, 12.7% | 254 out of 4250 genes, 6.0% | 0.000999 |
| CKDN-VS-D15DN | isoprenoid metabolic process | 8 out of 158 genes, 5.1% | 59 out of 4250 genes, 1.4% | 0.001348 |
| CKDN-VS-D15DN | suberin biosynthetic process | 2 out of 158 genes, 1.3% | 2 out of 4250 genes, 0.0% | 0.001374 |
| CKDN-VS-D15DN | isoprenoid biosynthetic process | 7 out of 158 genes, 4.4% | 50 out of 4250 genes, 1.2% | 0.002232 |
| CKDN-VS-D15DN | dicarboxylic acid metabolic process | 7 out of 158 genes, 4.4% | 52 out of 4250 genes, 1.2% | 0.002809 |
| CKDN-VS-D15DN | lipid biosynthetic process | 12 out of 158 genes, 7.6% | 131 out of 4250 genes, 3.1% | 0.003143 |
| CKDN-VS-D15DN | cellular ion homeostasis | 4 out of 158 genes, 2.5% | 18 out of 4250 genes, 0.4% | 0.003742 |
| CKDN-VS-D15DN | monosaccharide metabolic process | 12 out of 158 genes, 7.6% | 134 out of 4250 genes, 3.2% | 0.003791 |
| CKDN-VS-D15DN | glutamate biosynthetic process | 2 out of 158 genes, 1.3% | 3 out of 4250 genes, 0.1% | 0.00402 |
| CKDN-VS-D15DN | response to cold | 11 out of 158 genes, 7.0% | 119 out of 4250 genes, 2.8% | 0.004333 |
| CKDN-VS-D15DN | response to oxidative stress | 14 out of 158 genes, 8.9% | 173 out of 4250 genes, 4.1% | 0.004579 |
| CKDN-VS-D15DN | diterpenoid biosynthetic process | 3 out of 158 genes, 1.9% | 10 out of 4250 genes, 0.2% | 0.00499 |
| CKDN-VS-D15DN | phytoalexin biosynthetic process | 3 out of 158 genes, 1.9% | 10 out of 4250 genes, 0.2% | 0.00499 |
| CKSJ-VS-D15SJ | extracellular region | 62 out of 602 genes, 10.3% | 234 out of 5035 genes, 4.6% | 3.35E-10 |
| CKSJ-VS-D15SJ | cytoplasmic membrane-bounded vesicle | 216 out of 602 genes, 35.9% | 1282 out of 5035 genes, 25.5% | 9.00E-10 |
| CKSJ-VS-D15SJ | cytoplasmic vesicle | 216 out of 602 genes, 35.9% | 1282 out of 5035 genes, 25.5% | 9.00E-10 |
| CKSJ-VS-D15SJ | membrane-bounded vesicle | 216 out of 602 genes, 35.9% | 1284 out of 5035 genes, 25.5% | 1.05E-09 |
| CKSJ-VS-D15SJ | vesicle | 216 out of 602 genes, 35.9% | 1285 out of 5035 genes, 25.5% | 1.13E-09 |
| CKSJ-VS-D15SJ | apoplast | 51 out of 602 genes, 8.5% | 184 out of 5035 genes, 3.7% | 2.58E-09 |
| CKSJ-VS-D15SJ | cell periphery | 193 out of 602 genes, 32.1% | 1141 out of 5035 genes, 22.7% | 9.31E-09 |
| CKSJ-VS-D15SJ | cell wall | 71 out of 602 genes, 11.8% | 325 out of 5035 genes, 6.5% | 1.27E-07 |
| CKSJ-VS-D15SJ | external encapsulating structure | 71 out of 602 genes, 11.8% | 325 out of 5035 genes, 6.5% | 1.27E-07 |
| CKSJ-VS-D15SJ | plasma membrane | 152 out of 602 genes, 25.2% | 944 out of 5035 genes, 18.7% | 1.54E-05 |
| CKSJ-VS-D15SJ | vacuole | 71 out of 602 genes, 11.8% | 371 out of 5035 genes, 7.4% | 2.15E-05 |
| CKSJ-VS-D15SJ | cytoplasm | 543 out of 602 genes, 90.2% | 4271 out of 5035 genes, 84.8% | 2.73E-05 |
| CKSJ-VS-D15SJ | chloroplast stroma | 52 out of 602 genes, 8.6% | 266 out of 5035 genes, 5.3% | 0.000166 |
| CKSJ-VS-D15SJ | plastid stroma | 52 out of 602 genes, 8.6% | 271 out of 5035 genes, 5.4% | 0.00027 |
| CKSJ-VS-D15SJ | chloroplast part | 71 out of 602 genes, 11.8% | 430 out of 5035 genes, 8.5% | 0.002133 |
| CKSJ-VS-D15SJ | plastid part | 71 out of 602 genes, 11.8% | 436 out of 5035 genes, 8.7% | 0.003079 |
| CKSJ-VS-D15SJ | plant-type cell wall | 22 out of 602 genes, 3.7% | 105 out of 5035 genes, 2.1% | 0.00553 |
| CKSJ-VS-D15SJ | chloroplast thylakoid | 19 out of 602 genes, 3.2% | 90 out of 5035 genes, 1.8% | 0.008811 |
| CKSJ-VS-D15SJ | plastid thylakoid | 19 out of 602 genes, 3.2% | 90 out of 5035 genes, 1.8% | 0.008811 |
| CKSJ-VS-D15SJ | cell | 589 out of 602 genes, 97.8% | 4840 out of 5035 genes, 96.1% | 0.009645 |
| CKSJ-VS-D15SJ | cell part | 589 out of 602 genes, 97.8% | 4840 out of 5035 genes, 96.1% | 0.009645 |
| CKSJ-VS-D15SJ | thylakoid | 27 out of 602 genes, 4.5% | 143 out of 5035 genes, 2.8% | 0.009821 |
| CKSJ-VS-D15SJ | chloroplast | 133 out of 602 genes, 22.1% | 931 out of 5035 genes, 18.5% | 0.009826 |
| CKSJ-VS-D15SJ | organelle subcompartment | 19 out of 602 genes, 3.2% | 92 out of 5035 genes, 1.8% | 0.011167 |
| CKSJ-VS-D15SJ | plastoglobule | 7 out of 602 genes, 1.2% | 22 out of 5035 genes, 0.4% | 0.01146 |
| CKSJ-VS-D15SJ | chloroplast thylakoid membrane | 17 out of 602 genes, 2.8% | 82 out of 5035 genes, 1.6% | 0.015318 |
| CKSJ-VS-D15SJ | plastid thylakoid membrane | 17 out of 602 genes, 2.8% | 82 out of 5035 genes, 1.6% | 0.015318 |
| CKSJ-VS-D15SJ | thylakoid membrane | 17 out of 602 genes, 2.8% | 85 out of 5035 genes, 1.7% | 0.021497 |
| CKSJ-VS-D15SJ | thylakoid part | 18 out of 602 genes, 3.0% | 92 out of 5035 genes, 1.8% | 0.022623 |
| CKSJ-VS-D15SJ | storage vacuole | 3 out of 602 genes, 0.5% | 6 out of 5035 genes, 0.1% | 0.025757 |
| CKSJ-VS-D15SJ | protein storage vacuole | 3 out of 602 genes, 0.5% | 6 out of 5035 genes, 0.1% | 0.025757 |
| CKSJ-VS-D15SJ | eukaryotic translation elongation factor 1 complex | 3 out of 602 genes, 0.5% | 6 out of 5035 genes, 0.1% | 0.025757 |
| CKSJ-VS-D15SJ | photosynthetic membrane | 17 out of 602 genes, 2.8% | 87 out of 5035 genes, 1.7% | 0.026567 |
| CKSJ-VS-D15SJ | cytoplasmic part | 502 out of 602 genes, 83.4% | 4049 out of 5035 genes, 80.4% | 0.02696 |
| CKSJ-VS-D15SJ | chloroplast ribulose bisphosphate carboxylase complex | 2 out of 602 genes, 0.3% | 3 out of 5035 genes, 0.1% | 0.03942 |
| CKSJ-VS-D15SJ | central vacuole | 2 out of 602 genes, 0.3% | 3 out of 5035 genes, 0.1% | 0.03942 |
| CKSJ-VS-D15SJ | ribulose bisphosphate carboxylase complex | 2 out of 602 genes, 0.3% | 3 out of 5035 genes, 0.1% | 0.03942 |
| CKSJ-VS-D15SJ | stromule | 7 out of 602 genes, 1.2% | 28 out of 5035 genes, 0.6% | 0.042308 |
| CKSJ-VS-D15SJ | chloroplast envelope | 35 out of 602 genes, 5.8% | 221 out of 5035 genes, 4.4% | 0.047267 |
| CKSJ-VS-D15SJ | oxidoreductase activity | 146 out of 602 genes, 24.3% | 737 out of 4595 genes, 16.0% | 1.30E-08 |
| CKSJ-VS-D15SJ | tetrapyrrole binding | 43 out of 602 genes, 7.1% | 146 out of 4595 genes, 3.2% | 9.95E-08 |
| CKSJ-VS-D15SJ | nutrient reservoir activity | 13 out of 602 genes, 2.2% | 21 out of 4595 genes, 0.5% | 2.21E-07 |
| CKSJ-VS-D15SJ | heme binding | 41 out of 602 genes, 6.8% | 140 out of 4595 genes, 3.0% | 2.37E-07 |
| CKSJ-VS-D15SJ | peroxidase activity | 31 out of 602 genes, 5.1% | 102 out of 4595 genes, 2.2% | 3.07E-06 |
| CKSJ-VS-D15SJ | oxidoreductase activity, acting on peroxide as acceptor | 31 out of 602 genes, 5.1% | 102 out of 4595 genes, 2.2% | 3.07E-06 |
| CKSJ-VS-D15SJ | antioxidant activity | 34 out of 602 genes, 5.6% | 121 out of 4595 genes, 2.6% | 7.19E-06 |
| CKSJ-VS-D15SJ | catalytic activity | 473 out of 602 genes, 78.6% | 3324 out of 4595 genes, 72.3% | 0.000111 |
| CKSJ-VS-D15SJ | hydrolase activity, hydrolyzing O-glycosyl compounds | 36 out of 602 genes, 6.0% | 150 out of 4595 genes, 3.3% | 0.000158 |
| CKSJ-VS-D15SJ | manganese ion binding | 13 out of 602 genes, 2.2% | 34 out of 4595 genes, 0.7% | 0.000195 |
| CKSJ-VS-D15SJ | cation binding | 176 out of 602 genes, 29.2% | 1074 out of 4595 genes, 23.4% | 0.000213 |
| CKSJ-VS-D15SJ | hydrolase activity, acting on glycosyl bonds | 38 out of 602 genes, 6.3% | 164 out of 4595 genes, 3.6% | 0.000228 |
| CKSJ-VS-D15SJ | oxidoreductase activity, acting on CH-OH group of donors | 30 out of 602 genes, 5.0% | 124 out of 4595 genes, 2.7% | 0.000477 |
| CKSJ-VS-D15SJ | transferase activity, transferring alkyl or aryl (other than methyl) groups | 16 out of 602 genes, 2.7% | 53 out of 4595 genes, 1.2% | 0.000841 |
| CKSJ-VS-D15SJ | copper ion binding | 31 out of 602 genes, 5.1% | 136 out of 4595 genes, 3.0% | 0.001137 |
| CKSJ-VS-D15SJ | cofactor binding | 57 out of 602 genes, 9.5% | 299 out of 4595 genes, 6.5% | 0.001649 |
| CKSJ-VS-D15SJ | serine-type carboxypeptidase activity | 8 out of 602 genes, 1.3% | 19 out of 4595 genes, 0.4% | 0.001659 |
| CKSJ-VS-D15SJ | glycogenin glucosyltransferase activity | 3 out of 602 genes, 0.5% | 3 out of 4595 genes, 0.1% | 0.002239 |
| CKSJ-VS-D15SJ | iron ion binding | 23 out of 602 genes, 3.8% | 96 out of 4595 genes, 2.1% | 0.002439 |
| CKSJ-VS-D15SJ | adenylate kinase activity | 5 out of 602 genes, 0.8% | 9 out of 4595 genes, 0.2% | 0.003037 |
| CKSJ-VS-D15SJ | glutathione transferase activity | 9 out of 602 genes, 1.5% | 25 out of 4595 genes, 0.5% | 0.003107 |
| CKSJ-VS-D15SJ | oxidoreductase activity, acting on single donors with incorporation of molecular oxygen, incorporation of two atoms of oxygen | 8 out of 602 genes, 1.3% | 21 out of 4595 genes, 0.5% | 0.003515 |
| CKSJ-VS-D15SJ | carboxypeptidase activity | 10 out of 602 genes, 1.7% | 30 out of 4595 genes, 0.7% | 0.003544 |
| CKSJ-VS-D15SJ | oxidoreductase activity, acting on the aldehyde or oxo group of donors | 12 out of 602 genes, 2.0% | 40 out of 4595 genes, 0.9% | 0.003901 |
| CKSJ-VS-D15SJ | serine-type exopeptidase activity | 8 out of 602 genes, 1.3% | 22 out of 4595 genes, 0.5% | 0.004901 |
| CKSJ-VS-D15SJ | lyase activity | 32 out of 602 genes, 5.3% | 155 out of 4595 genes, 3.4% | 0.005082 |
| CKSJ-VS-D15SJ | metal ion binding | 155 out of 602 genes, 25.7% | 993 out of 4595 genes, 21.6% | 0.005325 |
| CKSJ-VS-D15SJ | transition metal ion binding | 90 out of 602 genes, 15.0% | 543 out of 4595 genes, 11.8% | 0.007586 |
| CKSJ-VS-D15SJ | phosphotransferase activity, phosphate group as acceptor | 7 out of 602 genes, 1.2% | 19 out of 4595 genes, 0.4% | 0.007761 |
| CKSJ-VS-D15SJ | serine-type peptidase activity | 18 out of 602 genes, 3.0% | 76 out of 4595 genes, 1.7% | 0.007886 |
| CKSJ-VS-D15SJ | serine hydrolase activity | 18 out of 602 genes, 3.0% | 76 out of 4595 genes, 1.7% | 0.007886 |
| CKSJ-VS-D15SJ | L-phenylalanine aminotransferase activity | 3 out of 602 genes, 0.5% | 4 out of 4595 genes, 0.1% | 0.00808 |
| CKSJ-VS-D15SJ | L-phenylalanine:2-oxoglutarate aminotransferase activity | 3 out of 602 genes, 0.5% | 4 out of 4595 genes, 0.1% | 0.00808 |
| CKSJ-VS-D15SJ | nucleobase-containing compound kinase activity | 8 out of 602 genes, 1.3% | 24 out of 4595 genes, 0.5% | 0.008883 |
| CKSJ-VS-D15SJ | beta-fructofuranosidase activity | 4 out of 602 genes, 0.7% | 8 out of 4595 genes, 0.2% | 0.013208 |
| CKSJ-VS-D15SJ | sucrose alpha-glucosidase activity | 4 out of 602 genes, 0.7% | 8 out of 4595 genes, 0.2% | 0.013208 |
| CKSJ-VS-D15SJ | oxidoreductase activity, acting on single donors with incorporation of molecular oxygen | 8 out of 602 genes, 1.3% | 26 out of 4595 genes, 0.6% | 0.014885 |
| CKSJ-VS-D15SJ | isomerase activity | 23 out of 602 genes, 3.8% | 111 out of 4595 genes, 2.4% | 0.0155 |
| CKSJ-VS-D15SJ | pyridoxal phosphate binding | 17 out of 602 genes, 2.8% | 76 out of 4595 genes, 1.7% | 0.017075 |
| CKSJ-VS-D15SJ | 5-methyltetrahydropteroyltriglutamate-homocysteine S-methyltransferase activity | 2 out of 602 genes, 0.3% | 2 out of 4595 genes, 0.0% | 0.017139 |
| CKSJ-VS-D15SJ | carbonate dehydratase activity | 2 out of 602 genes, 0.3% | 2 out of 4595 genes, 0.0% | 0.017139 |
| CKSJ-VS-D15SJ | cytidylate kinase activity | 2 out of 602 genes, 0.3% | 2 out of 4595 genes, 0.0% | 0.017139 |
| CKSJ-VS-D15SJ | ketol-acid reductoisomerase activity | 2 out of 602 genes, 0.3% | 2 out of 4595 genes, 0.0% | 0.017139 |
| CKSJ-VS-D15SJ | L-lactate dehydrogenase activity | 2 out of 602 genes, 0.3% | 2 out of 4595 genes, 0.0% | 0.017139 |
| CKSJ-VS-D15SJ | S-methyltransferase activity | 2 out of 602 genes, 0.3% | 2 out of 4595 genes, 0.0% | 0.017139 |
| CKSJ-VS-D15SJ | methionine synthase activity | 2 out of 602 genes, 0.3% | 2 out of 4595 genes, 0.0% | 0.017139 |
| CKSJ-VS-D15SJ | 5-methyltetrahydrofolate-dependent methyltransferase activity | 2 out of 602 genes, 0.3% | 2 out of 4595 genes, 0.0% | 0.017139 |
| CKSJ-VS-D15SJ | 5-methyltetrahydropteroyltri-L-glutamate-dependent methyltransferase activity | 2 out of 602 genes, 0.3% | 2 out of 4595 genes, 0.0% | 0.017139 |
| CKSJ-VS-D15SJ | 4-hydroxy-3-methylbut-2-en-1-yl diphosphate synthase activity | 2 out of 602 genes, 0.3% | 2 out of 4595 genes, 0.0% | 0.017139 |
| CKSJ-VS-D15SJ | oxidoreductase activity, acting on CH or CH2 groups, with an iron-sulfur protein as acceptor | 2 out of 602 genes, 0.3% | 2 out of 4595 genes, 0.0% | 0.017139 |
| CKSJ-VS-D15SJ | L-aspartate:2-oxoglutarate aminotransferase activity | 3 out of 602 genes, 0.5% | 5 out of 4595 genes, 0.1% | 0.018237 |
| CKSJ-VS-D15SJ | glyceraldehyde-3-phosphate dehydrogenase (NAD+) (phosphorylating) activity | 3 out of 602 genes, 0.5% | 5 out of 4595 genes, 0.1% | 0.018237 |
| CKSJ-VS-D15SJ | sucrose synthase activity | 3 out of 602 genes, 0.5% | 5 out of 4595 genes, 0.1% | 0.018237 |
| CKSJ-VS-D15SJ | carbon-oxygen lyase activity | 13 out of 602 genes, 2.2% | 54 out of 4595 genes, 1.2% | 0.019482 |
| CKSJ-VS-D15SJ | nucleotide kinase activity | 5 out of 602 genes, 0.8% | 13 out of 4595 genes, 0.3% | 0.019787 |
| CKSJ-VS-D15SJ | lipoxygenase activity | 4 out of 602 genes, 0.7% | 9 out of 4595 genes, 0.2% | 0.021346 |
| CKSJ-VS-D15SJ | carbon-oxygen lyase activity, acting on phosphates | 4 out of 602 genes, 0.7% | 9 out of 4595 genes, 0.2% | 0.021346 |
| CKSJ-VS-D15SJ | glucosidase activity | 7 out of 602 genes, 1.2% | 23 out of 4595 genes, 0.5% | 0.023673 |
| CKSJ-VS-D15SJ | carbon-nitrogen ligase activity, with glutamine as amido-N-donor | 5 out of 602 genes, 0.8% | 14 out of 4595 genes, 0.3% | 0.027539 |
| CKSJ-VS-D15SJ | ion binding | 275 out of 602 genes, 45.7% | 1931 out of 4595 genes, 42.0% | 0.028598 |
| CKSJ-VS-D15SJ | coenzyme binding | 36 out of 602 genes, 6.0% | 202 out of 4595 genes, 4.4% | 0.030614 |
| CKSJ-VS-D15SJ | alpha-mannosidase activity | 3 out of 602 genes, 0.5% | 6 out of 4595 genes, 0.1% | 0.032957 |
| CKSJ-VS-D15SJ | mannosidase activity | 3 out of 602 genes, 0.5% | 6 out of 4595 genes, 0.1% | 0.032957 |
| CKSJ-VS-D15SJ | phenylalanine ammonia-lyase activity | 3 out of 602 genes, 0.5% | 6 out of 4595 genes, 0.1% | 0.032957 |
| CKSJ-VS-D15SJ | exopeptidase activity | 12 out of 602 genes, 2.0% | 53 out of 4595 genes, 1.2% | 0.037948 |
| CKSJ-VS-D15SJ | 3-deoxy-7-phosphoheptulonate synthase activity | 2 out of 602 genes, 0.3% | 3 out of 4595 genes, 0.1% | 0.04694 |
| CKSJ-VS-D15SJ | UDP-glucose 6-dehydrogenase activity | 2 out of 602 genes, 0.3% | 3 out of 4595 genes, 0.1% | 0.04694 |
| CKSJ-VS-D15SJ | catalase activity | 2 out of 602 genes, 0.3% | 3 out of 4595 genes, 0.1% | 0.04694 |
| CKSJ-VS-D15SJ | lactate dehydrogenase activity | 2 out of 602 genes, 0.3% | 3 out of 4595 genes, 0.1% | 0.04694 |
| CKSJ-VS-D15SJ | alpha-galactosidase activity | 2 out of 602 genes, 0.3% | 3 out of 4595 genes, 0.1% | 0.04694 |
| CKSJ-VS-D15SJ | urea transmembrane transporter activity | 2 out of 602 genes, 0.3% | 3 out of 4595 genes, 0.1% | 0.04694 |
| CKSJ-VS-D15SJ | stemar-13-ene synthase activity | 2 out of 602 genes, 0.3% | 3 out of 4595 genes, 0.1% | 0.04694 |
| CKSJ-VS-D15SJ | ent-sandaracopimaradiene synthase activity | 2 out of 602 genes, 0.3% | 3 out of 4595 genes, 0.1% | 0.04694 |
| CKSJ-VS-D15SJ | ent-isokaurene synthase activity | 2 out of 602 genes, 0.3% | 3 out of 4595 genes, 0.1% | 0.04694 |
| CKSJ-VS-D15SJ | ent-pimara-8(14),15-diene synthase activity | 2 out of 602 genes, 0.3% | 3 out of 4595 genes, 0.1% | 0.04694 |
| CKSJ-VS-D15SJ | syn-stemod-13(17)-ene synthase activity | 2 out of 602 genes, 0.3% | 3 out of 4595 genes, 0.1% | 0.04694 |
| CKSJ-VS-D15SJ | single-organism metabolic process | 261 out of 549 genes, 47.5% | 1433 out of 4250 genes, 33.7% | 4.54E-13 |
| CKSJ-VS-D15SJ | response to stimulus | 238 out of 549 genes, 43.4% | 1310 out of 4250 genes, 30.8% | 2.06E-11 |
| CKSJ-VS-D15SJ | response to inorganic substance | 98 out of 549 genes, 17.9% | 402 out of 4250 genes, 9.5% | 2.72E-11 |
| CKSJ-VS-D15SJ | response to cadmium ion | 71 out of 549 genes, 12.9% | 276 out of 4250 genes, 6.5% | 1.91E-09 |
| CKSJ-VS-D15SJ | response to stress | 151 out of 549 genes, 27.5% | 766 out of 4250 genes, 18.0% | 2.33E-09 |
| CKSJ-VS-D15SJ | response to metal ion | 75 out of 549 genes, 13.7% | 306 out of 4250 genes, 7.2% | 6.66E-09 |
| CKSJ-VS-D15SJ | oxidation-reduction process | 134 out of 549 genes, 24.4% | 674 out of 4250 genes, 15.9% | 1.54E-08 |
| CKSJ-VS-D15SJ | response to chemical stimulus | 119 out of 549 genes, 21.7% | 633 out of 4250 genes, 14.9% | 3.05E-06 |
| CKSJ-VS-D15SJ | response to oxidative stress | 43 out of 549 genes, 7.8% | 173 out of 4250 genes, 4.1% | 9.74E-06 |
| CKSJ-VS-D15SJ | carbohydrate metabolic process | 85 out of 549 genes, 15.5% | 427 out of 4250 genes, 10.0% | 1.15E-05 |
| CKSJ-VS-D15SJ | response to abiotic stimulus | 101 out of 549 genes, 18.4% | 531 out of 4250 genes, 12.5% | 1.23E-05 |
| CKSJ-VS-D15SJ | small molecule metabolic process | 140 out of 549 genes, 25.5% | 808 out of 4250 genes, 19.0% | 3.54E-05 |
| CKSJ-VS-D15SJ | single-organism biosynthetic process | 62 out of 549 genes, 11.3% | 303 out of 4250 genes, 7.1% | 8.77E-05 |
| CKSJ-VS-D15SJ | cellular lipid metabolic process | 41 out of 549 genes, 7.5% | 178 out of 4250 genes, 4.2% | 0.000105 |
| CKSJ-VS-D15SJ | chorismate metabolic process | 5 out of 549 genes, 0.9% | 6 out of 4250 genes, 0.1% | 0.00019 |
| CKSJ-VS-D15SJ | monocarboxylic acid biosynthetic process | 22 out of 549 genes, 4.0% | 78 out of 4250 genes, 1.8% | 0.000228 |
| CKSJ-VS-D15SJ | response to cold | 29 out of 549 genes, 5.3% | 119 out of 4250 genes, 2.8% | 0.000403 |
| CKSJ-VS-D15SJ | terpenoid metabolic process | 15 out of 549 genes, 2.7% | 46 out of 4250 genes, 1.1% | 0.000418 |
| CKSJ-VS-D15SJ | lipid biosynthetic process | 31 out of 549 genes, 5.6% | 131 out of 4250 genes, 3.1% | 0.000446 |
| CKSJ-VS-D15SJ | organic acid biosynthetic process | 47 out of 549 genes, 8.6% | 227 out of 4250 genes, 5.3% | 0.000493 |
| CKSJ-VS-D15SJ | carboxylic acid biosynthetic process | 47 out of 549 genes, 8.6% | 227 out of 4250 genes, 5.3% | 0.000493 |
| CKSJ-VS-D15SJ | phenylpropanoid metabolic process | 14 out of 549 genes, 2.6% | 42 out of 4250 genes, 1.0% | 0.000503 |
| CKSJ-VS-D15SJ | response to temperature stimulus | 39 out of 549 genes, 7.1% | 180 out of 4250 genes, 4.2% | 0.000589 |
| CKSJ-VS-D15SJ | response to osmotic stress | 46 out of 549 genes, 8.4% | 223 out of 4250 genes, 5.2% | 0.000617 |
| CKSJ-VS-D15SJ | secondary metabolic process | 18 out of 549 genes, 3.3% | 63 out of 4250 genes, 1.5% | 0.000705 |
| CKSJ-VS-D15SJ | small molecule biosynthetic process | 55 out of 549 genes, 10.0% | 281 out of 4250 genes, 6.6% | 0.000722 |
| CKSJ-VS-D15SJ | monocarboxylic acid metabolic process | 29 out of 549 genes, 5.3% | 123 out of 4250 genes, 2.9% | 0.000723 |
| CKSJ-VS-D15SJ | nucleotide phosphorylation | 7 out of 549 genes, 1.3% | 14 out of 4250 genes, 0.3% | 0.000873 |
| CKSJ-VS-D15SJ | response to salt stress | 42 out of 549 genes, 7.7% | 204 out of 4250 genes, 4.8% | 0.001105 |
| CKSJ-VS-D15SJ | oxoacid metabolic process | 80 out of 549 genes, 14.6% | 451 out of 4250 genes, 10.6% | 0.001168 |
| CKSJ-VS-D15SJ | oxylipin metabolic process | 6 out of 549 genes, 1.1% | 11 out of 4250 genes, 0.3% | 0.001176 |
| CKSJ-VS-D15SJ | chorismate biosynthetic process | 4 out of 549 genes, 0.7% | 5 out of 4250 genes, 0.1% | 0.001237 |
| CKSJ-VS-D15SJ | shikimate biosynthetic process | 4 out of 549 genes, 0.7% | 5 out of 4250 genes, 0.1% | 0.001237 |
| CKSJ-VS-D15SJ | cellular response to water deprivation | 4 out of 549 genes, 0.7% | 5 out of 4250 genes, 0.1% | 0.001237 |
| CKSJ-VS-D15SJ | cellular response to water stimulus | 4 out of 549 genes, 0.7% | 5 out of 4250 genes, 0.1% | 0.001237 |
| CKSJ-VS-D15SJ | organic acid metabolic process | 80 out of 549 genes, 14.6% | 452 out of 4250 genes, 10.6% | 0.00125 |
| CKSJ-VS-D15SJ | terpenoid biosynthetic process | 12 out of 549 genes, 2.2% | 36 out of 4250 genes, 0.8% | 0.001259 |
| CKSJ-VS-D15SJ | lipid metabolic process | 50 out of 549 genes, 9.1% | 256 out of 4250 genes, 6.0% | 0.001326 |
| CKSJ-VS-D15SJ | response to water stimulus | 19 out of 549 genes, 3.5% | 72 out of 4250 genes, 1.7% | 0.00146 |
| CKSJ-VS-D15SJ | carboxylic acid metabolic process | 79 out of 549 genes, 14.4% | 449 out of 4250 genes, 10.6% | 0.001605 |
| CKSJ-VS-D15SJ | dicarboxylic acid metabolic process | 15 out of 549 genes, 2.7% | 52 out of 4250 genes, 1.2% | 0.00174 |
| CKSJ-VS-D15SJ | phenylpropanoid biosynthetic process | 10 out of 549 genes, 1.8% | 28 out of 4250 genes, 0.7% | 0.001767 |
| CKSJ-VS-D15SJ | metabolic process | 450 out of 549 genes, 82.0% | 3277 out of 4250 genes, 77.1% | 0.00181 |
| CKSJ-VS-D15SJ | response to hydrogen peroxide | 11 out of 549 genes, 2.0% | 33 out of 4250 genes, 0.8% | 0.001999 |
| CKSJ-VS-D15SJ | one-carbon metabolic process | 6 out of 549 genes, 1.1% | 12 out of 4250 genes, 0.3% | 0.002097 |
| CKSJ-VS-D15SJ | arginine catabolic process | 3 out of 549 genes, 0.5% | 3 out of 4250 genes, 0.1% | 0.002145 |
| CKSJ-VS-D15SJ | isoprenoid metabolic process | 16 out of 549 genes, 2.9% | 59 out of 4250 genes, 1.4% | 0.002508 |
| CKSJ-VS-D15SJ | aromatic amino acid family metabolic process | 13 out of 549 genes, 2.4% | 44 out of 4250 genes, 1.0% | 0.002754 |
| CKSJ-VS-D15SJ | secondary metabolite biosynthetic process | 13 out of 549 genes, 2.4% | 44 out of 4250 genes, 1.0% | 0.002754 |
| CKSJ-VS-D15SJ | monosaccharide metabolic process | 29 out of 549 genes, 5.3% | 134 out of 4250 genes, 3.2% | 0.002969 |
| CKSJ-VS-D15SJ | response to oxygen-containing compound | 51 out of 549 genes, 9.3% | 272 out of 4250 genes, 6.4% | 0.002983 |
| CKSJ-VS-D15SJ | cellular response to abiotic stimulus | 7 out of 549 genes, 1.3% | 17 out of 4250 genes, 0.4% | 0.003488 |
| CKSJ-VS-D15SJ | hexose metabolic process | 25 out of 549 genes, 4.6% | 112 out of 4250 genes, 2.6% | 0.003668 |
| CKSJ-VS-D15SJ | sulfur compound metabolic process | 20 out of 549 genes, 3.6% | 85 out of 4250 genes, 2.0% | 0.0048 |
| CKSJ-VS-D15SJ | oxylipin biosynthetic process | 5 out of 549 genes, 0.9% | 10 out of 4250 genes, 0.2% | 0.005092 |
| CKSJ-VS-D15SJ | fatty acid biosynthetic process | 13 out of 549 genes, 2.4% | 47 out of 4250 genes, 1.1% | 0.005174 |
| CKSJ-VS-D15SJ | cellular response to hydrogen peroxide | 6 out of 549 genes, 1.1% | 14 out of 4250 genes, 0.3% | 0.005425 |
| CKSJ-VS-D15SJ | lipid transport | 9 out of 549 genes, 1.6% | 28 out of 4250 genes, 0.7% | 0.006649 |
| CKSJ-VS-D15SJ | cellular response to nitrogen levels | 4 out of 549 genes, 0.7% | 7 out of 4250 genes, 0.2% | 0.006985 |
| CKSJ-VS-D15SJ | mannose metabolic process | 3 out of 549 genes, 0.5% | 4 out of 4250 genes, 0.1% | 0.007754 |
| CKSJ-VS-D15SJ | response to desiccation | 6 out of 549 genes, 1.1% | 15 out of 4250 genes, 0.4% | 0.008071 |
| CKSJ-VS-D15SJ | serine family amino acid biosynthetic process | 8 out of 549 genes, 1.5% | 24 out of 4250 genes, 0.6% | 0.008146 |
| CKSJ-VS-D15SJ | aromatic amino acid family biosynthetic process | 8 out of 549 genes, 1.5% | 24 out of 4250 genes, 0.6% | 0.008146 |
| CKSJ-VS-D15SJ | ethanolamine-containing compound metabolic process | 5 out of 549 genes, 0.9% | 11 out of 4250 genes, 0.3% | 0.008355 |
| CKSJ-VS-D15SJ | hydrogen peroxide catabolic process | 5 out of 549 genes, 0.9% | 11 out of 4250 genes, 0.3% | 0.008355 |
| CKSJ-VS-D15SJ | lipid localization | 9 out of 549 genes, 1.6% | 29 out of 4250 genes, 0.7% | 0.008565 |
| CKSJ-VS-D15SJ | generation of precursor metabolites and energy | 43 out of 549 genes, 7.8% | 234 out of 4250 genes, 5.5% | 0.008957 |
| CKSJ-VS-D15SJ | isoprenoid biosynthetic process | 13 out of 549 genes, 2.4% | 50 out of 4250 genes, 1.2% | 0.00906 |
| CKSJ-VS-D15SJ | single-organism carbohydrate metabolic process | 46 out of 549 genes, 8.4% | 254 out of 4250 genes, 6.0% | 0.009145 |
| CKSJ-VS-D15SJ | response to bacterium | 26 out of 549 genes, 4.7% | 126 out of 4250 genes, 3.0% | 0.009168 |
| CKSJ-VS-D15SJ | response to water deprivation | 16 out of 549 genes, 2.9% | 67 out of 4250 genes, 1.6% | 0.009581 |
| CKSJ-VS-D15SJ | cellular amino acid biosynthetic process | 30 out of 549 genes, 5.5% | 152 out of 4250 genes, 3.6% | 0.010233 |
| CKSJ-VS-D15SJ | glycolysis | 15 out of 549 genes, 2.7% | 62 out of 4250 genes, 1.5% | 0.010568 |
| CKSJ-VS-D15SJ | cellular response to reactive oxygen species | 6 out of 549 genes, 1.1% | 16 out of 4250 genes, 0.4% | 0.011531 |
| CKSJ-VS-D15SJ | phytoalexin metabolic process | 6 out of 549 genes, 1.1% | 16 out of 4250 genes, 0.4% | 0.011531 |
| CKSJ-VS-D15SJ | shikimate metabolic process | 4 out of 549 genes, 0.7% | 8 out of 4250 genes, 0.2% | 0.012556 |
| CKSJ-VS-D15SJ | lignin metabolic process | 5 out of 549 genes, 0.9% | 12 out of 4250 genes, 0.3% | 0.012825 |
| CKSJ-VS-D15SJ | hydrogen peroxide metabolic process | 5 out of 549 genes, 0.9% | 12 out of 4250 genes, 0.3% | 0.012825 |
| CKSJ-VS-D15SJ | dicarboxylic acid biosynthetic process | 5 out of 549 genes, 0.9% | 12 out of 4250 genes, 0.3% | 0.012825 |
| CKSJ-VS-D15SJ | diterpene phytoalexin metabolic process | 5 out of 549 genes, 0.9% | 12 out of 4250 genes, 0.3% | 0.012825 |
| CKSJ-VS-D15SJ | carbohydrate catabolic process | 23 out of 549 genes, 4.2% | 111 out of 4250 genes, 2.6% | 0.013136 |
| CKSJ-VS-D15SJ | fatty acid metabolic process | 16 out of 549 genes, 2.9% | 70 out of 4250 genes, 1.6% | 0.014631 |
| CKSJ-VS-D15SJ | alcohol metabolic process | 13 out of 549 genes, 2.4% | 53 out of 4250 genes, 1.2% | 0.01493 |
| CKSJ-VS-D15SJ | glucose metabolic process | 20 out of 549 genes, 3.6% | 94 out of 4250 genes, 2.2% | 0.015098 |
| CKSJ-VS-D15SJ | glucose catabolic process | 19 out of 549 genes, 3.5% | 88 out of 4250 genes, 2.1% | 0.015135 |
| CKSJ-VS-D15SJ | hexose catabolic process | 19 out of 549 genes, 3.5% | 88 out of 4250 genes, 2.1% | 0.015135 |
| CKSJ-VS-D15SJ | monosaccharide catabolic process | 19 out of 549 genes, 3.5% | 88 out of 4250 genes, 2.1% | 0.015135 |
| CKSJ-VS-D15SJ | amine metabolic process | 14 out of 549 genes, 2.6% | 59 out of 4250 genes, 1.4% | 0.015742 |
| CKSJ-VS-D15SJ | cellular response to nitrogen starvation | 2 out of 549 genes, 0.4% | 2 out of 4250 genes, 0.0% | 0.01666 |
| CKSJ-VS-D15SJ | suberin biosynthetic process | 2 out of 549 genes, 0.4% | 2 out of 4250 genes, 0.0% | 0.01666 |
| CKSJ-VS-D15SJ | GDP-mannose metabolic process | 2 out of 549 genes, 0.4% | 2 out of 4250 genes, 0.0% | 0.01666 |
| CKSJ-VS-D15SJ | choline metabolic process | 2 out of 549 genes, 0.4% | 2 out of 4250 genes, 0.0% | 0.01666 |
| CKSJ-VS-D15SJ | organic hydroxy compound metabolic process | 13 out of 549 genes, 2.4% | 54 out of 4250 genes, 1.3% | 0.017423 |
| CKSJ-VS-D15SJ | AMP biosynthetic process | 3 out of 549 genes, 0.5% | 5 out of 4250 genes, 0.1% | 0.017528 |
| CKSJ-VS-D15SJ | AMP metabolic process | 3 out of 549 genes, 0.5% | 5 out of 4250 genes, 0.1% | 0.017528 |
| CKSJ-VS-D15SJ | hydrogen peroxide transmembrane transport | 3 out of 549 genes, 0.5% | 5 out of 4250 genes, 0.1% | 0.017528 |
| CKSJ-VS-D15SJ | response to wounding | 11 out of 549 genes, 2.0% | 43 out of 4250 genes, 1.0% | 0.017836 |
| CKSJ-VS-D15SJ | response to red or far red light | 11 out of 549 genes, 2.0% | 43 out of 4250 genes, 1.0% | 0.017836 |
| CKSJ-VS-D15SJ | water transport | 5 out of 549 genes, 0.9% | 13 out of 4250 genes, 0.3% | 0.018669 |
| CKSJ-VS-D15SJ | fluid transport | 5 out of 549 genes, 0.9% | 13 out of 4250 genes, 0.3% | 0.018669 |
| CKSJ-VS-D15SJ | lignin biosynthetic process | 4 out of 549 genes, 0.7% | 9 out of 4250 genes, 0.2% | 0.020324 |
| CKSJ-VS-D15SJ | negative regulation of transcription, DNA-dependent | 4 out of 549 genes, 0.7% | 9 out of 4250 genes, 0.2% | 0.020324 |
| CKSJ-VS-D15SJ | negative regulation of RNA metabolic process | 4 out of 549 genes, 0.7% | 9 out of 4250 genes, 0.2% | 0.020324 |
| CKSJ-VS-D15SJ | sulfur compound biosynthetic process | 14 out of 549 genes, 2.6% | 61 out of 4250 genes, 1.4% | 0.020903 |
| CKSJ-VS-D15SJ | cellular response to extracellular stimulus | 11 out of 549 genes, 2.0% | 44 out of 4250 genes, 1.0% | 0.021106 |
| CKSJ-VS-D15SJ | cellular response to external stimulus | 11 out of 549 genes, 2.0% | 44 out of 4250 genes, 1.0% | 0.021106 |
| CKSJ-VS-D15SJ | diterpenoid metabolic process | 6 out of 549 genes, 1.1% | 18 out of 4250 genes, 0.4% | 0.021335 |
| CKSJ-VS-D15SJ | negative regulation of cellular metabolic process | 6 out of 549 genes, 1.1% | 18 out of 4250 genes, 0.4% | 0.021335 |
| CKSJ-VS-D15SJ | cellular response to oxidative stress | 6 out of 549 genes, 1.1% | 18 out of 4250 genes, 0.4% | 0.021335 |
| CKSJ-VS-D15SJ | response to zinc ion | 8 out of 549 genes, 1.5% | 28 out of 4250 genes, 0.7% | 0.021601 |
| CKSJ-VS-D15SJ | cellular metabolic compound salvage | 8 out of 549 genes, 1.5% | 28 out of 4250 genes, 0.7% | 0.021601 |
| CKSJ-VS-D15SJ | response to reactive oxygen species | 13 out of 549 genes, 2.4% | 56 out of 4250 genes, 1.3% | 0.023339 |
| CKSJ-VS-D15SJ | serine family amino acid metabolic process | 9 out of 549 genes, 1.6% | 34 out of 4250 genes, 0.8% | 0.024905 |
| CKSJ-VS-D15SJ | hyperosmotic response | 5 out of 549 genes, 0.9% | 14 out of 4250 genes, 0.3% | 0.026027 |
| CKSJ-VS-D15SJ | glutamate metabolic process | 4 out of 549 genes, 0.7% | 10 out of 4250 genes, 0.2% | 0.030479 |
| CKSJ-VS-D15SJ | nucleotide-sugar metabolic process | 4 out of 549 genes, 0.7% | 10 out of 4250 genes, 0.2% | 0.030479 |
| CKSJ-VS-D15SJ | hexose biosynthetic process | 4 out of 549 genes, 0.7% | 10 out of 4250 genes, 0.2% | 0.030479 |
| CKSJ-VS-D15SJ | phosphatidylcholine metabolic process | 4 out of 549 genes, 0.7% | 10 out of 4250 genes, 0.2% | 0.030479 |
| CKSJ-VS-D15SJ | sphingolipid metabolic process | 3 out of 549 genes, 0.5% | 6 out of 4250 genes, 0.1% | 0.031723 |
| CKSJ-VS-D15SJ | nucleotide-sugar biosynthetic process | 3 out of 549 genes, 0.5% | 6 out of 4250 genes, 0.1% | 0.031723 |
| CKSJ-VS-D15SJ | response to extracellular stimulus | 11 out of 549 genes, 2.0% | 47 out of 4250 genes, 1.1% | 0.033543 |
| CKSJ-VS-D15SJ | response to karrikin | 11 out of 549 genes, 2.0% | 47 out of 4250 genes, 1.1% | 0.033543 |
| CKSJ-VS-D15SJ | pollen tube growth | 7 out of 549 genes, 1.3% | 25 out of 4250 genes, 0.6% | 0.034369 |
| CKSJ-VS-D15SJ | cellular response to stress | 22 out of 549 genes, 4.0% | 115 out of 4250 genes, 2.7% | 0.035457 |
| CKSJ-VS-D15SJ | single-organism carbohydrate catabolic process | 19 out of 549 genes, 3.5% | 96 out of 4250 genes, 2.3% | 0.035584 |
| CKSJ-VS-D15SJ | response to other organism | 39 out of 549 genes, 7.1% | 228 out of 4250 genes, 5.4% | 0.036661 |
| CKSJ-VS-D15SJ | response to biotic stimulus | 40 out of 549 genes, 7.3% | 235 out of 4250 genes, 5.5% | 0.037116 |
| CKSJ-VS-D15SJ | phospholipid biosynthetic process | 7 out of 549 genes, 1.3% | 26 out of 4250 genes, 0.6% | 0.041984 |
| CKSJ-VS-D15SJ | pyruvate metabolic process | 4 out of 549 genes, 0.7% | 11 out of 4250 genes, 0.3% | 0.043122 |
| CKSJ-VS-D15SJ | gibberellin metabolic process | 4 out of 549 genes, 0.7% | 11 out of 4250 genes, 0.3% | 0.043122 |
| CKSJ-VS-D15SJ | negative regulation of biosynthetic process | 4 out of 549 genes, 0.7% | 11 out of 4250 genes, 0.3% | 0.043122 |
| CKSJ-VS-D15SJ | negative regulation of macromolecule biosynthetic process | 4 out of 549 genes, 0.7% | 11 out of 4250 genes, 0.3% | 0.043122 |
| CKSJ-VS-D15SJ | negative regulation of cellular biosynthetic process | 4 out of 549 genes, 0.7% | 11 out of 4250 genes, 0.3% | 0.043122 |
| CKSJ-VS-D15SJ | hyperosmotic salinity response | 4 out of 549 genes, 0.7% | 11 out of 4250 genes, 0.3% | 0.043122 |
| CKSJ-VS-D15SJ | amide transport | 4 out of 549 genes, 0.7% | 11 out of 4250 genes, 0.3% | 0.043122 |
| CKSJ-VS-D15SJ | negative regulation of nucleobase-containing compound metabolic process | 4 out of 549 genes, 0.7% | 11 out of 4250 genes, 0.3% | 0.043122 |
| CKSJ-VS-D15SJ | negative regulation of nitrogen compound metabolic process | 4 out of 549 genes, 0.7% | 11 out of 4250 genes, 0.3% | 0.043122 |
| CKSJ-VS-D15SJ | negative regulation of cellular macromolecule biosynthetic process | 4 out of 549 genes, 0.7% | 11 out of 4250 genes, 0.3% | 0.043122 |
| CKSJ-VS-D15SJ | photomorphogenesis | 6 out of 549 genes, 1.1% | 21 out of 4250 genes, 0.5% | 0.044599 |
| CKSJ-VS-D15SJ | cellular carbohydrate metabolic process | 26 out of 549 genes, 4.7% | 144 out of 4250 genes, 3.4% | 0.04509 |
| CKSJ-VS-D15SJ | pyrimidine nucleobase catabolic process | 2 out of 549 genes, 0.4% | 3 out of 4250 genes, 0.1% | 0.04569 |
| CKSJ-VS-D15SJ | uracil catabolic process | 2 out of 549 genes, 0.4% | 3 out of 4250 genes, 0.1% | 0.04569 |
| CKSJ-VS-D15SJ | ornithine metabolic process | 2 out of 549 genes, 0.4% | 3 out of 4250 genes, 0.1% | 0.04569 |
| CKSJ-VS-D15SJ | urea transport | 2 out of 549 genes, 0.4% | 3 out of 4250 genes, 0.1% | 0.04569 |
| CKSJ-VS-D15SJ | tetrahydrofolate interconversion | 2 out of 549 genes, 0.4% | 3 out of 4250 genes, 0.1% | 0.04569 |
| CKSJ-VS-D15SJ | nucleobase catabolic process | 2 out of 549 genes, 0.4% | 3 out of 4250 genes, 0.1% | 0.04569 |
| CKSJ-VS-D15SJ | urea transmembrane transport | 2 out of 549 genes, 0.4% | 3 out of 4250 genes, 0.1% | 0.04569 |
| CKSJ-VS-D15SJ | multi-organism process | 47 out of 549 genes, 8.6% | 287 out of 4250 genes, 6.8% | 0.046024 |
| CKSJ-VS-D15SJ | photosynthesis | 14 out of 549 genes, 2.6% | 68 out of 4250 genes, 1.6% | 0.049129 |

**Table S6.** A detailed list of TFs expressed differentially in CKDN vs.D15DN under low -T_w_ treatment.

| GeneID | Length | CKDN-Expression | D15DN-Expression | log2FoldChange(D15DN/CKDN) | Pvalue | TF_family |
| --- | --- | --- | --- | --- | --- | --- |
| 4346629 | 1635 | 98.61019 | 559.5859 | 2.504551 | 4.88E-19 | AP2-EREBP |
| 9270656 | 1400 | 896.243 | 412.9974 | -1.11776 | 8.76E-07 | C3H |
| 4326683 | 1454 | 446.9447 | 29.24967 | -3.9336 | 5.56E-20 | WRKY |
| 4329852 | 1938 | 176.2414 | 68.07876 | -1.37228 | 5.10E-05 | NAC |
| 4327245 | 1794 | 26.29431 | 143.4156 | 2.44738 | 1.09E-10 | SBP |
| 4341882 | 2885 | 165.5093 | 519.8045 | 1.651057 | 1.65E-12 | ARF |
| 4336779 | 2024 | 83.37557 | 206.2639 | 1.306794 | 1.40E-07 | MYB |
| 4344705 | 2926 | 7840.634 | 1840.058 | -2.09122 | 2.88E-34 | NAC |
| 4342416 | 2227 | 113.1202 | 242.606 | 1.100758 | 0.00058 | TCP |
| 4324958 | 1643 | 1044.052 | 377.9157 | -1.46606 | 5.54E-06 | TAZ |
| 4325520 | 1128 | 105.0387 | 210.1693 | 1.000632 | 0.002754 | NOZZLE |
| 4341678 | 1652 | 5105.902 | 1098.499 | -2.21663 | 3.72E-16 | WRKY |
| 4334444 | 1214 | 995.5902 | 283.4962 | -1.81222 | 1.49E-12 | Alfin-like |
| 1.07E+08 | 1584 | 35.57401 | 119.7796 | 1.751486 | 0.000201 | SBP |
| 4335698 | 964 | 270.8828 | 108.1359 | -1.32482 | 0.006633 | Tify |
| 4330280 | 2611 | 80.42762 | 246.5535 | 1.616138 | 0.000972 | C2H2 |
| 1.07E+08 | 2126 | 3.974994 | 39.22837 | 3.302873 | 2.94E-05 | C2H2 |
| BGI_novel_G000104 | 1626 | 24.26693 | 0.651115 | -5.21994 | 1.92E-08 | WRKY |
| 4348901 | 1600 | 5.659559 | 27.5303 | 2.282259 | 0.000904 | NAC |
| 4331833 | 1141 | 8304.153 | 2003.573 | -2.05126 | 9.71E-09 | Tify |
| 4334553 | 1345 | 38033.56 | 3951.228 | -3.2669 | 7.79E-76 | NAC |
| 4337752 | 1894 | 19.39837 | 61.18151 | 1.657161 | 0.000379 | bHLH |
| 1.07E+08 | 1486 | 3655.295 | 49.00593 | -6.22089 | 2.18E-78 | AP2-EREBP |
| 4330864 | 2262 | 812.2067 | 207.8156 | -1.96654 | 6.37E-23 | WRKY |
| 4333948 | 2528 | 1774.53 | 226.3698 | -2.97068 | 1.74E-52 | GRAS |
| 4333197 | 1100 | 2461.728 | 479.6438 | -2.35964 | 7.35E-39 | C2H2 |
| 4334864 | 962 | 87.42194 | 241.0781 | 1.463433 | 6.93E-05 | AP2-EREBP |
| 4351131 | 1474 | 176.166 | 371.4731 | 1.076322 | 7.23E-05 | MYB |
| 4334592 | 908 | 19259.61 | 824.0621 | -4.54668 | 2.05E-58 | C2H2 |
| 4341986 | 2189 | 80.76192 | 423.2747 | 2.389847 | 4.03E-07 | SBP |
| 4347359 | 2207 | 1400.785 | 617.0404 | -1.1828 | 8.81E-12 | bHLH |
| 4332845 | 2430 | 125.0833 | 252.3455 | 1.012511 | 7.46E-05 | TUB |
| 1.07E+08 | 2816 | 48.69128 | 6.249124 | -2.96194 | 1.54E-08 | bHLH |
| 4335707 | 3113 | 1163.157 | 436.1377 | -1.41519 | 0.00011 | AP2-EREBP |
| 4350864 | 1475 | 431.8701 | 163.3288 | -1.40282 | 7.71E-08 | bHLH |
| 4334599 | 3014 | 130.0947 | 268.8516 | 1.047247 | 0.000178 | C2C2-Dof |
| BGI_novel_G000434 | 970 | 18.69312 | 1.603219 | -3.54346 | 3.42E-05 | AP2-EREBP |
| 4326726 | 1139 | 5870.367 | 531.2161 | -3.46608 | 1.07E-54 | MYB |
| 9271635 | 2133 | 34.5929 | 3.099067 | -3.48057 | 6.19E-05 | NOZZLE |
| 4351370 | 1753 | 519.31 | 103.808 | -2.32268 | 3.24E-10 | NAC |
| 1.07E+08 | 1903 | 118.3179 | 263.1677 | 1.153314 | 0.000122 | WRKY |
| 4339665 | 1102 | 669.9649 | 69.02855 | -3.27882 | 2.90E-21 | WRKY |
| 4341990 | 1951 | 33.53578 | 160.9365 | 2.262719 | 1.21E-12 | G2-like |
| 4342538 | 3684 | 3027.304 | 836.1323 | -1.85623 | 1.43E-32 | CPP |
| 4350143 | 2448 | 151.5267 | 56.67641 | -1.41875 | 1.50E-05 | Alfin-like |
| 4328461 | 1580 | 67.0435 | 241.0392 | 1.846098 | 2.51E-13 | SBP |
| 4351577 | 2852 | 550.2927 | 160.5319 | -1.77734 | 3.41E-13 | GRAS |
| 4333910 | 2601 | 112.8362 | 30.6149 | -1.88192 | 2.75E-08 | Alfin-like |
| 9268771 | 2220 | 3035.373 | 230.0706 | -3.72173 | 1.56E-68 | AP2-EREBP |
| 4332113 | 1723 | 1942.771 | 462.9687 | -2.06913 | 5.23E-15 | HSF |
| 4332755 | 1877 | 2433.474 | 192.7563 | -3.65817 | 1.77E-40 | WRKY |
| 4349650 | 1704 | 210.081 | 53.10962 | -1.9839 | 2.78E-07 | NAC |
| 4331887 | 1314 | 716.0254 | 165.3254 | -2.1147 | 4.68E-07 | bHLH |
| 9269465 | 1025 | 10481.67 | 241.6456 | -5.43883 | 8.30E-33 | bHLH |
| 4329405 | 1862 | 159.5039 | 324.2609 | 1.023563 | 1.51E-05 | HSF |
| 1.07E+08 | 1655 | 93.4527 | 298.4994 | 1.67542 | 7.64E-05 | NAC |
| 4342759 | 1465 | 1614.022 | 695.1741 | -1.21521 | 9.20E-08 | AP2-EREBP |
| 4342383 | 2192 | 17.47219 | 53.18082 | 1.605846 | 0.000896 | bHLH |
| 1.07E+08 | 1694 | 362.8129 | 58.62195 | -2.62971 | 2.76E-12 | WRKY |
| 4336288 | 1584 | 336.7439 | 73.2766 | -2.20023 | 4.66E-08 | C2C2-CO-like |
| 4338926 | 1170 | 1837.271 | 152.1015 | -3.59446 | 3.61E-37 | NAC |
| 4333315 | 1819 | 10.22376 | 31.47227 | 1.622155 | 0.003604 | MYB |
| 4327076 | 1490 | 1401.898 | 575.0918 | -1.28552 | 4.46E-17 | BES1 |
| 4333065 | 1251 | 4675.07 | 2213.238 | -1.07883 | 9.94E-05 | Tify |
| 4337756 | 3724 | 14.30788 | 60.35952 | 2.076771 | 0.002097 | MYB |
| 4344331 | 2405 | 4814.424 | 1399.308 | -1.78265 | 7.79E-07 | EIL |
| 9267785 | 1633 | 77.21145 | 23.01308 | -1.74636 | 0.005435 | AP2-EREBP |
| 4343868 | 1256 | 1309.961 | 548.9722 | -1.25472 | 4.81E-06 | Alfin-like |
| 4337599 | 1867 | 12.76735 | 37.33576 | 1.548099 | 0.005552 | C2C2-Dof |
| 4352574 | 2021 | 5.743668 | 28.88453 | 2.330253 | 0.00022 | C2C2-Dof |
| 9267508 | 1158 | 875.8622 | 111.7906 | -2.9699 | 1.13E-12 | WRKY |
| 4351328 | 1340 | 89.38151 | 41.55479 | -1.10496 | 0.005397 | WRKY |
| 4347070 | 1248 | 2381.3 | 240.1309 | -3.30986 | 1.67E-29 | WRKY |
| 4349814 | 1170 | 182.1213 | 10.1037 | -4.17194 | 5.73E-29 | NAC |
| 1.07E+08 | 1138 | 113.2224 | 15.78064 | -2.84293 | 0.001001 | WRKY |
| 4330722 | 2390 | 55.79047 | 171.9109 | 1.623571 | 0.00179 | TCP |
| 4325181 | 1677 | 80.75629 | 249.5482 | 1.627672 | 0.000311 | MYB |
| 4339490 | 1535 | 143.9687 | 445.4147 | 1.629394 | 0.005786 | MYB |
| 4326803 | 2245 | 287.9636 | 86.00826 | -1.74334 | 1.33E-06 | NAC |
| 4337692 | 2335 | 29641.62 | 6194.975 | -2.25845 | 7.33E-34 | C3H |
| 4331627 | 910 | 13.43916 | 88.85058 | 2.724938 | 0.000563 | LOB |
| 4348616 | 2005 | 1058.717 | 2128.942 | 1.00782 | 1.39E-06 | NAC |
| 9270349 | 2122 | 34847.8 | 1896.492 | -4.19966 | 3.82E-17 | AP2-EREBP |
| 4333691 | 1774 | 3.973699 | 79.15133 | 4.316059 | 1.83E-07 | bHLH |
| 9266609 | 1513 | 74.50603 | 205.7152 | 1.465219 | 1.97E-09 | mTERF |
| 4342999 | 3216 | 24.52455 | 66.14709 | 1.431451 | 0.001198 | mTERF |
| BGI_novel_G000419 | 2277 | 39.57192 | 8.541947 | -2.21184 | 0.000244 | AP2-EREBP |
| 4333934 | 1482 | 39.04913 | 263.1415 | 2.752476 | 5.89E-23 | MYB |
| 4341999 | 5625 | 696.8651 | 229.1668 | -1.60448 | 2.90E-05 | TAZ |
| 4337015 | 1640 | 158.4144 | 339.4985 | 1.099702 | 5.77E-07 | bHLH |
| 9269685 | 2343 | 42.67474 | 108.8758 | 1.351229 | 0.000349 | GRAS |
| 4329655 | 1129 | 8.197816 | 33.64604 | 2.037125 | 0.001403 | NAC |
| 4352661 | 1606 | 85.62525 | 402.4935 | 2.232857 | 6.97E-07 | G2-like |
| 4343653 | 1367 | 1045.906 | 186.3823 | -2.48842 | 2.09E-34 | C2H2 |
| 4326230 | 2355 | 672.5016 | 275.6574 | -1.28666 | 4.96E-10 | C3H |
| 4348439 | 2208 | 2508.144 | 857.3992 | -1.54858 | 1.47E-17 | NAC |
| 1.07E+08 | 1521 | 3.082768 | 26.10045 | 3.081776 | 3.75E-05 | OFP |
| 4338934 | 1824 | 1237.771 | 459.9408 | -1.42822 | 4.57E-10 | C2H2 |
| 4347142 | 1631 | 10245.12 | 21125.77 | 1.044068 | 0.000124 | AP2-EREBP |
| 4341753 | 1463 | 951.3591 | 261.1216 | -1.86527 | 6.44E-19 | bZIP |
| 4339928 | 1660 | 14.52208 | 67.60886 | 2.218964 | 5.92E-07 | GRF |
| 4325916 | 1541 | 77.71363 | 280.1902 | 1.850167 | 0.002202 | C3H |
| 4336519 | 1348 | 579.0025 | 30.38314 | -4.25223 | 1.06E-25 | NAC |
| 4343764 | 1068 | 36.50356 | 116.9354 | 1.679603 | 4.58E-06 | C2H2 |
| 4332331 | 1102 | 186.1969 | 32.96487 | -2.49783 | 3.32E-09 | AP2-EREBP |
| 1.07E+08 | 4217 | 20.9117 | 79.8856 | 1.933625 | 0.000224 | mTERF |
| 4331600 | 1538 | 401.3904 | 105.6365 | -1.9259 | 2.06E-17 | C2C2-GATA |
| 4332403 | 2781 | 6606.139 | 1210.364 | -2.44837 | 1.42E-57 | MYB |
| 4330829 | 1187 | 15401.42 | 574.6691 | -4.74419 | 5.81E-36 | AP2-EREBP |
| 4330349 | 2103 | 28.28996 | 73.17011 | 1.370964 | 0.004019 | MYB |
| 4328265 | 4290 | 19.61591 | 49.66946 | 1.340335 | 0.001055 | ARF |
| 4325454 | 2089 | 271.243 | 814.7335 | 1.586742 | 5.08E-13 | MYB |
| 4341326 | 1835 | 199.4816 | 20.21937 | -3.30245 | 2.09E-10 | HSF |
| 4329309 | 1101 | 2292.029 | 73.86861 | -4.95552 | 3.80E-34 | WRKY |
| 4345899 | 2085 | 1869.428 | 933.3812 | -1.00206 | 6.29E-05 | bHLH |
| 1.07E+08 | 1970 | 10.47769 | 1.876617 | -2.48111 | 0.006115 | GRAS |
| 9268190 | 1509 | 2673.08 | 134.1328 | -4.31677 | 4.00E-34 | MYB |
| 9266067 | 1829 | 164.1637 | 19.06037 | -3.10649 | 1.71E-08 | WRKY |
| 4336759 | 2440 | 2468.459 | 1046.171 | -1.23849 | 4.41E-07 | GRAS |
| 9268836 | 2003 | 2262.601 | 45.97099 | -5.62111 | 2.27E-30 | WRKY |
| 4344289 | 1339 | 4.476521 | 34.81604 | 2.959302 | 9.00E-07 | OFP |
| 4340317 | 2126 | 884.4336 | 272.4786 | -1.69861 | 2.52E-16 | LIM |
| 4338474 | 2090 | 15469.24 | 5890.308 | -1.39299 | 1.63E-11 | WRKY |
| 9270511 | 1806 | 21.2611 | 79.12589 | 1.895934 | 7.40E-06 | C2H2 |
| 4330230 | 2918 | 148.3262 | 341.104 | 1.201438 | 1.25E-05 | GRAS |
| 4347669 | 1687 | 367.627 | 159.8948 | -1.20112 | 2.60E-05 | SRS |
| 4333464 | 4124 | 70.76999 | 160.8289 | 1.184317 | 0.005268 | ABI3VP1 |
| 4346023 | 4563 | 5335.749 | 2285.089 | -1.22344 | 3.78E-14 | SBP |
| 4340382 | 2011 | 1967.449 | 940.4133 | -1.06496 | 2.78E-07 | bHLH |
| 4338039 | 3622 | 55.6968 | 490.9122 | 3.139799 | 5.45E-36 | MYB |
| 4342753 | 1330 | 4266.353 | 712.7241 | -2.58159 | 8.64E-18 | NAC |
| 1.07E+08 | 762 | 61.51394 | 3.521346 | -4.12671 | 8.30E-12 | WRKY |
| 4347266 | 1087 | 12061.36 | 230.4228 | -5.70996 | 2.49E-60 | AP2-EREBP |
| 1.07E+08 | 1262 | 17.07103 | 1.954307 | -3.12682 | 0.000171 | NAC |
| 4339092 | 2288 | 3042.851 | 143.6339 | -4.40496 | 1.89E-34 | WRKY |
| 4352559 | 1204 | 365.6958 | 1360.822 | 1.895762 | 6.75E-09 | MYB |
| 4334471 | 1283 | 40.55349 | 16.63884 | -1.28527 | 0.007519 | bHLH |
| 4331412 | 713 | 37.86459 | 6.391162 | -2.5667 | 1.24E-05 | AP2-EREBP |
| 4325120 | 2005 | 7936.907 | 1208.491 | -2.71537 | 1.00E-34 | NAC |
| 9266399 | 1183 | 3157.289 | 1363.596 | -1.21127 | 1.51E-05 | WRKY |
| 9271723 | 863 | 76.4876 | 217.7873 | 1.509622 | 4.18E-08 | ABI3VP1 |
| 4344665 | 1775 | 17.96937 | 63.40626 | 1.819085 | 0.005978 | MYB |
| 4331725 | 2834 | 40.31039 | 102.5645 | 1.347307 | 5.97E-05 | FAR1 |
| 4325035 | 2761 | 5.883482 | 19.72627 | 1.745376 | 0.012241 | AP2-EREBP |
| 9272091 | 1120 | 20.97484 | 111.7051 | 2.412963 | 8.01E-07 | MYB |
| 9269795 | 1573 | 64.48827 | 291.0846 | 2.17433 | 8.85E-13 | C2C2-CO-like |
| 4343630 | 2285 | 96.64519 | 4.876536 | -4.30877 | 2.55E-14 | NAC |
| 4335808 | 2169 | 661.0294 | 132.7164 | -2.31637 | 3.21E-22 | E2F-DP |
| 4334294 | 1545 | 34.55854 | 103.6666 | 1.584838 | 2.40E-05 | NAC |
| 4333157 | 2457 | 2646.447 | 1110.527 | -1.25281 | 7.24E-12 | MYB |
| 4339597 | 2131 | 610.8717 | 173.1782 | -1.81861 | 1.99E-09 | TUB |
| 4337494 | 1933 | 10.01803 | 134.9747 | 3.752018 | 4.20E-07 | C2H2 |
| 4330217 | 1469 | 26.24431 | 3.223058 | -3.0255 | 5.80E-06 | C2H2 |
| 1.07E+08 | 858 | 1827.026 | 47.00778 | -5.28045 | 2.31E-62 | AP2-EREBP |
| 4344297 | 5427 | 768.6849 | 280.6506 | -1.45362 | 1.99E-06 | Alfin-like |
| 4331648 | 1780 | 1142.538 | 203.8119 | -2.48693 | 2.55E-22 | E2F-DP |
| 9271993 | 1156 | 1.898886 | 14.02177 | 2.884444 | 0.000843 | LOB |
| 4344021 | 1738 | 26.27279 | 91.69999 | 1.803352 | 0.001174 | MYB |
| 4334668 | 1312 | 320.787 | 655.291 | 1.03052 | 2.56E-05 | C2C2-GATA |
| 4339978 | 6987 | 763.7175 | 341.3951 | -1.1616 | 1.48E-05 | GRAS |
| 4343641 | 2664 | 2483.421 | 1240.727 | -1.00114 | 1.80E-06 | C3H |
| 4328230 | 2572 | 70.40116 | 26.58715 | -1.40487 | 0.0033 | RWP-RK |
| 4344327 | 1674 | 81.5227 | 210.3409 | 1.367455 | 0.004862 | G2-like |
| 1.07E+08 | 1139 | 2.547303 | 10.97198 | 2.106782 | 0.013134 | MYB |
| 4325718 | 1931 | 80.25357 | 24.87023 | -1.69015 | 4.30E-06 | MYB |
| 4339974 | 1096 | 30315.64 | 547.1894 | -5.79188 | ###### | AP2-EREBP |
| 4339863 | 1571 | 156.6346 | 324.1296 | 1.049168 | 0.000243 | MYB |
| 4330202 | 1534 | 117.8432 | 434.8981 | 1.883809 | 1.02E-17 | AP2-EREBP |
| 4347620 | 950 | 2509.206 | 944.8293 | -1.40911 | 3.78E-07 | AP2-EREBP |
| 4325657 | 1897 | 2150.932 | 746.8543 | -1.52606 | 2.15E-09 | WRKY |
| 4330436 | 1624 | 5.408535 | 0.570753 | -3.2443 | 0.001956 | GRF |
| 1.07E+08 | 624 | 6.248855 | 0.636287 | -3.29584 | 0.001671 | AP2-EREBP |
| 4339151 | 1399 | 9.655166 | 27.6079 | 1.515708 | 0.007839 | G2-like |
| 9271572 | 1321 | 5.36803 | 34.84785 | 2.698605 | 1.22E-05 | MADS |
| 4331765 | 1967 | 188.5834 | 542.6038 | 1.524697 | 7.75E-08 | C2C2-Dof |
| 4345659 | 1794 | 311.8162 | 955.1743 | 1.615068 | 6.54E-08 | G2-like |
| 9270639 | 2582 | 26.03201 | 107.0527 | 2.039963 | 7.44E-05 | SBP |
| 4344514 | 1776 | 39.88052 | 175.3144 | 2.136188 | 2.82E-05 | NAC |
| 4336872 | 1527 | 133.517 | 36.78061 | -1.86001 | 0.000176 | G2-like |
| 4334261 | 2252 | 15.68461 | 4.098339 | -1.93624 | 0.004737 | HB |
| 4330001 | 1458 | 945.3424 | 211.9915 | -2.15683 | 4.39E-12 | MYB |
| 4328953 | 1959 | 1851.489 | 502.0219 | -1.88286 | 9.11E-17 | WRKY |
| 4336500 | 1653 | 57.50231 | 187.5936 | 1.705919 | 0.00192 | MYB |
| 4340383 | 1577 | 1734.286 | 3877.046 | 1.160616 | 0.000373 | AP2-EREBP |
| 4347619 | 1060 | 4728.252 | 529.3771 | -3.15894 | 3.77E-17 | AP2-EREBP |
| 4327261 | 1823 | 387.3472 | 1022.698 | 1.400681 | 1.58E-15 | TCP |
| 4334213 | 1258 | 28547.12 | 4774.762 | -2.57984 | 7.52E-30 | C2H2 |
| 9268095 | 1946 | 506.7716 | 158.9842 | -1.67245 | 6.87E-06 | WRKY |
| 4338832 | 1511 | 1779.407 | 308.0181 | -2.53031 | 1.78E-15 | NAC |
| 4329629 | 2507 | 68.19326 | 138.3918 | 1.021057 | 0.001291 | FAR1 |
| BGI_novel_G000398 | 1156 | 48.78974 | 12.44901 | -1.97055 | 0.010636 | MYB |
| 4330466 | 3756 | 170.5922 | 26.28418 | -2.69828 | 4.80E-09 | zf-HD |
| 4344326 | 1881 | 80.98074 | 186.806 | 1.20589 | 2.17E-06 | C2C2-Dof |
| 4326856 | 2307 | 1204.719 | 514.4727 | -1.22753 | 4.23E-05 | WRKY |
| 1.07E+08 | 1964 | 81.72477 | 23.77576 | -1.78128 | 0.000417 | GRAS |
| 4333810 | 3314 | 2546.32 | 1144.369 | -1.15386 | 4.46E-14 | C3H |
| 4326760 | 2285 | 11769.61 | 1886.913 | -2.64097 | 2.30E-23 | WRKY |
| 9266268 | 1098 | 150.7013 | 7.502203 | -4.32823 | 1.52E-10 | MYB |
| 4330736 | 1764 | 41.99476 | 102.9992 | 1.294352 | 0.000105 | mTERF |
| 1.07E+08 | 2415 | 513.0794 | 27.90802 | -4.20043 | 2.46E-17 | AP2-EREBP |
| 4338741 | 1394 | 7.005525 | 25.78927 | 1.880206 | 0.000741 | mTERF |
| 4324418 | 3745 | 1345.36 | 534.7956 | -1.33093 | 5.79E-15 | AP2-EREBP |
| 9269072 | 2700 | 685.3079 | 1898.211 | 1.469816 | 2.63E-07 | AP2-EREBP |
| 4330027 | 1053 | 3684.929 | 371.7198 | -3.30935 | 1.44E-17 | MYB |
| 9269507 | 1582 | 52.56593 | 19.19318 | -1.45353 | 0.005309 | bHLH |
| 1.07E+08 | 973 | 18.08403 | 4.291686 | -2.0751 | 0.007265 | C2H2 |
| 4328809 | 1407 | 119.7174 | 420.5451 | 1.812628 | 1.26E-09 | HSF |
| 1.07E+08 | 2205 | 186.3306 | 32.41708 | -2.52304 | 1.50E-09 | GRAS |
| 4347711 | 1740 | 356.1376 | 846.3319 | 1.248789 | 1.93E-06 | MYB |
| 9270031 | 1446 | 136.338 | 30.76927 | -2.14763 | 4.45E-08 | WRKY |
| 4332111 | 2699 | 2284.081 | 1017.549 | -1.16652 | 8.23E-10 | ARR-B |
| 4327669 | 2043 | 2074.516 | 870.1909 | -1.25337 | 6.05E-07 | bHLH |
| 1.07E+08 | 1193 | 8.354713 | 1.283743 | -2.70223 | 0.006149 | WRKY |
| 1.07E+08 | 1955 | 6.453567 | 30.96766 | 2.262594 | 0.000202 | bHLH |
| 4330191 | 1492 | 149.4297 | 36.83127 | -2.02046 | 1.36E-10 | AP2-EREBP |
| 4330939 | 2030 | 39.2152 | 91.53875 | 1.22297 | 0.0002 | mTERF |
| 4347516 | 2285 | 5.978896 | 23.06794 | 1.947938 | 0.001514 | SBP |
| 1.07E+08 | 639 | 10.48114 | 1.489098 | -2.81529 | 0.002589 | bHLH |
| 1.07E+08 | 1435 | 3.714031 | 18.0248 | 2.278926 | 0.001163 | AP2-EREBP |
| 4329612 | 5700 | 1626.52 | 96.6634 | -4.07267 | 1.22E-27 | AP2-EREBP |
| 4330306 | 1266 | 4848.609 | 97.34909 | -5.63826 | 2.43E-98 | AP2-EREBP |
| 4342232 | 1343 | 8.556761 | 31.95685 | 1.900988 | 0.000848 | WRKY |
| 4324267 | 3455 | 66.01925 | 134.9689 | 1.031668 | 0.001872 | C2C2-Dof |
| 4335893 | 1698 | 13.95814 | 2.507806 | -2.47661 | 0.002075 | zf-HD |
| 4330203 | 1298 | 34.84331 | 152.4739 | 2.129609 | 2.47E-08 | AP2-EREBP |
| 4331520 | 1449 | 449.1916 | 199.5198 | -1.1708 | 2.26E-06 | NAC |
| 4345940 | 2636 | 13.88772 | 37.95479 | 1.450473 | 0.004941 | FAR1 |
| 4335878 | 1266 | 282.3919 | 41.41394 | -2.76951 | 3.85E-09 | GRAS |
| 4328362 | 1476 | 934.7001 | 445.8421 | -1.06797 | 0.000103 | AP2-EREBP |
| 1.07E+08 | 1590 | 69.19114 | 17.89443 | -1.95108 | 1.13E-05 | AP2-EREBP |
| 9272566 | 2228 | 71.49654 | 179.9029 | 1.331273 | 0.000115 | C2C2-Dof |
| 4330462 | 1682 | 128.3097 | 49.96775 | -1.36056 | 0.000807 | bHLH |
| 4351978 | 2075 | 1402.305 | 681.496 | -1.04102 | 5.73E-10 | C3H |
| 1.07E+08 | 3149 | 43.09165 | 14.99581 | -1.52285 | 0.000335 | WRKY |
| 9271266 | 1886 | 3.7696 | 31.90499 | 3.081299 | 2.15E-06 | AP2-EREBP |
| 4346229 | 1755 | 3933.765 | 767.1427 | -2.35834 | 7.75E-14 | HSF |
| 1.07E+08 | 1532 | 330.3991 | 52.43248 | -2.65568 | 9.22E-09 | AP2-EREBP |
| 4338484 | 1762 | 2397.617 | 674.9246 | -1.8288 | 7.38E-12 | AP2-EREBP |
| 4345625 | 2018 | 303.4365 | 2243.19 | 2.886086 | 2.42E-64 | Tify |
| 4332651 | 1754 | 5647.453 | 739.6377 | -2.93271 | 6.95E-47 | MYB |
| 4330231 | 2919 | 169.9518 | 411.2433 | 1.274866 | 2.31E-06 | GRAS |
| 4339409 | 2093 | 577.5224 | 188.1875 | -1.61771 | 1.25E-05 | HSF |
| 4331834 | 981 | 12953.29 | 2619.92 | -2.30572 | 1.05E-16 | Tify |
| 4352343 | 3037 | 187.398 | 454.0803 | 1.276842 | 1.26E-07 | C3H |
| 4338852 | 2556 | 8606.231 | 4085.021 | -1.07504 | 1.14E-08 | NAC |
| 4325972 | 1235 | 1265.587 | 2534.045 | 1.001636 | 9.87E-10 | LOB |
| 4350721 | 1970 | 93.48084 | 188.107 | 1.008811 | 0.00048 | BSD |
| 4324770 | 1133 | 3208.643 | 818.6107 | -1.97071 | 3.69E-20 | MYB |
| 4334944 | 1752 | 111.3054 | 338.9023 | 1.606346 | 2.64E-05 | C2H2 |
| 1.07E+08 | 732 | 39.84418 | 9.02225 | -2.14281 | 1.33E-05 | AP2-EREBP |
| 4324783 | 1186 | 7799.009 | 1021.723 | -2.93229 | 1.46E-24 | MYB |
| 4348526 | 1242 | 376.6104 | 1773.564 | 2.235506 | 3.50E-21 | AP2-EREBP |
| 4347069 | 1472 | 3777.649 | 88.37125 | -5.41777 | 7.59E-47 | WRKY |
| 4339902 | 1573 | 11.98621 | 38.2383 | 1.673643 | 0.010343 | MYB |
| 4337654 | 2422 | 685.2629 | 3368.793 | 2.297503 | 1.81E-30 | AP2-EREBP |
| 4342046 | 2011 | 101.3867 | 215.3204 | 1.086616 | 0.013251 | SRS |
| 4330950 | 1537 | 505.6337 | 244.3911 | -1.0489 | 0.000279 | MYB |
| 4327512 | 2012 | 359.6281 | 156.7071 | -1.19844 | 8.98E-07 | G2-like |
| 4328512 | 1630 | 30206.18 | 3843.598 | -2.97431 | 1.37E-25 | WRKY |
| 4351217 | 2301 | 766.5279 | 355.489 | -1.10853 | 1.25E-08 | GRAS |
| 4347252 | 1260 | 692.7765 | 243.1523 | -1.51053 | 0.000617 | bHLH |
| 4348490 | 1438 | 43.64539 | 433.9656 | 3.313679 | 1.33E-06 | bHLH |
| 9270361 | 3907 | 903.691 | 190.8924 | -2.24307 | 2.72E-20 | ARF |
| 4327694 | 2158 | 7.008767 | 52.40448 | 2.902458 | 0.000114 | C2H2 |
| 4332704 | 1626 | 353.3623 | 735.8716 | 1.058306 | 8.72E-05 | G2-like |
| 4325750 | 1926 | 48.98743 | 19.82127 | -1.30536 | 0.008985 | bHLH |
| 4348585 | 1905 | 501.2189 | 1287.741 | 1.36133 | 3.61E-08 | C2C2-Dof |
| 4351199 | 840 | 49.44161 | 3.806017 | -3.69937 | 6.02E-09 | C2H2 |
| 4342269 | 1586 | 86.39055 | 350.962 | 2.022369 | 8.72E-09 | G2-like |
| 1.07E+08 | 1640 | 10.43449 | 34.46205 | 1.723649 | 0.002329 | ABI3VP1 |
| 4340229 | 1960 | 2754.685 | 280.9276 | -3.29362 | 5.94E-37 | bHLH |
| 4330470 | 1852 | 223.6042 | 54.34798 | -2.04065 | 0.001261 | C2C2-Dof |
| 4348918 | 2225 | 246.1397 | 646.9239 | 1.394118 | 6.07E-12 | ARF |
| 4337718 | 1354 | 3094.561 | 300.6066 | -3.36379 | 7.90E-17 | MYB |
| 4324426 | 1747 | 2448.507 | 545.4763 | -2.16631 | 1.38E-16 | WRKY |
| 4346351 | 1559 | 5.631088 | 0.58528 | -3.26621 | 0.001812 | AP2-EREBP |
| 9270770 | 2007 | 93.21539 | 355.3469 | 1.930588 | 1.97E-06 | bHLH |
| 4346047 | 2572 | 9.562353 | 52.52246 | 2.457497 | 2.77E-07 | mTERF |
| 4345673 | 1798 | 35.44919 | 96.61292 | 1.446464 | 1.92E-05 | zf-HD |
| 1.07E+08 | 1002 | 897.1551 | 63.57342 | -3.81886 | 1.04E-38 | AP2-EREBP |
| 4338909 | 2229 | 2135.92 | 595.048 | -1.84378 | 6.12E-08 | TUB |
| 4352653 | 1323 | 2737.937 | 314.4522 | -3.12218 | 7.20E-30 | C2H2 |
| 4351545 | 1273 | 724.469 | 20.5313 | -5.14103 | 2.75E-28 | NAC |
| 1.07E+08 | 3401 | 40.36579 | 99.66571 | 1.303964 | 0.000177 | FAR1 |
| 4325059 | 1855 | 1995.418 | 252.153 | -2.98432 | 9.18E-15 | C2H2 |
| 4339739 | 1562 | 1466.676 | 666.463 | -1.13795 | 0.001756 | WRKY |
| 4327914 | 2004 | 17.16301 | 73.46557 | 2.097765 | 0.000297 | MYB |
| 4348531 | 840 | 5783.863 | 251.4259 | -4.52383 | 1.14E-37 | Tify |
| 4337480 | 1849 | 252.6928 | 805.1858 | 1.671937 | 8.98E-11 | TUB |
| 4339005 | 2248 | 342.3297 | 137.2113 | -1.31899 | 3.27E-07 | MYB |
| 1.07E+08 | 2632 | 217.5966 | 16.08463 | -3.7579 | 2.74E-12 | MYB |
| 1.07E+08 | 1843 | 56.5795 | 13.5295 | -2.06417 | 2.96E-05 | EIL |
| 4336551 | 1108 | 16445.94 | 3473.293 | -2.24336 | 4.85E-41 | Trihelix |
| 4347486 | 1618 | 499.9679 | 227.8571 | -1.13371 | 0.001055 | bHLH |
| 4325992 | 1562 | 34.70827 | 77.40248 | 1.1571 | 0.01062 | MYB |
| 4327611 | 2050 | 2728.89 | 194.3785 | -3.81137 | 6.97E-32 | WRKY |
| 4325137 | 1650 | 4.102269 | 0.558506 | -2.87678 | 0.006823 | TCP |
| 4331028 | 1686 | 848.1394 | 291.1408 | -1.54258 | 7.81E-06 | DBP |
| 4334181 | 1969 | 13.9017 | 69.67351 | 2.325349 | 0.001492 | bHLH |
| 4343296 | 3142 | 109.4002 | 251.0066 | 1.19811 | 3.84E-07 | FAR1 |
| 4337852 | 1208 | 36.26026 | 131.0617 | 1.853785 | 2.39E-05 | C2C2-GATA |
| 4345697 | 1870 | 40.08706 | 106.0992 | 1.404206 | 8.79E-05 | AP2-EREBP |
| 4324327 | 2379 | 251.6097 | 549.5427 | 1.127044 | 5.95E-08 | bHLH |
| 4351611 | 1811 | 249.3707 | 530.3507 | 1.088655 | 9.79E-06 | C2C2-GATA |
| 4326315 | 2074 | 859.3699 | 287.572 | -1.57936 | 9.75E-09 | bHLH |
| 4340188 | 3182 | 842.4423 | 28.06251 | -4.90786 | 1.37E-07 | WRKY |
| 4348646 | 2438 | 6383.821 | 788.5455 | -3.01715 | 2.83E-26 | HSF |
| 4330431 | 1550 | 375.1153 | 72.80969 | -2.36513 | 1.09E-08 | G2-like |
| 4333457 | 4716 | 1.211339 | 20.44097 | 4.076789 | 7.53E-06 | bHLH |
| 4334170 | 1608 | 2371.883 | 817.7324 | -1.53633 | 2.30E-08 | WRKY |
| 4327305 | 1673 | 189.8609 | 629.4049 | 1.729045 | 3.45E-06 | MYB |
| 9272089 | 2205 | 378.6525 | 159.7957 | -1.24465 | 7.58E-05 | ABI3VP1 |
| 1.07E+08 | 2318 | 296.6101 | 46.74694 | -2.66562 | 2.80E-16 | GRAS |
| 4348723 | 1579 | 47.78581 | 97.83283 | 1.033736 | 0.000594 | E2F-DP |
| 4339599 | 2019 | 13343.9 | 1414.758 | -3.23755 | 1.57E-40 | Trihelix |
| 4350036 | 1458 | 163.3353 | 333.4149 | 1.029482 | 0.005095 | MYB |
| 4326323 | 1596 | 136.2267 | 61.0163 | -1.15874 | 3.80E-05 | C3H |
| 9272517 | 961 | 6119.46 | 348.0068 | -4.13622 | 2.01E-29 | C2H2 |
| 4345807 | 1606 | 915.5681 | 369.1213 | -1.31057 | 2.71E-12 | bZIP |
| 4348906 | 1454 | 523.3572 | 54.54516 | -3.26227 | 3.90E-09 | MYB |
| 4352708 | 2082 | 804.2978 | 1760.862 | 1.13048 | 7.54E-07 | WRKY |
| 4329950 | 1671 | 102.4351 | 423.6053 | 2.048011 | 3.90E-14 | C2C2-CO-like |
| 4340746 | 1676 | 102.603 | 44.59449 | -1.20214 | 0.000526 | C2C2-CO-like |
| 4324664 | 1274 | 1132.586 | 131.5306 | -3.10615 | 4.48E-08 | MYB |
| 4343521 | 2155 | 23826.76 | 878.15 | -4.76197 | 2.21E-48 | GRAS |
| 4330189 | 1301 | 5408.195 | 1809.834 | -1.57929 | 3.55E-09 | AP2-EREBP |
| 4349649 | 4077 | 61.96671 | 6.937297 | -3.15905 | 1.07E-09 | NAC |
| 4336947 | 1145 | 24234.12 | 5798.906 | -2.06319 | 1.40E-37 | AP2-EREBP |
| 4339209 | 1158 | 11384.62 | 4267.38 | -1.41566 | 4.67E-26 | AP2-EREBP |
| 9267434 | 1770 | 267.422 | 57.68111 | -2.21295 | 1.12E-09 | NAC |
| 4351576 | 1413 | 876.8891 | 431.4846 | -1.02309 | 1.62E-08 | bZIP |
| 4343735 | 2297 | 17785.47 | 1457.796 | -3.60884 | 3.01E-42 | GRAS |
| 1.07E+08 | 1594 | 22.53711 | 45.38689 | 1.009973 | 0.012514 | GRAS |
| 4351184 | 1349 | 291.4627 | 931.9708 | 1.676974 | 9.10E-07 | MYB |
| 1.07E+08 | 1540 | 47.63075 | 23.11636 | -1.04298 | 0.012654 | WRKY |
| 4347637 | 2290 | 10665.59 | 2559.882 | -2.05882 | 8.87E-19 | HSF |
| 4339778 | 2313 | 97.6176 | 316.0276 | 1.694837 | 9.01E-08 | Sigma70-like |
| 4334060 | 2681 | 27862.95 | 1303.006 | -4.41843 | 4.59E-88 | bHLH |
| 4347431 | 2132 | 324.2768 | 716.969 | 1.144685 | 1.91E-05 | bHLH |
| 4343245 | 2007 | 865.481 | 242.713 | -1.83425 | 3.34E-13 | MYB |
| 4352838 | 1433 | 41.16934 | 104.1317 | 1.338767 | 0.000373 | C2C2-GATA |
| 4329526 | 2512 | 19457.75 | 929.259 | -4.38812 | 4.46E-19 | HSF |
| 9270355 | 1352 | 10.89526 | 39.61967 | 1.862516 | 0.0091 | SBP |
| 4350492 | 1991 | 1368.009 | 606.4898 | -1.17352 | 6.43E-13 | C3H |
| 4331707 | 1802 | 969.0944 | 413.0083 | -1.23047 | 3.93E-06 | HSF |
| 1.07E+08 | 2442 | 129.0651 | 61.31819 | -1.07371 | 0.008715 | WRKY |
| 4344814 | 2000 | 1.156576 | 19.39891 | 4.068043 | 4.83E-06 | MYB |
| 9269429 | 1237 | 7.666891 | 33.61569 | 2.132421 | 0.01226 | OFP |
| 4339348 | 1783 | 70.08253 | 182.9998 | 1.384715 | 7.84E-05 | C2C2-GATA |
| 4349938 | 1450 | 33.06372 | 135.4894 | 2.034859 | 0.000987 | MYB |
| 9268165 | 3125 | 5.093845 | 16.11677 | 1.661736 | 0.01217 | mTERF |
| 4326686 | 1488 | 73.317 | 24.92801 | -1.55638 | 0.00815 | OFP |
| 4338978 | 1328 | 8274.819 | 1571.97 | -2.39615 | 6.15E-15 | bHLH |
| 9268901 | 1562 | 37.87357 | 5.145309 | -2.87986 | 6.05E-05 | zf-HD |

**Table S7** A detailed list of TFs expressed differentially in CKSJ vs.D15SJ under low -T_w_ treatment.

| GeneID | Length | CKSJ-Expression | D15SJ-Expression | log2FoldChange(D15SJ/CKSJ) | Pvalue | Transcripts | TF_family |
| --- | --- | --- | --- | --- | --- | --- | --- |
| 4346629 | 1635 | 120.2305 | 743.0867 | 2.627728 | 1.12E-11 | XM_015755149.1 | AP2-EREBP |
| 9270656 | 1385 | 752.4976 | 327.8158 | -1.1988 | 0.000158 | XM_015768003.1 | C3H |
| 4326683 | 1454 | 222.3399 | 31.24865 | -2.8309 | 5.59E-20 | XM_015785114.1 | WRKY |
| 4341803 | 1603 | 639.5829 | 167.2764 | -1.9349 | 3.34E-10 | XM_015785966.1 | G2-like |
| 4329852 | 1938 | 175.7026 | 25.06694 | -2.80928 | 5.92E-11 | XM_015768600.1 | NAC |
| 4327245 | 1794 | 52.89409 | 180.3993 | 1.770015 | 2.09E-06 | XM_015755872.1 | SBP |
| 4345867 | 1564 | 5.83926 | 0.853737 | -2.77392 | 0.01192 | XM_015793927.1 | bHLH |
| 4341882 | 2904 | 162.4321 | 585.9196 | 1.850866 | 4.23E-12 | XM_015788652.1 | ARF |
| 4336779 | 2016 | 76.38389 | 191.5894 | 1.326677 | 3.13E-06 | XM_015780798.1 | MYB |
| 4344705 | 2926 | 8642.791 | 1948.581 | -2.14907 | 4.68E-23 | XM_015794803.1 | NAC |
| 4341994 | 1938 | 76.96657 | 182.8913 | 1.248682 | 0.000454 | BGI_novel_T007780 | C3H |
| 4324958 | 1680 | 1054.113 | 411.4426 | -1.35727 | 1.28E-09 | BGI_novel_T001595 | TAZ |
| 4341678 | 1652 | 2885.122 | 664.0358 | -2.1193 | 0.000108 | XM_015786092.1 | WRKY |
| 4334444 | 1216 | 1030.267 | 258.7333 | -1.99348 | 2.04E-14 | XM_015777362.1 | Alfin-like |
| 1.07E+08 | 1584 | 33.09083 | 103.4118 | 1.643897 | 6.42E-06 | XM_015762464.1 | SBP |
| 4335698 | 977 | 337.4654 | 68.64394 | -2.29754 | 9.92E-05 | XM_015778772.1 | Tify |
| 1.07E+08 | 2126 | 1.246798 | 11.55024 | 3.211623 | 0.003346 | XM_015789852.1 | C2H2 |
| 4331833 | 1141 | 12147.45 | 1925.64 | -2.65724 | 2.29E-06 | XM_015777660.1 | Tify |
| 4334553 | 1345 | 33151.68 | 5975.558 | -2.47194 | 2.33E-09 | XM_015775072.1 | NAC |
| 4337752 | 1885 | 17.04694 | 77.94612 | 2.192964 | 1.34E-05 | XM_015782796.1 | bHLH |
| 4328564 | 4017 | 270.4036 | 132.2939 | -1.03137 | 6.55E-05 | XM_015771701.1 | AP2-EREBP |
| 1.07E+08 | 1486 | 2280.864 | 81.08343 | -4.81403 | 7.95E-13 | XM_015769449.1 | AP2-EREBP |
| 4330864 | 2262 | 928.4179 | 294.5155 | -1.65643 | 0.000235 | XM_015768451.1 | WRKY |
| 4340749 | 2464 | 39.41357 | 124.859 | 1.663536 | 1.16E-05 | XM_015788048.1 | bHLH |
| 4333948 | 2528 | 1191.539 | 234.9196 | -2.34259 | 8.77E-19 | XM_015773363.1 | GRAS |
| 4339759 | 2181 | 7.638621 | 1.065508 | -2.84177 | 0.010879 | XM_015782230.1 | Sigma70-like |
| 4334592 | 909 | 18995.43 | 1800.353 | -3.3993 | 2.50E-11 | BGI_novel_T004734 | C2H2 |
| 4341986 | 2305 | 92.48042 | 386.6449 | 2.063789 | 2.01E-09 | XM_015787975.1 | SBP |
| 4347359 | 2207 | 1275.465 | 548.5055 | -1.21745 | 5.95E-06 | XM_015757038.1 | bHLH |
| 1.07E+08 | 1575 | 123.3866 | 260.8477 | 1.080022 | 0.001465 | XM_015771857.1 | bHLH |
| 1.07E+08 | 1401 | 749.7509 | 1743.014 | 1.2171 | 0.000615 | XM_015755469.1 | MYB |
| 4328080 | 1328 | 883.3343 | 378.8866 | -1.22119 | 1.13E-11 | XM_015770880.1 | bHLH |
| 1.07E+08 | 2749 | 568.88 | 26.34068 | -4.43276 | 4.99E-16 | XM_015776320.1 | bHLH |
| 4335707 | 3113 | 1219.751 | 245.553 | -2.31248 | 6.22E-06 | XM_015781117.1 | AP2-EREBP |
| 1.07E+08 | 1642 | 17.32101 | 44.50702 | 1.36151 | 0.009499 | XM_015765335.1 | bZIP |
| 4350864 | 1471 | 448.6307 | 124.4147 | -1.85037 | 9.23E-09 | BGI_novel_T011669 | bHLH |
| 4344312 | 3440 | 399.1532 | 859.4914 | 1.10654 | 0.000214 | XM_015791601.1 | C3H |
| 4349217 | 1123 | 284.4582 | 54.27708 | -2.3898 | 2.92E-07 | XM_015757946.1 | OFP |
| 4326726 | 1139 | 3511.011 | 1203.592 | -1.54454 | 0.001129 | XM_015775332.1 | MYB |
| 9271635 | 2133 | 54.61778 | 3.340341 | -4.0313 | 2.47E-06 | XM_015766399.1 | NOZZLE |
| 4351370 | 1753 | 535.6359 | 67.17172 | -2.99533 | 1.11E-22 | XM_015765044.1 | NAC |
| 1.07E+08 | 1903 | 184.0772 | 503.9246 | 1.452897 | 2.21E-05 | XM_015782638.1 | WRKY |
| 4339665 | 1123 | 296.5012 | 36.3033 | -3.02987 | 5.03E-12 | XM_015782927.1 | WRKY |
| 4347983 | 1553 | 33.58894 | 146.7032 | 2.126842 | 0.012141 | XM_015757239.1 | BBR/BPC |
| 4341990 | 1951 | 28.36607 | 107.7264 | 1.925134 | 3.00E-06 | XM_015788665.1 | G2-like |
| 4336585 | 1186 | 98.53476 | 233.5393 | 1.244961 | 0.004063 | XM_015780276.1 | AP2-EREBP |
| 4342538 | 3368 | 3325.501 | 1023.295 | -1.70035 | 2.31E-13 | BGI_novel_T008048 | CPP |
| 4350143 | 2448 | 301.9338 | 38.57536 | -2.96848 | 4.91E-12 | XM_015762406.1 | Alfin-like |
| 4328461 | 1539 | 83.94242 | 258.4961 | 1.62267 | 7.76E-09 | XM_015767591.1 | SBP |
| 4334216 | 1737 | 1410.538 | 505.528 | -1.48038 | 8.06E-05 | XM_015775720.1 | G2-like |
| 1.07E+08 | 1237 | 126.6885 | 335.9284 | 1.406868 | 0.009854 | XM_015783433.1 | WRKY |
| 4351577 | 2904 | 362.1789 | 155.1123 | -1.22339 | 1.58E-06 | XM_015763664.1 | GRAS |
| 9268771 | 2220 | 1972.696 | 348.5553 | -2.50071 | 1.45E-11 | XM_015772133.1 | AP2-EREBP |
| 1.07E+08 | 5640 | 1.011705 | 8.267981 | 3.030746 | 0.005803 | BGI_novel_T002179 | GRAS |
| 4332113 | 1723 | 2227.447 | 349.8235 | -2.67069 | 1.18E-23 | XM_015777855.1 | HSF |
| 4332755 | 1898 | 1333.466 | 331.8296 | -2.00667 | 2.08E-05 | BGI_novel_T004051 | WRKY |
| 4349650 | 1704 | 196.2791 | 35.73325 | -2.45757 | 3.05E-09 | XM_015760382.1 | NAC |
| 4331887 | 1314 | 512.0808 | 124.0198 | -2.0458 | 3.98E-06 | XM_015773787.1 | bHLH |
| 4352211 | 3296 | 217.1461 | 473.7647 | 1.125505 | 0.000443 | XM_015764926.1 | ARF |
| 9269465 | 1025 | 8469.837 | 203.3941 | -5.37998 | 5.57E-31 | XM_015779571.1 | bHLH |
| 1.07E+08 | 1655 | 122.5732 | 383.2509 | 1.644646 | 0.000191 | XM_015791028.1 | NAC |
| 4342759 | 1465 | 1470.96 | 577.7998 | -1.34812 | 0.000386 | XM_015789877.1 | AP2-EREBP |
| 4327014 | 3006 | 68.4126 | 160.675 | 1.231812 | 0.000859 | XM_015779017.1 | ARF |
| 4342383 | 2337 | 12.95357 | 111.2607 | 3.102523 | 1.59E-06 | BGI_novel_T007974 | bHLH |
| 1.07E+08 | 1694 | 508.1809 | 77.8425 | -2.70671 | 3.82E-13 | XM_015760947.1 | WRKY |
| 4336288 | 1584 | 246.8783 | 88.40586 | -1.48159 | 9.58E-08 | XM_015778355.1 | C2C2-CO-like |
| 4330293 | 2577 | 4091.705 | 1976.709 | -1.0496 | 1.87E-05 | XM_015772065.1 | C2C2-Dof |
| 4333315 | 1819 | 14.23392 | 45.47684 | 1.675799 | 0.000984 | XM_015773879.1 | MYB |
| 4333065 | 1251 | 5169.221 | 1323.717 | -1.96535 | 1.24E-11 | XM_015775146.1 | Tify |
| 4325479 | 1557 | 524.9366 | 179.3566 | -1.54931 | 0.000194 | XM_015766773.1 | AP2-EREBP |
| 4344331 | 2405 | 4695.234 | 1263.63 | -1.89362 | 2.87E-12 | XM_015791088.1 | EIL |
| 9267785 | 1633 | 168.8958 | 37.3569 | -2.17669 | 0.004727 | BGI_novel_T006541 | AP2-EREBP |
| 4343868 | 1256 | 1156.586 | 416.1612 | -1.47466 | 2.73E-11 | XM_015791628.1 | Alfin-like |
| 9267508 | 1158 | 1741.775 | 70.91471 | -4.61833 | 6.49E-72 | XM_015783322.1 | WRKY |
| 4330143 | 2068 | 208.8286 | 520.5388 | 1.317686 | 3.77E-05 | XM_015767871.1 | C2C2-GATA |
| 4347070 | 1248 | 793.2433 | 83.54046 | -3.24722 | 2.21E-15 | XM_015755269.1 | WRKY |
| 4349814 | 1393 | 92.32812 | 13.52817 | -2.7708 | 8.21E-06 | BGI_novel_T011145 | NAC |
| 4340303 | 1901 | 115.7425 | 257.3318 | 1.152712 | 4.32E-05 | XM_015787899.1 | DBP |
| 1.07E+08 | 1138 | 75.12956 | 15.98032 | -2.23308 | 0.003883 | XM_015783418.1 | WRKY |
| 4330722 | 2390 | 22.03907 | 88.56515 | 2.006676 | 0.000903 | XM_015768530.1 | TCP |
| 4339490 | 1535 | 70.92821 | 178.0078 | 1.327509 | 0.006011 | XM_015782699.1 | MYB |
| 4337692 | 2332 | 23746.75 | 10554.68 | -1.16985 | 1.17E-05 | BGI_novel_T006052 | C3H |
| 9270349 | 2121 | 35304.06 | 1357.388 | -4.70093 | 1.04E-25 | BGI_novel_T002929 | AP2-EREBP |
| 4327325 | 1748 | 13.95866 | 126.7385 | 3.182623 | 9.70E-09 | XM_015771425.1 | bHLH |
| 4348485 | 3350 | 2754.874 | 1265.887 | -1.12184 | 2.00E-09 | XM_015758912.1 | TIG |
| 9266609 | 1513 | 71.39414 | 196.2335 | 1.458694 | 1.39E-05 | XM_015785989.1 | mTERF |
| 4342999 | 2806 | 14.91308 | 76.27597 | 2.35465 | 1.03E-06 | XM_015789537.1 | mTERF |
| 4344293 | 3446 | 251.0063 | 972.7662 | 1.954369 | 9.19E-10 | XM_015791292.1 | ABI3VP1 |
| BGI_novel_G000419 | 2277 | 84.01728 | 7.842213 | -3.42135 | 1.45E-09 | BGI_novel_T006821 | AP2-EREBP |
| 4333934 | 1482 | 97.84871 | 347.8857 | 1.829989 | 4.56E-07 | XM_015773452.1 | MYB |
| 4340004 | 1642 | 84.65773 | 214.0141 | 1.337992 | 1.65E-05 | XM_015785668.1 | BBR/BPC |
| 4341999 | 5662 | 571.031 | 209.1132 | -1.44928 | 2.90E-05 | XM_015786946.1 | TAZ |
| 4324801 | 1560 | 5.561364 | 26.03275 | 2.226817 | 0.009149 | XM_015775202.1 | MYB |
| 4327319 | 2551 | 342.8676 | 891.2227 | 1.378134 | 3.30E-10 | XM_015767005.1 | C2H2 |
| 4352661 | 1606 | 105.4804 | 351.2506 | 1.735526 | 0.000213 | XM_015763301.1 | G2-like |
| 4343653 | 1367 | 1017.152 | 145.6932 | -2.80353 | 1.41E-47 | XM_015789406.1 | C2H2 |
| 4326230 | 2355 | 638.2239 | 212.261 | -1.58822 | 3.16E-11 | XM_015766943.1 | C3H |
| 4348439 | 2185 | 3445.684 | 928.1192 | -1.89241 | 6.03E-29 | XM_015759061.1 | NAC |
| 4338934 | 1821 | 3352.586 | 532.227 | -2.65516 | 6.99E-11 | XM_015785258.1 | C2H2 |
| 4347142 | 1631 | 11341.22 | 25918.8 | 1.192424 | 0.003543 | XM_015756387.1 | AP2-EREBP |
| 4341753 | 1463 | 840.8052 | 248.6483 | -1.75767 | 1.01E-08 | XM_015788319.1 | bZIP |
| 4339928 | 1583 | 11.08036 | 60.47952 | 2.448442 | 4.45E-05 | XR_001546469.1 | GRF |
| 1.07E+08 | 1543 | 3.237356 | 17.35376 | 2.422361 | 0.008108 | XM_015774050.1 | GRAS |
| 4336519 | 1348 | 178.0674 | 44.08449 | -2.01408 | 0.004881 | XM_015779109.1 | NAC |
| 4343764 | 1068 | 21.39338 | 96.62726 | 2.175266 | 1.27E-05 | XM_015791723.1 | C2H2 |
| 4332331 | 1102 | 92.86929 | 22.89755 | -2.02001 | 0.000245 | XM_015774664.1 | AP2-EREBP |
| 4342790 | 1741 | 3.765781 | 17.31042 | 2.200619 | 0.010671 | XM_015790054.1 | C2C2-Dof |
| 4342421 | 1158 | 407.0341 | 76.65425 | -2.40871 | 8.91E-05 | XM_015790756.1 | Tify |
| 9271013 | 2710 | 18.36339 | 53.33923 | 1.538365 | 0.003653 | XM_015760058.1 | FAR1 |
| 1.07E+08 | 4217 | 24.18803 | 71.59288 | 1.565523 | 0.005358 | BGI_novel_T010293 | mTERF |
| 4331600 | 1538 | 188.6349 | 93.51261 | -1.01236 | 0.012238 | XM_015773734.1 | C2C2-GATA |
| 4332403 | 2874 | 5049.555 | 1136.274 | -2.15185 | 3.54E-16 | XM_015776359.1 | MYB |
| 4330829 | 1187 | 18091.05 | 766.5624 | -4.56073 | 4.21E-22 | XM_015768788.1 | AP2-EREBP |
| 9272478 | 2179 | 1769.321 | 859.6378 | -1.04139 | 0.000268 | XM_015755299.1 | bHLH |
| 4328265 | 4223 | 27.65011 | 83.55785 | 1.59549 | 4.96E-05 | BGI_novel_T002007 | ARF |
| 4325454 | 2089 | 443.9674 | 956.5084 | 1.107324 | 5.12E-09 | XM_015766331.1 | MYB |
| 4336052 | 1466 | 402.2325 | 1125.692 | 1.484711 | 6.97E-09 | XM_015778438.1 | NAC |
| 4341326 | 1873 | 182.4826 | 13.17825 | -3.79153 | 1.87E-17 | BGI_novel_T007509 | HSF |
| 4329309 | 1101 | 1856.496 | 144.4675 | -3.68377 | 3.32E-11 | XM_015769356.1 | WRKY |
| 4345899 | 2086 | 1863.684 | 567.3915 | -1.71574 | 6.40E-07 | XM_015795004.1 | bHLH |
| 9268190 | 1509 | 2079.816 | 122.3647 | -4.0872 | 1.45E-14 | XM_015776174.1 | MYB |
| 4327270 | 1572 | 898.2922 | 3762.527 | 2.066445 | 1.06E-11 | XM_015782036.1 | Trihelix |
| 9266067 | 1829 | 113.1195 | 8.389719 | -3.75308 | 2.71E-08 | XM_015783257.1 | WRKY |
| 4336759 | 2440 | 2349.883 | 1013.201 | -1.21367 | 5.90E-08 | XM_015781614.1 | GRAS |
| 4330397 | 1834 | 61.53548 | 133.6833 | 1.119329 | 0.010776 | XM_015768130.1 | MYB |
| 9268836 | 2327 | 1059.031 | 73.64661 | -3.84598 | 2.51E-14 | XM_015765745.1 | WRKY |
| 4344289 | 1339 | 2.461436 | 19.25902 | 2.967962 | 0.000976 | XM_015791287.1 | OFP |
| 4334219 | 1679 | 13.05562 | 2.153464 | -2.59994 | 0.004099 | XM_015773932.1 | C2C2-Dof |
| 4340317 | 2125 | 953.4536 | 226.2527 | -2.07523 | 1.17E-17 | XM_015788597.1 | LIM |
| 4338474 | 2090 | 17178.43 | 7947.588 | -1.11201 | 0.000197 | XM_015784890.1 | WRKY |
| 4330230 | 2917 | 127.9841 | 282.9929 | 1.144802 | 9.11E-07 | XM_015771930.1 | GRAS |
| 4333464 | 4240 | 78.42024 | 195.0058 | 1.314219 | 2.18E-05 | XM_015777563.1 | ABI3VP1 |
| 4346023 | 4563 | 5683.603 | 2510.717 | -1.17871 | 1.12E-10 | XM_015794435.1 | SBP |
| 4340382 | 2006 | 2382.555 | 807.3084 | -1.56132 | 2.26E-14 | BGI_novel_T007105 | bHLH |
| 4338039 | 3667 | 102.5408 | 428.447 | 2.062919 | 1.08E-05 | BGI_novel_T006198 | MYB |
| 4342753 | 1330 | 3101.182 | 727.1522 | -2.09249 | 9.97E-06 | XM_015789542.1 | NAC |
| 4347266 | 1087 | 7050.4 | 1466.698 | -2.26513 | 7.42E-05 | XM_015755558.1 | AP2-EREBP |
| 4338032 | 1519 | 802.8452 | 76.34729 | -3.39447 | 3.18E-08 | XM_015782464.1 | NAC |
| 1.07E+08 | 1262 | 19.1833 | 1.442323 | -3.73338 | 5.30E-05 | XM_015783410.1 | NAC |
| 4339092 | 2284 | 2610.726 | 694.8101 | -1.90976 | 0.004809 | XM_015782350.1 | WRKY |
| 4352559 | 1200 | 225.7676 | 1553.685 | 2.782784 | 4.25E-16 | XM_015764741.1 | MYB |
| 4337905 | 1220 | 103.6107 | 12.45899 | -3.05591 | 3.04E-05 | XM_015782893.1 | bHLH |
| 4334471 | 1283 | 50.92714 | 10.36404 | -2.29685 | 2.24E-06 | XM_015773837.1 | bHLH |
| 4331412 | 713 | 26.58396 | 4.815209 | -2.46489 | 0.001028 | XM_015773950.1 | AP2-EREBP |
| 4325120 | 2005 | 7991.222 | 1048.534 | -2.93004 | 8.27E-22 | XM_015794298.1 | NAC |
| 4348481 | 2991 | 449.4456 | 44.71229 | -3.3294 | 1.79E-06 | XM_015757361.1 | NAC |
| 9271723 | 863 | 54.07945 | 224.5681 | 2.054 | 2.06E-07 | XM_015768713.1 | ABI3VP1 |
| 4329674 | 2518 | 156.5124 | 346.1676 | 1.145194 | 1.91E-05 | BGI_novel_T002682 | C3H |
| 4349881 | 1373 | 4700.73 | 1096.434 | -2.10007 | 0.001571 | XM_015762132.1 | AP2-EREBP |
| 9272091 | 1120 | 21.49771 | 94.30056 | 2.133083 | 0.000359 | XM_015763436.1 | MYB |
| 9269795 | 1573 | 64.4619 | 180.3224 | 1.48406 | 0.000829 | XM_015785723.1 | C2C2-CO-like |
| 4343630 | 2285 | 204.9631 | 9.542793 | -4.42481 | 1.39E-11 | XM_015790191.1 | NAC |
| 4335808 | 2096 | 502.1462 | 105.3684 | -2.25266 | 1.31E-12 | BGI_novel_T005292 | E2F-DP |
| 4334294 | 1545 | 76.30076 | 273.409 | 1.841291 | 0.001616 | XM_015773360.1 | NAC |
| 4333157 | 2458 | 2988.89 | 1120.9 | -1.41495 | 1.89E-11 | XM_015776409.1 | MYB |
| 4330935 | 1667 | 7909.767 | 3055.41 | -1.37227 | 8.59E-09 | XM_015769977.1 | AP2-EREBP |
| 4339597 | 2186 | 387.5473 | 180.195 | -1.10481 | 0.013076 | BGI_novel_T006808 | TUB |
| 4339875 | 1025 | 70.3814 | 156.3983 | 1.151958 | 0.012533 | XM_015787048.1 | ABI3VP1 |
| 4337494 | 1933 | 7.605914 | 69.81595 | 3.198363 | 7.98E-08 | XM_015778794.1 | C2H2 |
| 1.07E+08 | 858 | 907.7185 | 68.52521 | -3.72754 | 9.79E-14 | XM_015790485.1 | AP2-EREBP |
| 4331648 | 1759 | 1571.662 | 167.7528 | -3.22788 | 1.12E-27 | BGI_novel_T003538 | E2F-DP |
| 4326293 | 1415 | 116.3522 | 236.5229 | 1.023482 | 0.002058 | XM_015765518.1 | NAC |
| 4343641 | 2665 | 2516.107 | 1230.445 | -1.03201 | 6.29E-05 | XM_015792021.1 | C3H |
| 4328230 | 2572 | 62.91724 | 22.19436 | -1.50326 | 0.013913 | XM_015772224.1 | RWP-RK |
| 4324850 | 2218 | 3983.677 | 1636.907 | -1.28313 | 1.82E-15 | XM_015755342.1 | GRAS |
| 1.07E+08 | 1139 | 1.675503 | 10.59609 | 2.660866 | 0.008877 | XM_015790228.1 | MYB |
| 4339522 | 1756 | 1334.913 | 630.8224 | -1.08144 | 0.006158 | XM_015782331.1 | AP2-EREBP |
| 4325718 | 1931 | 99.53318 | 21.17343 | -2.23292 | 9.18E-08 | XM_015768824.1 | MYB |
| 9268032 | 3492 | 260.5089 | 552.7102 | 1.08519 | 1.15E-05 | XM_015756629.1 | RWP-RK |
| 4339974 | 1096 | 25768.84 | 1754.303 | -3.87666 | 4.63E-16 | XM_015786965.1 | AP2-EREBP |
| 4335388 | 1740 | 4106.712 | 1913.742 | -1.10159 | 0.000176 | XM_015778086.1 | WRKY |
| 4324028 | 1504 | 911.5381 | 3185.528 | 1.805158 | 7.70E-16 | XM_015772758.1 | LOB |
| 4330202 | 1534 | 60.09781 | 389.3518 | 2.69569 | 2.47E-11 | XM_015770831.1 | AP2-EREBP |
| 4325657 | 1897 | 3158.072 | 983.4318 | -1.68315 | 0.000397 | XM_015769774.1 | WRKY |
| 4336689 | 3111 | 244.4721 | 495.4494 | 1.019068 | 3.19E-07 | BGI_novel_T005630 | C2H2 |
| 4340715 | 2030 | 677.5402 | 333.1209 | -1.02426 | 5.99E-09 | BGI_novel_T007239 | NAC |
| 1.07E+08 | 624 | 5.30107 | 0.56232 | -3.23682 | 0.004081 | XM_015786744.1 | AP2-EREBP |
| 4349997 | 1873 | 90.37499 | 180.9628 | 1.001697 | 0.011447 | XM_015759887.1 | ABI3VP1 |
| 4331949 | 2186 | 189.8137 | 584.0379 | 1.621478 | 5.54E-09 | XM_015777170.1 | C2H2 |
| 4339151 | 1408 | 5.009446 | 19.73899 | 1.978326 | 0.011884 | XM_015784931.1 | G2-like |
| 4331765 | 1967 | 308.9533 | 633.8097 | 1.036661 | 0.000115 | XM_015776308.1 | C2C2-Dof |
| 4327499 | 3060 | 2354.868 | 1027.323 | -1.19676 | 5.94E-09 | XM_015763813.1 | GRAS |
| 4345659 | 1794 | 446.4663 | 1034.618 | 1.212475 | 1.02E-06 | XM_015795367.1 | G2-like |
| 9270639 | 2497 | 20.33186 | 92.82373 | 2.190752 | 5.38E-05 | XM_015771392.1 | SBP |
| 4344514 | 1776 | 44.069 | 156.4736 | 1.828083 | 0.001893 | XM_015793488.1 | NAC |
| 4336872 | 1526 | 302.2639 | 30.75257 | -3.29703 | 1.92E-22 | XM_015779922.1 | G2-like |
| 4330001 | 1458 | 756.7337 | 294.5632 | -1.36121 | 5.50E-05 | XM_015768685.1 | MYB |
| 4328953 | 1959 | 1678.41 | 518.7999 | -1.69385 | 9.52E-08 | XM_015767914.1 | WRKY |
| 4336500 | 1676 | 35.08498 | 135.9349 | 1.953991 | 0.001781 | XM_015780748.1 | MYB |
| 4327261 | 1823 | 459.8621 | 1052.622 | 1.194714 | 5.01E-10 | XM_015766300.1 | TCP |
| 4334213 | 1261 | 28147.1 | 13178.05 | -1.09485 | 0.004632 | BGI_novel_T004589 | C2H2 |
| 4327362 | 2397 | 99.61663 | 202.3444 | 1.022355 | 9.17E-05 | XM_015766850.1 | MYB |
| 4339713 | 3989 | 28.03861 | 8.891265 | -1.65695 | 0.011148 | XM_015784071.1 | zf-HD |
| 9268095 | 1946 | 662.2052 | 111.8855 | -2.56526 | 4.29E-12 | XM_015782431.1 | WRKY |
| 4338832 | 1511 | 1023.08 | 121.528 | -3.07356 | 2.26E-21 | XM_015782002.1 | NAC |
| 4329629 | 2507 | 47.6926 | 156.2825 | 1.712319 | 2.08E-06 | XM_015770947.1 | FAR1 |
| BGI_novel_G000398 | 1156 | 218.6019 | 13.01829 | -4.06969 | 1.06E-13 | BGI_novel_T006557 | MYB |
| 4330466 | 3756 | 138.1156 | 7.773193 | -4.15123 | 2.68E-13 | XM_015771338.1 | zf-HD |
| 4326856 | 2230 | 2104.723 | 573.7318 | -1.87518 | 1.13E-06 | BGI_novel_T001023 | WRKY |
| 1.07E+08 | 1964 | 93.55367 | 14.7187 | -2.66814 | 5.59E-09 | XM_015764139.1 | GRAS |
| 4333810 | 3314 | 2875.739 | 1391.603 | -1.04719 | 2.14E-05 | XM_015776568.1 | C3H |
| 4326760 | 2285 | 11882.85 | 2222.751 | -2.41846 | 4.61E-10 | XM_015766655.1 | WRKY |
| 9266268 | 1098 | 84.43203 | 7.952379 | -3.40833 | 0.000122 | XM_015783468.1 | MYB |
| 4349118 | 4024 | 2123.679 | 327.5347 | -2.69685 | 1.17E-14 | BGI_novel_T010866 | Trihelix |
| 1.07E+08 | 2415 | 197.474 | 20.86851 | -3.24226 | 1.83E-22 | XM_015779546.1 | AP2-EREBP |
| 4324418 | 3745 | 1565.156 | 541.6354 | -1.53091 | 1.54E-14 | XM_015765853.1 | AP2-EREBP |
| 9269072 | 2705 | 1046.4 | 2316.014 | 1.14621 | 0.001028 | BGI_novel_T005865 | AP2-EREBP |
| 4330027 | 1053 | 4451.929 | 553.6269 | -3.00744 | 3.82E-08 | XM_015767638.1 | MYB |
| 4324153 | 1447 | 44.78377 | 13.52324 | -1.72754 | 0.010392 | XM_015786202.1 | OFP |
| 4327485 | 1465 | 2288.422 | 285.0607 | -3.00501 | 9.76E-08 | XM_015768220.1 | NAC |
| 4328809 | 1407 | 135.5437 | 303.2603 | 1.161799 | 2.67E-06 | XM_015768085.1 | HSF |
| 1.07E+08 | 2205 | 240.4658 | 28.09295 | -3.09755 | 1.22E-09 | XM_015761173.1 | GRAS |
| 4340770 | 1369 | 96.01956 | 276.5921 | 1.52636 | 5.45E-09 | BGI_novel_T007261 | C2C2-Dof |
| 4332111 | 2700 | 3501.845 | 1048.973 | -1.73914 | 5.03E-11 | XM_015775091.1 | ARR-B |
| 1.07E+08 | 1193 | 24.33838 | 1.05441 | -4.52872 | 6.92E-06 | XM_015786556.1 | WRKY |
| 4330191 | 1492 | 148.0557 | 40.24013 | -1.87943 | 7.14E-06 | XM_015768572.1 | AP2-EREBP |
| 4330939 | 2024 | 41.97207 | 108.6972 | 1.372814 | 0.00022 | XM_015767867.1 | mTERF |
| 4347516 | 2285 | 1.235989 | 26.31591 | 4.412197 | 1.31E-05 | XM_015755387.1 | SBP |
| 1.07E+08 | 639 | 19.2111 | 2.655832 | -2.8547 | 0.001687 | XM_015769602.1 | bHLH |
| 4329612 | 3338 | 1046.889 | 102.2204 | -3.35635 | 2.11E-09 | XM_015768980.1 | AP2-EREBP |
| 4330306 | 1266 | 2514.481 | 138.4053 | -4.18329 | 7.22E-14 | XM_015769271.1 | AP2-EREBP |
| 4324267 | 3455 | 40.94544 | 105.4192 | 1.364364 | 0.001152 | XM_015779748.1 | C2C2-Dof |
| 4335762 | 677 | 1789.877 | 655.733 | -1.44868 | 3.06E-08 | XM_015777930.1 | S1Fa-like |
| 4330203 | 1298 | 19.8604 | 160.9434 | 3.018587 | 3.86E-12 | XM_015768812.1 | AP2-EREBP |
| 4345940 | 2806 | 11.88053 | 46.51135 | 1.968984 | 0.000451 | XM_015794676.1 | FAR1 |
| 4335878 | 1266 | 192.6637 | 23.37463 | -3.04307 | 2.41E-07 | XM_015779353.1 | GRAS |
| 4328362 | 1476 | 967.2816 | 417.164 | -1.21332 | 9.75E-08 | XM_015768337.1 | AP2-EREBP |
| 4349484 | 2772 | 3932.955 | 1731.747 | -1.18339 | 1.37E-06 | XM_015758526.1 | bHLH |
| 9272566 | 2228 | 80.91249 | 224.3449 | 1.471284 | 2.63E-08 | XM_015762779.1 | C2C2-Dof |
| 4330462 | 2006 | 101.449 | 24.61587 | -2.04309 | 6.78E-06 | BGI_novel_T003023 | bHLH |
| 4351978 | 2078 | 1342.543 | 571.3845 | -1.23243 | 7.51E-13 | XM_015764816.1 | C3H |
| 4327165 | 1637 | 710.0028 | 1683.324 | 1.245417 | 6.20E-05 | XM_015794985.1 | bZIP |
| 4346229 | 1755 | 3185.087 | 827.8633 | -1.94387 | 3.89E-06 | BGI_novel_T009670 | HSF |
| 1.07E+08 | 1553 | 212.1877 | 65.03649 | -1.70602 | 6.94E-08 | XM_015770354.1 | AP2-EREBP |
| 4338484 | 1748 | 1466.618 | 447.4488 | -1.7127 | 2.74E-23 | XM_015784169.1 | AP2-EREBP |
| 4326351 | 1560 | 23.07279 | 5.886809 | -1.97064 | 0.009082 | XM_015776016.1 | HSF |
| 4345625 | 2018 | 448.9496 | 2346.868 | 2.386112 | 3.27E-44 | BGI_novel_T009415 | Tify |
| 4339196 | 1792 | 295.7934 | 641.5191 | 1.116903 | 8.82E-05 | XM_015783228.1 | bZIP |
| 4332651 | 1754 | 4732.519 | 1940.579 | -1.28612 | 6.83E-05 | XM_015772960.1 | MYB |
| 4334346 | 1864 | 17.39671 | 55.79147 | 1.68123 | 0.002823 | XM_015773598.1 | TCP |
| 4330231 | 2919 | 150.5977 | 391.4766 | 1.378226 | 4.98E-07 | XM_015771585.1 | GRAS |
| 4351386 | 1702 | 4.312573 | 18.91208 | 2.132687 | 0.007039 | XM_015764487.1 | AP2-EREBP |
| 4331834 | 981 | 12338.1 | 3198.935 | -1.94746 | 4.27E-05 | XM_015777661.1 | Tify |
| 4347679 | 4339 | 534.7153 | 1222.633 | 1.193148 | 4.00E-05 | XM_015756721.1 | MYB |
| 4352343 | 3027 | 203.7158 | 506.0911 | 1.312839 | 1.23E-07 | BGI_novel_T012358 | C3H |
| 4338852 | 2553 | 10862.39 | 4360.854 | -1.31666 | 6.93E-17 | XM_015783768.1 | NAC |
| 4325972 | 1235 | 1067.364 | 2468.767 | 1.209739 | 9.43E-09 | XM_015788984.1 | LOB |
| 4324770 | 1133 | 3012.507 | 1285.11 | -1.22907 | 4.32E-06 | XM_015772213.1 | MYB |
| 4334944 | 1752 | 62.00225 | 195.8453 | 1.659322 | 3.15E-05 | XM_015778813.1 | C2H2 |
| 4333920 | 3144 | 83.10245 | 200.474 | 1.270452 | 0.000301 | BGI_novel_T004502 | FAR1 |
| 4344703 | 2973 | 942.1179 | 1896.953 | 1.009705 | 0.002835 | BGI_novel_T009001 | MYB |
| 4324783 | 1186 | 4982.746 | 1047.72 | -2.24969 | 2.10E-12 | XM_015766974.1 | MYB |
| 1.07E+08 | 1518 | 5.733673 | 25.47254 | 2.151411 | 0.002903 | XM_015794061.1 | MADS |
| 4347530 | 1457 | 102.4886 | 242.0607 | 1.239905 | 0.003149 | XM_015795918.1 | NAC |
| 4348526 | 1242 | 340.0099 | 2368.64 | 2.80041 | 1.45E-17 | XM_015757876.1 | AP2-EREBP |
| 4347069 | 1472 | 1888.582 | 124.072 | -3.92805 | 8.42E-20 | XM_015755209.1 | WRKY |
| 4339902 | 1573 | 19.8409 | 65.1632 | 1.71558 | 0.013261 | XM_015786501.1 | MYB |
| 4337654 | 2422 | 812.1702 | 4302.998 | 2.405488 | 2.65E-36 | XM_015783844.1 | AP2-EREBP |
| 4330950 | 1537 | 588.3333 | 225.5597 | -1.38312 | 4.69E-05 | XM_015768701.1 | MYB |
| 4327512 | 1911 | 544.4043 | 182.1887 | -1.57925 | 1.45E-06 | XM_015755753.1 | G2-like |
| 4328512 | 1630 | 24995.69 | 5869.51 | -2.09037 | 2.23E-07 | XM_015771931.1 | WRKY |
| 4347252 | 1260 | 927.4865 | 128.7176 | -2.84912 | 9.25E-07 | XM_015755510.1 | bHLH |
| 4348490 | 1438 | 87.28954 | 296.4601 | 1.763957 | 0.00217 | XM_015757815.1 | bHLH |
| 4325838 | 1970 | 1209.962 | 3210.997 | 1.40806 | 3.54E-06 | XM_015755290.1 | GRAS |
| 4342550 | 1546 | 45.9194 | 95.23131 | 1.052332 | 0.013918 | BGI_novel_T008052 | HSF |
| 4327694 | 2158 | 5.21999 | 36.32295 | 2.798762 | 4.73E-05 | XM_015779463.1 | C2H2 |
| 4334257 | 2269 | 5.777778 | 0.589206 | -3.29367 | 0.003451 | XM_015774163.1 | AP2-EREBP |
| 4332704 | 1626 | 409.4062 | 862.1046 | 1.07433 | 0.004518 | XM_015772299.1 | G2-like |
| 4329901 | 1810 | 56.72643 | 131.2332 | 1.21004 | 0.000146 | XM_015770450.1 | mTERF |
| 4347439 | 2834 | 975.9937 | 365.0804 | -1.41866 | 1.78E-09 | XM_015756608.1 | EIL |
| 4351199 | 840 | 109.7034 | 25.74523 | -2.09123 | 2.22E-06 | XM_015761043.1 | C2H2 |
| 4340229 | 1982 | 2167.241 | 248.1334 | -3.12667 | 2.42E-31 | BGI_novel_T007036 | bHLH |
| 4324161 | 1326 | 287.376 | 91.5899 | -1.64968 | 0.000486 | XM_015765890.1 | WRKY |
| 4330470 | 1852 | 336.9924 | 33.10596 | -3.34755 | 1.08E-06 | XM_015767794.1 | C2C2-Dof |
| 4348918 | 2240 | 206.0942 | 576.6784 | 1.484463 | 2.73E-07 | XM_015759356.1 | ARF |
| 4349554 | 1780 | 88.04161 | 182.0661 | 1.048205 | 0.008886 | XM_015761629.1 | G2-like |
| 4337718 | 1354 | 2692.026 | 169.3983 | -3.9902 | 8.89E-16 | XM_015782974.1 | MYB |
| 4324426 | 1747 | 1625.401 | 646.7928 | -1.32942 | 0.000581 | XM_015769426.1 | WRKY |
| 9272222 | 2189 | 1698.326 | 5128.12 | 1.594316 | 1.39E-11 | BGI_novel_T000516 | WRKY |
| 9270770 | 2007 | 97.30374 | 248.7246 | 1.353982 | 7.75E-07 | XM_015760147.1 | bHLH |
| 4346047 | 2572 | 14.41486 | 45.52346 | 1.659053 | 0.00333 | XM_015792976.1 | mTERF |
| 1.07E+08 | 1002 | 386.7068 | 34.29638 | -3.49511 | 5.60E-20 | XM_015783507.1 | AP2-EREBP |
| 4338909 | 2229 | 3230.819 | 471.2725 | -2.77727 | 3.85E-21 | XM_015784903.1 | TUB |
| 4352653 | 1323 | 2087.034 | 398.1242 | -2.39016 | 3.38E-08 | XM_015764706.1 | C2H2 |
| 4340011 | 2483 | 71.28231 | 264.6708 | 1.892583 | 5.85E-05 | XM_015787555.1 | NAC |
| 4351545 | 1273 | 276.8504 | 30.87948 | -3.16439 | 6.75E-09 | XM_015763955.1 | NAC |
| 4325059 | 1855 | 2633.088 | 232.9168 | -3.49887 | 5.91E-10 | XM_015770348.1 | C2H2 |
| 4339739 | 1562 | 2905.429 | 463.0921 | -2.64938 | 1.43E-11 | BGI_novel_T006847 | WRKY |
| 4327914 | 2004 | 9.045625 | 46.64541 | 2.366443 | 0.00054 | XM_015771623.1 | MYB |
| 4348531 | 840 | 4245.5 | 494.4087 | -3.10216 | 1.54E-05 | XM_015757976.1 | Tify |
| 4337480 | 1849 | 415.5486 | 1054.404 | 1.343338 | 3.30E-05 | XM_015781385.1 | TUB |
| 4339005 | 2404 | 285.1069 | 133.3855 | -1.0959 | 0.000155 | XM_015781949.1 | MYB |
| 1.07E+08 | 2632 | 319.1583 | 8.65809 | -5.20408 | 8.48E-27 | XM_015786781.1 | MYB |
| 1.07E+08 | 1843 | 43.72486 | 7.251711 | -2.59206 | 1.53E-05 | XM_015779901.1 | EIL |
| 4336551 | 1108 | 14300.64 | 4489.341 | -1.6715 | 9.39E-13 | BGI_novel_T005593 | Trihelix |
| 4347486 | 1618 | 579.721 | 210.0671 | -1.46451 | 2.62E-06 | XM_015795892.1 | bHLH |
| 4327611 | 2050 | 1359.69 | 653.3082 | -1.05744 | 0.011179 | XM_015765748.1 | WRKY |
| 4331028 | 1686 | 1501.919 | 169.1786 | -3.15019 | 1.81E-16 | XM_015771101.1 | DBP |
| 4334181 | 1971 | 11.77364 | 46.44867 | 1.980077 | 0.000201 | XM_015772855.1 | bHLH |
| 4343296 | 3142 | 103.4741 | 240.9794 | 1.21964 | 0.000504 | XM_015792216.1 | FAR1 |
| 4328463 | 1421 | 18.24838 | 79.85727 | 2.129655 | 6.41E-06 | XM_015770578.1 | Trihelix |
| 4347215 | 2514 | 63.37973 | 168.1763 | 1.407881 | 0.002128 | BGI_novel_T010080 | C2H2 |
| 9269857 | 1083 | 922.0153 | 299.751 | -1.62103 | 8.49E-06 | XM_015764541.1 | WRKY |
| 4345697 | 1870 | 49.40354 | 129.6235 | 1.391641 | 7.85E-05 | XM_015795525.1 | AP2-EREBP |
| 4341978 | 4234 | 534.1782 | 235.029 | -1.18448 | 0.001608 | XM_015788007.1 | ARF |
| 4347257 | 1885 | 154.446 | 624.162 | 2.014818 | 4.17E-12 | XM_015757064.1 | bZIP |
| 4324327 | 2378 | 214.5962 | 469.4757 | 1.129426 | 4.04E-06 | XM_015766441.1 | bHLH |
| 4349457 | 1979 | 2478.561 | 330.8549 | -2.90523 | 6.47E-06 | XM_015757390.1 | NAC |
| 4342572 | 1848 | 282.3982 | 691.7697 | 1.292561 | 2.21E-09 | XM_015789121.1 | bHLH |
| 4346133 | 2218 | 5.216614 | 29.9022 | 2.519066 | 0.004651 | XM_015793891.1 | SBP |
| 4348646 | 1843 | 6334.444 | 882.6247 | -2.84335 | 4.14E-18 | BGI_novel_T010665 | HSF |
| 4330431 | 1550 | 434.4885 | 54.81697 | -2.98662 | 1.65E-29 | XM_015768271.1 | G2-like |
| 4344404 | 2276 | 59.95542 | 121.6954 | 1.021313 | 0.003815 | XM_015790995.1 | MYB |
| 4333457 | 4838 | 4.704766 | 37.89308 | 3.00974 | 4.45E-05 | XM_015777192.1 | bHLH |
| 4334170 | 1607 | 2552.706 | 608.3312 | -2.0691 | 1.88E-30 | XM_015775935.1 | WRKY |
| 9272089 | 2168 | 331.6532 | 124.0838 | -1.41836 | 0.000342 | XM_015778621.1 | ABI3VP1 |
| 1.07E+08 | 2318 | 196.3044 | 40.4953 | -2.27727 | 9.00E-07 | XM_015760343.1 | GRAS |
| 4351811 | 3159 | 34.57304 | 70.33604 | 1.024617 | 0.013762 | XR_001542244.1 | FAR1 |
| 4339599 | 2019 | 10866.48 | 1525.674 | -2.83237 | 2.50E-28 | XM_015784323.1 | Trihelix |
| 4326323 | 1829 | 212.1757 | 55.09852 | -1.94517 | 4.02E-09 | BGI_novel_T001032 | C3H |
| 4330115 | 1493 | 76.98871 | 169.4867 | 1.138453 | 0.011757 | XM_015767513.1 | MYB |
| 9272517 | 961 | 7135.507 | 526.2359 | -3.76123 | 7.14E-14 | XM_015779175.1 | C2H2 |
| 4334582 | 2131 | 265.0015 | 88.01822 | -1.59013 | 2.15E-06 | XM_015772884.1 | AP2-EREBP |
| 4345807 | 1606 | 943.6759 | 327.3511 | -1.52745 | 6.25E-18 | XM_015795440.1 | bZIP |
| 4347164 | 1325 | 448.4212 | 174.4671 | -1.3619 | 0.002188 | XM_015755158.1 | Tify |
| 4326690 | 1792 | 299.2611 | 65.97647 | -2.18138 | 1.38E-08 | XM_015770821.1 | WRKY |
| 4348906 | 1454 | 491.6211 | 22.75262 | -4.43344 | 1.18E-13 | XM_015757470.1 | MYB |
| 1.07E+08 | 1466 | 4.487486 | 27.89281 | 2.635914 | 0.000411 | XM_015787493.1 | bHLH |
| 4328300 | 1402 | 676.7367 | 298.4304 | -1.1812 | 4.14E-10 | XM_015767504.1 | C2C2-GATA |
| 1.07E+08 | 1391 | 3.843548 | 0.540778 | -2.82933 | 0.012689 | XM_015780747.1 | MYB |
| 4329950 | 1671 | 105.8608 | 350.6294 | 1.727779 | 2.15E-06 | XM_015770369.1 | C2C2-CO-like |
| 1.07E+08 | 1647 | 2062.448 | 784.1494 | -1.39516 | 7.44E-05 | XM_015768437.1 | AP2-EREBP |
| 4324664 | 1274 | 922.511 | 136.4272 | -2.75743 | 1.81E-06 | XM_015778465.1 | MYB |
| 4343521 | 2155 | 21353.99 | 1194.971 | -4.15946 | 1.75E-50 | XM_015791310.1 | GRAS |
| 4330189 | 1301 | 4871.07 | 1705.535 | -1.51401 | 5.89E-05 | XM_015767773.1 | AP2-EREBP |
| 4338037 | 2095 | 741.1171 | 2178.692 | 1.555689 | 1.44E-09 | XM_015785277.1 | C3H |
| 4327838 | 1840 | 778.2791 | 219.3763 | -1.82688 | 1.09E-05 | XM_015765911.1 | ULT |
| 4349649 | 4077 | 55.98765 | 3.73767 | -3.9049 | 1.69E-07 | XM_015760603.1 | NAC |
| 9267434 | 1770 | 201.137 | 46.1345 | -2.12426 | 7.40E-08 | XM_015779381.1 | NAC |
| 4351576 | 1426 | 866.9005 | 378.2645 | -1.19647 | 3.45E-11 | BGI_novel_T012037 | bZIP |
| 4327040 | 1818 | 95.7104 | 272.4167 | 1.509068 | 1.04E-06 | XM_015777960.1 | bHLH |
| 4343735 | 2297 | 12665.69 | 1908.584 | -2.73035 | 2.75E-27 | XM_015791259.1 | GRAS |
| 4325175 | 1977 | 1133.058 | 460.1137 | -1.30016 | 1.06E-11 | XM_015777848.1 | BSD |
| 1.07E+08 | 1629 | 14.00177 | 37.16337 | 1.408273 | 0.009135 | XM_015764142.1 | GRAS |
| 4351184 | 1365 | 197.8897 | 1753.775 | 3.147696 | 5.73E-60 | XM_015762473.1 | MYB |
| 4346379 | 1747 | 1635.228 | 676.2957 | -1.27377 | 5.27E-12 | XM_015757104.1 | MYB |
| 4343736 | 2359 | 546.968 | 1135.455 | 1.053743 | 0.000419 | XM_015791257.1 | WRKY |
| 4332697 | 2820 | 598.952 | 287.1497 | -1.06064 | 0.005504 | BGI_novel_T004031 | EIL |
| 4338925 | 1036 | 2063.093 | 123.9619 | -4.05684 | 1.14E-08 | XM_015782734.1 | MYB |
| 4347637 | 2128 | 8996.365 | 2368.843 | -1.92516 | 1.70E-23 | XM_015757040.1 | HSF |
| 4339778 | 2313 | 97.24931 | 365.6139 | 1.910561 | 4.59E-08 | XM_015782153.1 | Sigma70-like |
| 4334060 | 2692 | 26550.73 | 2408.608 | -3.46248 | 1.22E-12 | XM_015775745.1 | bHLH |
| 4329577 | 2382 | 256.548 | 103.2312 | -1.31335 | 5.28E-06 | XM_015767892.1 | E2F-DP |
| 4347431 | 2132 | 210.6519 | 845.7206 | 2.005321 | 4.82E-16 | XM_015757126.1 | bHLH |
| 4325196 | 2329 | 35.1576 | 9.347273 | -1.91122 | 0.011138 | XM_015792514.1 | LOB |
| 4343245 | 2110 | 1033.17 | 281.3925 | -1.87642 | 5.20E-21 | BGI_novel_T008433 | MYB |
| 4352838 | 1433 | 25.9263 | 65.42838 | 1.3355 | 0.002627 | XM_015762856.1 | C2C2-GATA |
| 4329526 | 2484 | 17513.79 | 690.2032 | -4.66533 | 4.05E-54 | XM_015767575.1 | HSF |
| 9270355 | 1352 | 6.752598 | 29.88165 | 2.145745 | 0.005283 | XM_015789929.1 | SBP |
| 4350492 | 2008 | 1302.337 | 515.2382 | -1.33779 | 3.84E-11 | XM_015761962.1 | C3H |
| 4331707 | 1778 | 1015.6 | 388.3875 | -1.38676 | 2.23E-05 | BGI_novel_T003579 | HSF |
| 4346973 | 2561 | 5.103602 | 29.53681 | 2.532926 | 0.000308 | XM_015755474.1 | G2-like |
| 4339348 | 1783 | 60.16866 | 182.5826 | 1.601465 | 0.001347 | XM_015782533.1 | C2C2-GATA |
| 4331187 | 2677 | 1415.466 | 677.8172 | -1.06231 | 1.13E-08 | XM_015769205.1 | NAC |
| 4338978 | 1328 | 7195.072 | 1358.69 | -2.40479 | 3.58E-17 | XM_015782051.1 | bHLH |
| 9268901 | 1562 | 36.69311 | 6.28113 | -2.54641 | 0.002464 | XM_015767011.1 | zf-HD |
