# Supplementary figures and images for: Transcriptome Sequencing and iTRAQ of Different Rice Cultivars Provide Insight into Molecular Mechanisms of Cold-Tolerance Response in Japonica Rice
Source: Rice (N Y). 2020 Jun 22;13:43. doi: 10.1186/s12284-020-00401-8 (PMC7310054; doi:10.1186/s12284-020-00401-8)

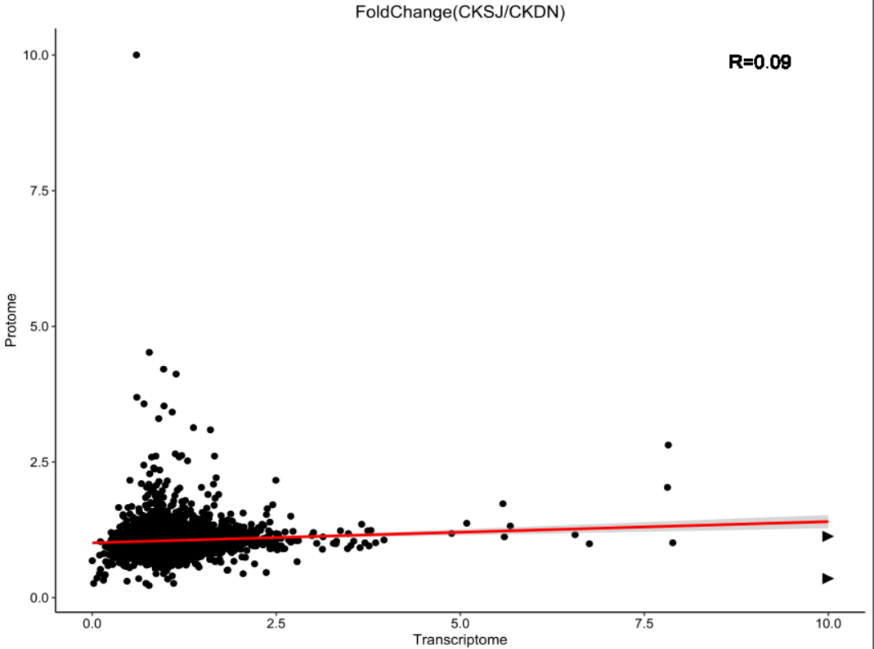

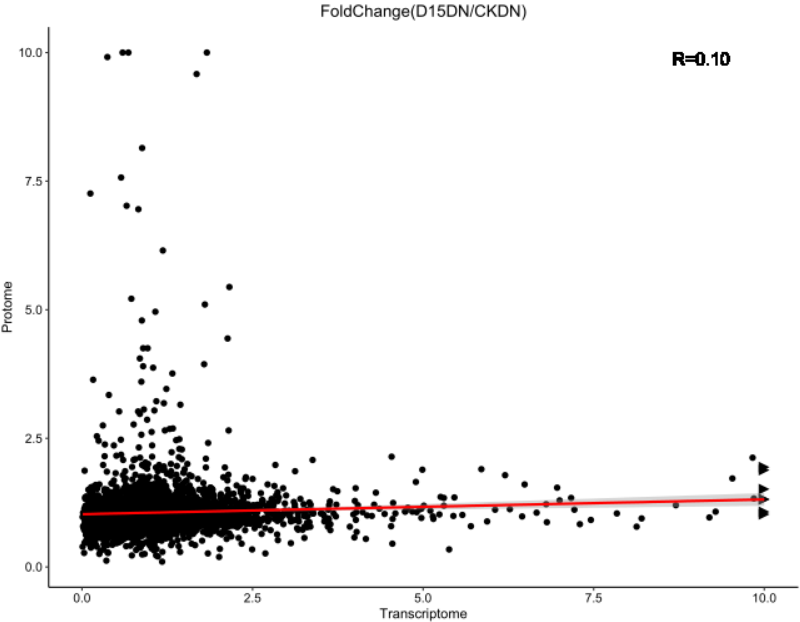


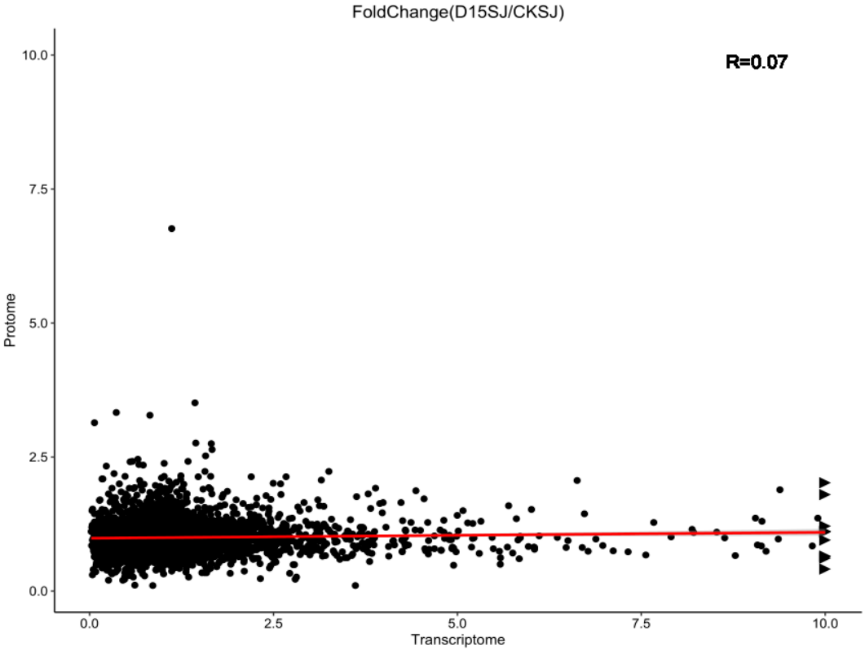

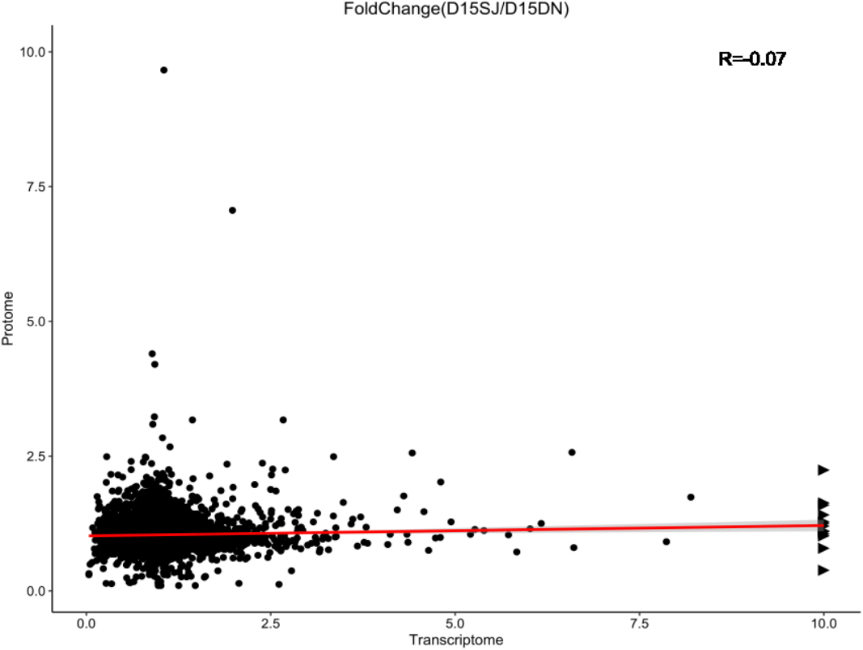


**Fig.** **S1**

Supplement: Supplementary file 3 — Additional file 3: Figure S1. Spearman correlations for the proteins and corresponding genes in each group. [file 12284_2020_401_MOESM3_ESM.docx]

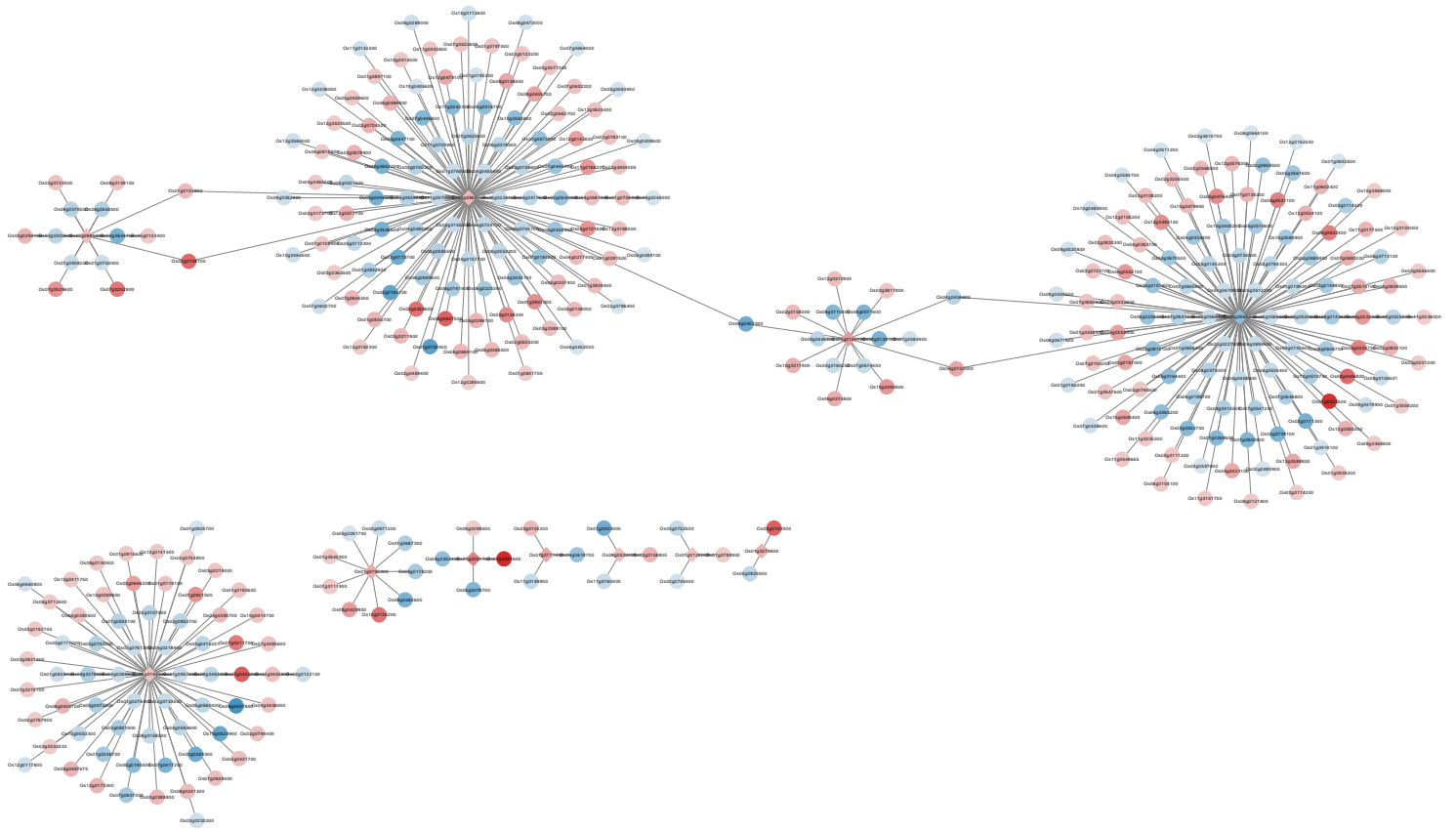

Supplement: Supplementary file 4 — Additional file 4: Figure S2. Targeted gene analysis of the TFs. [file 12284_2020_401_MOESM4_ESM.pdf]
